# Effects of immune challenge on expression of life-history and immune trait expression in sexually reproducing metazoans—a meta-analysis

**DOI:** 10.1186/s12915-020-00856-7

**Published:** 2020-10-07

**Authors:** M. Nystrand, D. K. Dowling

**Affiliations:** School of Biological Sciences, Monash University, Clayton, Victoria 3800 Australia

**Keywords:** Immune challenge, Phenotype, Context-dependence, Life-history trade-offs, Sexually dimorphic, Sex-specific

## Abstract

**Background:**

Life-history theory predicts a trade-off between investment into immune defence and other fitness-related traits. Accordingly, individuals are expected to upregulate their immune response when subjected to immune challenge. However, this is predicted to come at the expense of investment into a range of other traits that are costly to maintain, such as growth, reproduction and survival. Currently, it remains unclear whether the magnitude of such costs, and trade-offs involving immune investment and other traits, manifests consistently across species and sexes. To address this, we conducted a meta-analysis to investigate how changes in sex, ontogenetic stage and environmental factors shape phenotypic trait expression following an immune challenge.

**Results:**

We explored the effects of immune challenge on three types of traits across sexually reproducing metazoans: life-history, morphological and proximate immune traits (235 effect sizes, 53 studies, 37 species [21 invertebrates vs. 16 vertebrates]). We report a general negative effect of immune challenge on survival and reproduction, a positive effect on immune trait expression, but no effect on morphology or development time. The negative effects of immune challenge on reproductive traits and survival were larger in females than males. We also report a pronounced effect of the immune treatment agent used (e.g. whether the treatment involved a live pathogen or not) on the host response to immune challenge, and find an effect of mating status on the host response in invertebrates.

**Conclusion:**

These results suggest that costs associated with immune deployment following an immune challenge are context-dependent and differ consistently in their magnitude across the sexes of diverse taxonomic lineages. We synthesise and discuss the outcomes in the context of evolutionary theory on sex differences in life-history and highlight the need for future studies to carefully consider the design of experiments aimed at disentangling the costs of immune deployment.

## Background

Animals living in natural environments are constantly exposed to microbes, some of which are pathogenic, threatening the survival of their hosts [1]. Most models attempting to explain heterogeneity in host responses to pathogenic microbes are grounded on the assumption that the combined effort of producing, maintaining and activating the immune system is costly to the host [2, 3]. As a result of these costs, investment into immunity (defined here as the ability of an organism to resist or tolerate microbes) is predicted to be tightly linked to the expression of other fitness-related traits, and to be involved in the trade-offs between these traits [2–9]. Accordingly, theory predicts that individuals will be constrained in their ability to express optimal immune responses [10]. Moreover, if such trade-offs have an underlying genetic basis, then populations may be constrained in their capacity to evolve optimised immune defences, without incurring costs to expression of life-history traits [11, 12].

To date, many studies that have subjected individuals to immune challenges have demonstrated clear negative effects on the expression of life-history traits, such as reproductive capacity and survival, ranging across various insect species (e.g. *Drosophila melanogaster* [13–16], *Anopheles gambiae* [17], *Teleogryllus oceanicus* [18] and *Tribolium castaneum* [19]), to lizards [*Ctenophorus fordi*] [20] and birds (e.g. *Passer domesticus* [21], *Ficedula hypoleuca* [22])—also, see reviews in [3, 11, 23]. However, the magnitude of recorded effects are rarely equal across individuals within or between species, which has prompted evolutionary ecologists to seek to understand the intrinsic and extrinsic factors that might mediate the magnitude and pattern of responses to immune challenge [8, 24–26]. In particular, many studies have focused on the capacity for sex differences to occur in immune responses and disease expression, in humans and other vertebrates [27–29], as well as invertebrates [28, 30]. Indeed, evolutionary theory predicts that there should be sex differences in immune responses, given that optimal life-history strategies typically diverge between the sexes [31, 32]. Notwithstanding, predictions as to which sex should be more sensitive to immune challenge remain somewhat unclear. For example, in species that experience strong sexual selection, selection may favour high investment into early-life reproduction in males, at the expense of later life components of fitness, but sustained reproduction across life in females [31]. Under these conditions, females should benefit more than males through heightened investment into traits that enhance their immune response and thus promote somatic maintenance and survival [28, 31–35]. On the other hand, the per unit costs associated with many components of reproduction are typically envisaged to be larger in females (attributable to a larger gamete size and higher levels of resource provisioning to offspring both prior to- and post birth) than in males [32, 36, 37]. In addition, direct costs to females are two-fold; firstly, reproduction can cause direct harm to the soma, and second, resources that could be used to repair damage to the soma are instead invested into reproduction [36–39]. Accordingly, sex biases in direct reproductive costs might render females more sensitive to an active immune challenge [37–39], as well as reduce the capacity of their immune system to resist a challenge [2, 11, 40]. Hence, understanding whether sex differences in general life-history will manifest into consistent sex differences in patterns of immune investment across metazoans is complex.

In 2009, Nunn et al. investigated patterns of sex difference in the expression of two particular traits involved in the innate immune response (phenoloxidase expression and haemocyte number) in insects, reporting a signature of female bias for the expression of phenoloxidase, but not for haemocyte counts across studies [28]. More recently, another study set out to also investigate patterns of sex difference in immune traits, investigating several different traits (including measures of innate immunity, such as those involved in melanisation and encapsulation responses, and acquired immunity via the antibody response to antigen) across broad taxonomic groups of metazoans [30]. The authors reported a general tendency of heightened female immunocompetence, albeit not statistically significant when accounting for phylogenetic differences [30]. While highly insightful, these findings beg the question of whether any signatures of sex bias at the level of expression of immune traits translate into pronounced female biases when it comes to the expression of key components of life-history, such as reproduction and survival.

The general lack of a consistent sex bias in the expression of immune traits across studies and taxa suggests that other, unaccounted for, factors may be involved in moderating the magnitude of sex differences in immunocompetence. For example, immune responses are likely to vary with the type of immune system involved (invertebrates have innate immune systems only, whereas vertebrates have innate and acquired systems), the ontogenetic stage at which an immune response in measured, the mating status of the individuals measured, or the type of immune challenge used. It is well established that in vertebrates that are equipped with an acquired immune system, there is a sizeable shift in immune function capability from infancy to adulthood [25, 41, 42]. Similar shifts related to ontogenetic stage are recorded for invertebrate innate immune systems, whereby development-specific stages of immune function have been recorded (e.g. lamellocytes and crystal cells in *Drosophila* flies are only found in the larval stages [43, 44]; haematopoiesis only occurs in pupal and larval stages in several insect species [43–48]).

It must also be noted that methodological differences are likely to generate much variation in results across studies, given that studies often utilise different techniques by which to elicit immune responses. For example, where some studies utilise living, replicating pathogens to elicit an immune response, others challenge individuals with immobilised pathogens (e.g. heat-killed pathogens or lipopolysaccharide) that elicit an immune response without inducing the direct effects associated with pathogenicity of a replicating pathogen [49–52]. Indeed, mechanistic studies have demonstrated that patterns of host responses following exposure to live versus immobilised/dead pathogens can be directly opposite of each other [13, 53, 54]. Thus, while the type of challenge used is invariably informed by the particular question under test in the individual study, it may represent a large source of heterogeneity across studies exploring both general and sex-biased patterns of immune responses.

Motivated by the open questions outlined above, we set out to conduct a systematic meta-analysis, with two primary aims. The first aim was to quantify the magnitude and direction of effects of immune challenge, on expression of life-history traits (survival, reproduction, development times), proximate immune traits (i.e. traits directly related to immune function), and morphological indicators of structural size, across gonochoristic metazoans (animals with separate sexes). We predicted to find greater effects of immune challenge on expression of life-history and proximate immune traits compared to morphological traits, simply because morphological traits are usually set early in life (by adulthood) [55] and should therefore exhibit little—if any—plasticity throughout life. The second aim was to establish the degree to which variation across studies and taxa in the effects of immune challenge on the above traits could be explained by inclusion of moderating factors (i.e. moderators). First, we explored the effect of sex and ontogenetic stage at the time of immune challenge (i.e. using a composite trait consisting of juveniles, adult females, and adult males, henceforth referred to as “life-history status”) on expression of life-history, proximate immune, and morphological traits. Second, we explored the type of treatment agent used to trigger the immune system (i.e. whether the challenge involved a living, replicating immune agent or an immobilised/dead, non-replicating agent). Third, we investigated whether organismal responses to immune challenge differed between studies of invertebrates and vertebrates, given the different immune systems between these groups. In addition, we also extracted a subset of data (invertebrates only) in which we explored the effect of host mating status on the response to immune challenge. This analysis was motivated by prior evidence suggesting that mating activity can alter immune function efficiency and expression of fitness-related traits [11, 40].

## Results

### Data extraction

We specifically focused on studies that assessed the effect of active immune deployment following a challenge, as opposed to effects associated with immune maintenance at background levels (in the absence of a challenge). For this reason, the main criterion for inclusion of studies in our meta-analysis was that they had to have administered an active immune challenge, and this had to be associated with a procedural control (see Additional file 1, Table S1 for full search string and Table S3 for more details on the immune challenges included). The meta-analysis was limited to gonochoristic bilaterians (bilaterian animals with two distinct sexes).

**Fig. 1:**
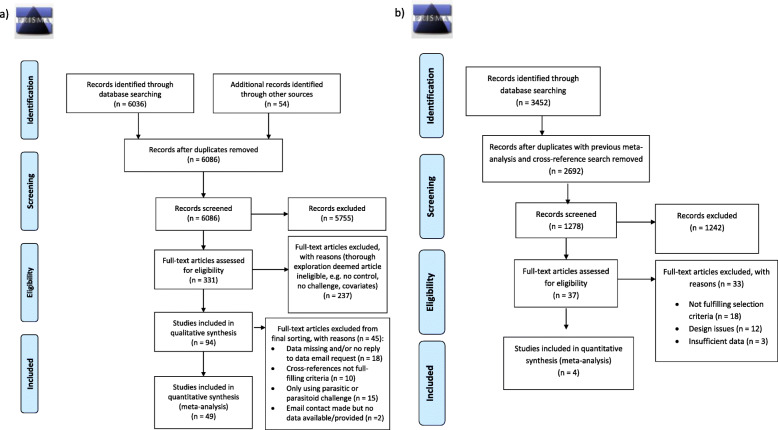
Diagram outlining search process (PRISMA): **a** original search and **b** add-on search specifically aimed at the host-pathogen literature

The article extraction generated a total number of 8778 studies (from the two database searches [backwards and forward searches]), after duplicates were removed and including additional information received from research groups that were contacted directly (see Fig. 1 for more details). Of these, 368 studies were screened more thoroughly (Fig. 1 and Additional file 1, Table S2), resulting in a final number of 53 individual studies (*N*_studies_ in each analysis [same study may contain data on more than one trait]: survival = 23, reproduction = 29, immune trait expression = 16, morphology = 31 and development = 6). In sum, these studies contained a total of 235 effect sizes [ES] (*N*: survival = 51, reproduction = 80, immune trait expression = 54, morphology = 31, and development = 18), ranging across 37 species in total (representation in each study was *N*: survival = 21, reproduction = 22, immune trait expression = 13, morphology = 14, and development = 5). The majority of species belonged to invertebrates, accounting for 189 ES across 21 invertebrate species, compared to 47 ES across 16 vertebrate species (*N*_ES_ vertebrates/invertebrates for each trait analysed: survival = 8/43, reproduction = 8/44, immune trait expression = 7/47, morphology = 12/19, development = 11/13) (see Additional file 1, Table S2, and the raw file data available from figshare, for a list of included studies). Out of the 48 authors that were contacted, we received complementary data from 16 groups; however, only eight of these studies ended up being adequate for use in the analyses (Fig. 1).

### Results from meta-analytic tests (intercept models)

The meta-analytic models for three of the five traits (i.e. survival, reproduction and immune trait expression) supported a general effect of immune challenge on host trait expression. This effect was most pronounced for survival, in which a clear negative effect of immune challenge was recorded in both the model accounting for phylogeny (i.e. model in which a phylogenetic correlation matrix had been fitted—from here on referred to as the “phylogenetic model”) and the model that did not account for phylogeny (i.e. model lacking the phylogenetic correlation matrix; from here on referred to as the “non-phylogenetic model”; Table 1). Likewise, reproduction was negatively affected by immune treatment, regardless of whether phylogeny was accounted for or not (both models were identical: Table 1). Traits directly measuring proximate immune traits showed a positive increase following immune challenge (indicating their upregulation), and this increase was independent of phylogeny (Table 1). In contrast, neither morphology, nor development times appeared sensitive to immune challenge (Table 1). All models showed a slightly better fit when phylogeny was not accounted for (lower AIC where dAIC ≤ 2 in most cases; Table 1). Forest plots of the full data, displaying each data point, for each trait are shown in Additional file 1, Fig. S2, S6, S10, S18 and S22. Note that the same study may have contributed data for several different traits.

**Table 1:** Results of meta-analytic models, accounting for phylogeny (“Phylo”) vs. those not accounting for phylogeny (“Non-phylo”). Note that survival was analysed using ln odds ratios [lnOR], but for clarity, raw odds ratios [OR] are also displayed

Model	Effect size	SE	CI lower	CI upper	AIC
***Survival lnOR (OR)***					
Phylo	− 1.252 (0.286)	0.358	− 1.953 (0.142)	− 0.551 (0.577)	167.86
Non-phylo	− 1.252 (0.286)	0.358	− 1.953 (0.142)	− 0.551 (0.577)	165.86
***Reproduction (Hg)***					
Phylo	− 0.132	0.072	− 2.273	− 0.010	124.43
Non-phylo	− 0.132	0.072	− 2.273	− 0.010	122.43
***Immune trait exp. (Hg)***					
Phylo	0.824	0.322	0.192	1.462	160.25
Non-phylo	0.824	0.322	0.192	1.462	158.25
***Morphology (Hg)***					
Phylo	0.040	0.265	− 0.479	0.558	73.017
Non-phylo	0.038	0.128	− 0.213	0.289	69.113
***Development time (Hg)***					
Phylo	− 0.152	0.344	− 0.827	0.522	29.21
Non-phylo	− 0.061	0.169	− 0.392	0.270	27.90

All our models displayed high heterogeneities, with values of total *I*^*2*^ ranging from a minimum of 71.27% (reproduction, both models) to a maximum of 96.84 (survival, Table 2). Such large heterogeneities in a meta-analytic model indicate that there are additional and unaccounted factors affecting the outcome of the analysis [56].

**Table 2:** Quantified heterogeneities (*I*^*2*^%) for meta-analytic models, estimating variance accounted for by shared group identity, shared study identity, shared phylogeny, residual against sampling error (i.e. variance due to effect size), total variance and phylogenetic signal (*H*^2^), for each of the traits measured and for models accounting for phylogeny vs. those that did not

	***I***^***2***^_**GROUP**_	***I***^***2***^_**STUDY**_	***I***^***2***^_**PHYLOG**_	***I***^***2***^_**RESIDUAL**_	***I***^***2***^_**TOTAL**_	***H***^**2**^
***Survival***						
Phylo	–	95.70	4.17^–07^	1.13	96.83	4.31^–09^
Non-phylo	–	95.70	–	1.13	96.83	–
***Reproduction***						
Phylo	1.41^–08^	51.57	1.63^–07^	19.70	71.27	2.28^–09^
Non-phylo	3.16^–08^	51.57	–	19.70	71.27	–
***Immune trait exp.***						
Phylo	8.48^–09^	80.27	3.84^–07^	13.64	93.90	4.08^–09^
Non-phylo	1.95^–08^	80.27	–	13.64	93.90	–
***Morphology***						
Phylo	28.34	8.06^–08^	45.07	11.44	84.85	0.53
Non-phylo	35.04	23.22	–	21.04	79.30	–
***Development time***						
Phylo	9.97^–09^	4.22^–12^	71.09	20.64	91.73	0.77
Non-phylo	2.42^–08^	44.09	–	41.84	85.92	–

The contribution of phylogenetic heterogeneity (*I*_*2 PHYLOG*_) was more variable across traits, showing close to no effect in the models exploring survival, reproduction and immune traits (< 0.001%), to moderate and strong levels in morphology and development times (45.07% and 71.09% respectively, Table 2). However, despite this strong signal, the overall models did not perform better when phylogeny was accounted for based on AIC values of the models.

### Results from meta-regression: testing the effect of moderators on the models

Initially, we tested the effect of two moderators, life-history stage at the time of challenge (sex and ontogenetic age combined) and treatment agent (replicating or non-replicating immune challenge; more details in Additional file 1, Table S3) on four different traits: survival, reproduction, proximate immune traits and morphology. We also compiled data on how immune challenge influenced development times; however, all data on this trait came from juvenile individuals, and thus, life-history stage was not included in this test.

**Fig. 2:**
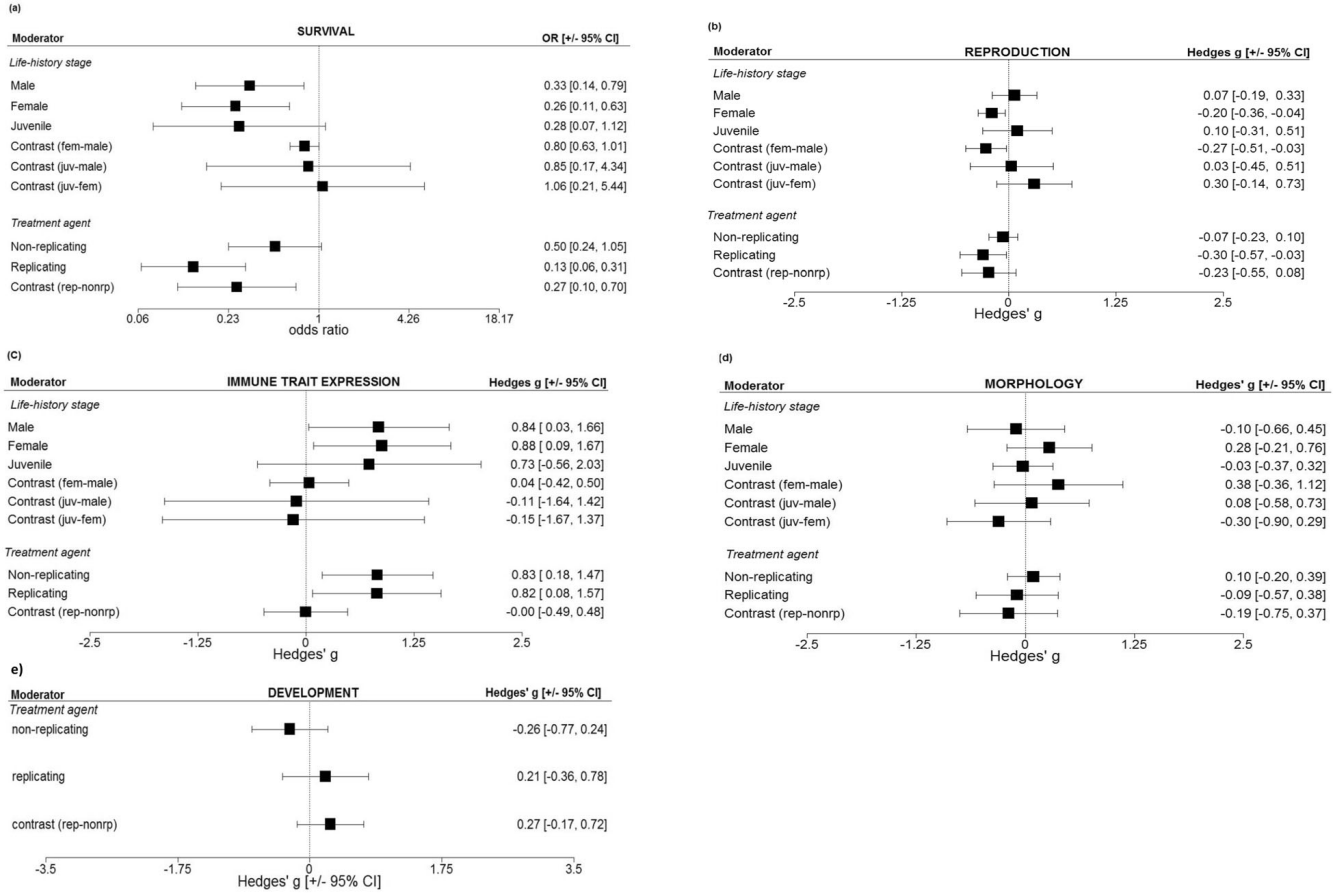
Univariate tests showing the results of each moderator in isolation, across all traits: **a** survival (*N*_ES_ = 51, *N*_studies/species_ = 23/21), **b** reproduction (*N*_ES_ = 80, *N*_studies/species_ = 29/22), **c** immune trait expression (*N*_ES_ = 54, *N*_studies/species_ = 16/13), **d** morphology (*N*_ES_ = 31, *N*_studies/species_ = 15/14), and **e** development times (*N*_ES_ = 18, *N*_studies/species_ = 6/5). Note that we have combined the two separate univariate tests into one figure

**Fig. 3:**
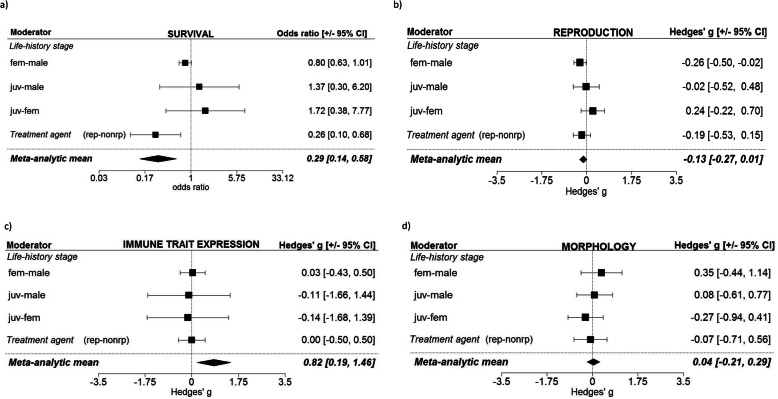
Results from meta-regression models containing both main moderators for each trait: **a** Survival (*N*_ES_ = 51, *N*_studies/species_ = 23/21), **b** reproduction (*N*_ES_ = 80, *N*_studies/species_ = 29/22), **c** immune trait expression (*N*_ES_ = 54, *N*_studies/species_ = 16/13), **d** morphology (*N*_ES_ = 31 *N*_studies/species_ = 15/14). Note that development is not shown because it is the same as in Fig. 2e, as there was only sufficient data to test one moderator

The analysis exploring the effects of life-history stage at time of challenge showed that both adult females and males had significantly reduced survival following an immune challenge, but juveniles did not (although juvenile responses were in the same direction as that recorded for adults; Fig. 2a and Additional file 2, Table A2.S1_1). Moreover, there was a clear signature of sex differences, whereby females were the more sensitive sex in both the univariate (Fig. 2a and Additional file 2 A2.S1_1) and the bivariate model in which the treatment agent was included (Fig. 3a and Additional file 2 A2.S6; this effect, however, was not strong enough to generate a significant interaction, Additional file 1, Table S4). Reproduction was also negatively affected by immune challenge. The magnitude of this response was female-biased (the effect on females was more negative than on males) and consistent across both the univariate model and the full meta-regression model (Fig. 2b and Additional file 2, Table A2.S2_1; Fig. 3b, Additional file 2, Table A2.S7). We did not detect a statistically significant effect of immune challenge on survival or reproduction in animals that were challenged as juveniles (Fig. 2a, b and Additional file 2, Table A2.S1_1, Table A2.S2_1; Fig. 3a, b and Additional file 2, Table A2.S6, Table A2.S7), nor did phylogenetic interdependence qualitatively influence these results (Additional file 1, Table S8-S11 and Table S14-S16).

Likewise, while there was an increase in proximate immune trait expression, in both females and males, following an immune challenge, there was no difference between the sexes, nor was there a difference between the responses in juveniles versus adult females or males (Fig. 2c and Additional file 2, Table A2.S3_1; Fig. 3c and Additional file 2, Table A2.S8, Additional file 1 Table S17). The juvenile responses aligned with the overall pattern of adult responses, suggesting that the lack of a significant response in this group may have been largely driven by a lack of statistical power. Further partitioning of these immune traits directly into their individual components (e.g. antimicrobial activity, phenoloxidase, number of haemocytes, phytohaemagglutinin) revealed that these individual components were either unaffected or positively upregulated following a challenge (Additional file 1, Fig. S12a-b). Due to limitations in the sample size associated with each immune trait component, a closer exploration of these patterns across all data was not possible. However, we were able to conduct an analysis that was limited to invertebrates only, in which a subset of the immune data containing high enough within-level replication was analysed (i.e. PO and antimicrobial activity responses). This revealed that antimicrobial activity increased following an immune challenge whereas PO remained unchanged (Additional file 1, Table S25, Fig. S13). The influence of age and sex showed the same patterns in the subset of invertebrate immune data as they did in the full data set (that contained both vertebrate and invertebrate data), whereby both adult males and females, but not juveniles, showed an upregulation of immune traits in response to an immune challenge (Fig. 2c and Additional file 2, Table A2.S3_1; Additional file 1, Figure S14-S15). Again, while the analysis of the subset of data used here cannot be extrapolated to the full data set (since the data subset used was limited to invertebrates), we note that models containing the phylogenetic matrix were almost identical to models not accounting for phylogeny, suggesting that phylogeny had a limited effect on the results (Additional file 1, Table S21–24). Finally, the expression of morphological traits following an immune challenge did not vary according to life-history status (Fig. 2d and Additional file 2, Table A2.S4_1; Fig. 3d and Additional file 2, Table A2.S9; Additional file 1, Table S27, S28, S30).

The analysis exploring the effect of immune challenge on host response showed that challenges consisting of a live and replicating treatment agent exerted a significant negative effect on both survival and reproduction when explored in isolation, i.e. univariate regression (Fig. 2a, b and Additional file 2, Table A2.S1_2, Table A2.S2_2; Additional file 1, Table S9, Table S15). Only for survival, however, was there a consistently significant difference between a live, replicating immune challenge versus a non-replicating immune challenge consistent across both the univariate and the full model (Fig. 2a, Fig. 3a, and Additional file 2, Table A2.S1_2; Additional file 1, Table S4, S6, S7, S9). In contrast, there was little evidence that the treatment agent influenced the magnitude of expression of proximate immune traits (Fig. 2c, Fig. 3c, and Additional file 2, Table A2.S3_2, Table A2.S8), or shaped morphological traits or development times (Fig. 2d, e and Additional file 2, Table A2.S4_2, Table A2.S5_1; Fig. 3d, e, and Additional file 2, Table A2.S9; Additional file 1, Table S27, S29-S31). Note that only two of the traits—survival and proximate immune trait expression—contained sufficient data to test for an interaction between life-history status and treatment agent; these analyses, however, did not indicate that the effect of the treatment agent varied according to life-history status for either trait (Additional file 1, Table S4, S7, S11, S17, S20, S24).

There was no significant difference in effect size associated with immune challenge between studies of vertebrates and invertebrates (Additional file 1, Table S5, S12, S18, S26 and Fig. S3, S7, S11, S19). The addition of this moderator (i.e. “animal kingdom”) to the full models, including the models on survival and immune trait expression that contained the interaction between life-history status and treatment agent, did not qualitatively alter the results (Additional file 1, Table S6, S7, S13, S19, S27).

The high heterogeneities recorded for the meta-analytical (intercept) models were not substantially altered by the inclusion of moderators (Table 3), suggesting that, overall, the moderators only exerted a limited effect on the models (note however, moderators may still be biologically relevant, despite having high heterogeneities, and in fact, in ecology, it is not uncommon with *I*^*2*^ values of up to 92% [57, 58]). Indeed, the estimation of the amount of variance that was explained by the full meta-regression models (*R*^2^) showed a moderate impact of the fixed factors for survival (*R*^2^_marginal_ = 11.09%) and reproduction (*R*^2^_marginal_ = 15.51%), but a high level of conditional variance (i.e. fixed and random effects) for both traits (*R*^2^_conditional_ = 99.32 vs. *R*^2^_conditional_ = 80.97). In contrast, while the conditional variances were high in the full models exploring proximate immune traits and morphology (*R*^2^_conditional_ = 88.14% vs. *R*^2^_conditional_ = 83.83%), the fixed effects accounted for a minimal part of the variance (*R*^2^_marginal_ = 0.14% vs. *R*^2^_marginal_ = 4.52%).

**Table 3:** Quantified heterogeneities (*I*^*2*^ %) for full meta-regression models (including life-history status and treatment agent only), estimating variance accounted for by shared group identity, shared study identity, shared phylogeny, residual against sampling error (i.e. variance due to effect size), total variance and phylogenetic signal (*H*^2^), for each of the traits measured and for models accounting for phylogeny vs. those that did not

	***I***^***2***^_**GROUP**_	***I***^***2***^_**STUDY**_	***I***^***2***^_**PHYLOG**_	***I***^***2***^_**RESIDUAL**_	***I***^***2***^_**TOTAL**_	***H***^**2**^
***Survival***						
Phylo	–	96.32	4.80^–07^	1.76^−10^	96.32	4.99^–09^
Non-phylo	–	96.32	–	9.88^–09^	96.32	–
***Reproduction***						
Phylo	1.14^−08^	57.14	6.40^–08^	16.34	73.48	8.71^–10^
Non-phylo	2.95^–08^	57.14	–	16.34	73.48	–
***Immune trait exp.***						
Phylo	1.89^–09^	80.67	4.51^–07^	13.82	94.49	4.78^–09^
Non-phylo	7.63^–11^	80.67	–	13.82	94.49	–
***Morphology***						
Phylo	24.46	6.70^–08^	52.82	9.65	86.92	0.61
Non-phylo	39.10	28.09	–	14.77	81.96	–
***Development time***						
Phylo	1.72^–09^	1.97^–11^	63.63	26.77	90.40	0.70
Non-phylo	1.81^–08^	54.67	–	33.42	88.09	–

### Effects of mating status on organismal response to an immune challenge

**Fig. 4:**
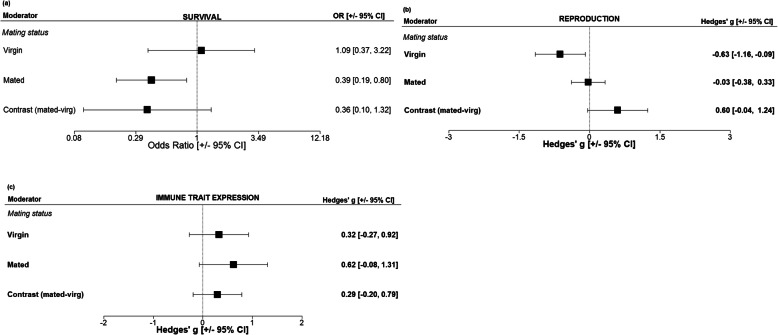
Subset analysis exploring the effects on mating on **a** survival (*N*_ES_ = 29, *N*_studies/species_ = 11/8, **b** reproduction (*N*_ES_ = 40, *N*_studies/species_ = 14/10), and **c** immune trait expression (*N*_ES_ = 29, *N*_studies/species_ = 6/4), following an immune challenge. Data was insufficient to test any interactions

We assessed the influence of mating status on moderating the effects of immune challenge for a subset of data in which all included species were invertebrates. We found an effect on survival following an immune challenge, whereby mated animals were associated with lower survival compared to virgin animals (Fig. 4a and Additional file 2, Table A2.S10). This effect showed the same signature but was slightly eroded when the test was limited to females only (Additional file 1, Figure S24a). In contrast, reproductive output was negatively affected by an immune challenge only when it had been administered to an individual before mating (i.e. in the virgin state; note that because of low sample sizes in males, this test could only be conducted in females; Fig. 4b and Additional file 2, Table A2.S11, Additional file 1, Fig. S24b). Finally, there was no effect of mating status on the response of proximate immune traits to immune challenge, regardless of whether the data contained both sexes or females only (Fig. 4c and Additional file 2, Table A2.S12, Additional file 1 Fig. S24c).

### Publication bias

**Fig. 5:**
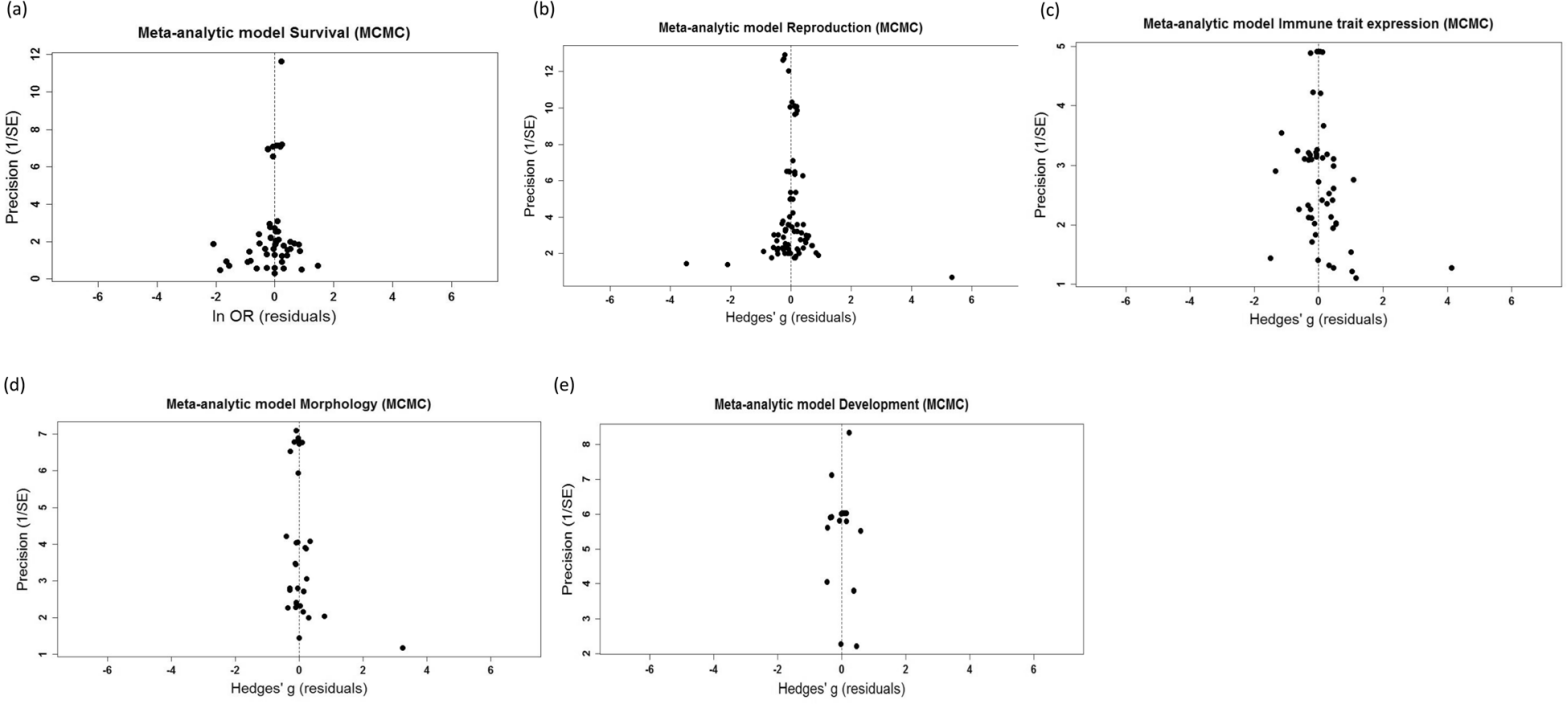
Funnel plots from meta-analytic models of **a**) survival, **b**) reproduction, **c**) immune trait expression, **d**) morphology and **e**) development times. The *X*-axis shows the residual values from the non-phylogenetic meta-analysis model, and the *Y*-axis shows the inverse of the standard error (precision). The dashed line indicates no effect (i.e. effect size of zero). Publication bias is indicated by asymmetry in the spread of the effect size residuals (e.g. less points on one side of the funnel)

**Fig. 6:**
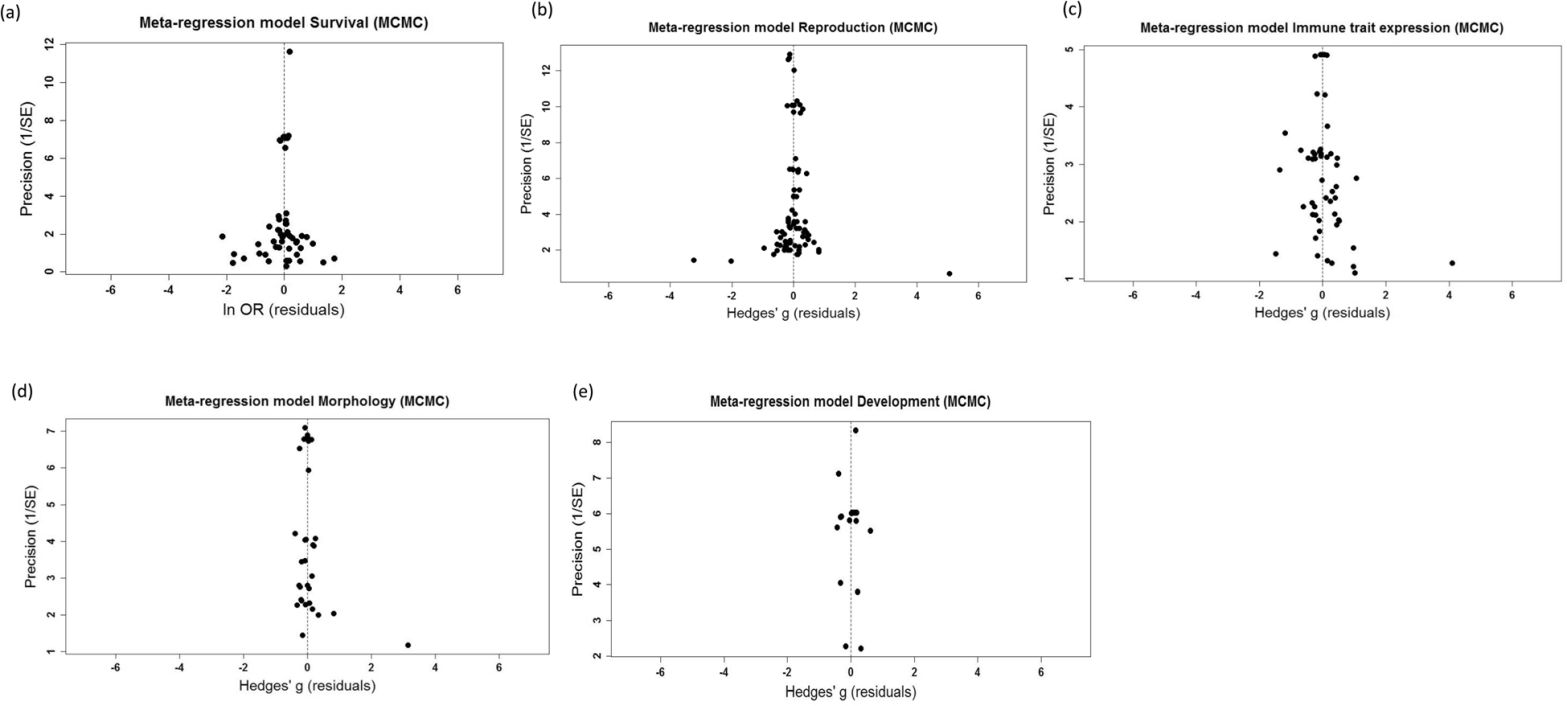
Funnel plots from full meta-regression models of **a**) survival, **b**) reproduction, **c**) immune trait expression, **d**) morphology and **e**) development times. The *X*-axis shows the residual values from the non-phylogenetic meta-analysis model, and the *Y*-axis shows the inverse of the standard error (precision). The dashed line indicates no effect (i.e. effect size of zero). Publication bias is indicated by asymmetry in the spread of the effect size residuals (e.g. less points on one side of the funnel)

Visual inspection of funnel plots did not suggest any obvious problems with asymmetry in the meta-analysis associated with any of the focal traits (i.e. scatter plots of meta-analytic funnel plots had no obvious bias in cluster of data points on either side of the mean, and the corresponding residual funnel plots were clustered symmetrically around the vertical “no effect” line; Fig. 5; more details in Additional file 1, Fig. S4, S8, S16, S20, S23). This conclusion was supported by the results of Egger’s regressions run on the model residuals for each trait, none of which were significantly different from zero (Table 4). Accordingly, we also conducted trim-and-fill analyses on the model residuals, which were consistent with the previous results suggesting limited or no asymmetry in the data (Table 4). The results of the meta-regressions did not alter these conclusions, showing no obvious sign of asymmetry (Fig. 6).

**Table 4:** Results of Egger’s regression and trim-and-fill analysis of residuals from the meta-analytic model (residuals generated in MCMCglmm)

	Egger’s regression				Trim and fill					
***Model***	***Est.***	***SE***	***t-value (d.f.)***	***P value***	***Missing studies***		***SE***		***P value***	
					*LO*	*RO*	*LO*	*RO*	*LO*	*RO*
***Survival***										
Phylo	− 0.320	0.210	− 1.525 (50)	0.134	4	3	4.535	2.828	–	0.063
Non-phylo	− 0.309	0.210	− 1.471 (49)	0.148	4	3	4.559	2.828	–	0.063
***Reproduction***										
Phylo	0.088	0.263	0.335 (78)	0.738	2	0	5.413	1.414	–	0.500
Non-phylo	0.125	0.266	0.471 (78)	0.639	1	0	5.357	1.414	–	0.500
***Immune trait exp.***										
Phylo	0.908	0.052	1.731 (52)	0.089	9	0	4.849	1.414	–	0.500
Non-phylo	0.931	0.523	1.771 (52)	0.082	11	0	4.854	1.414	–	0.500
***Morphology***										
Phylo	0.754	0.397	1.874 (29)	0.068	5	1	3.707	2.000	–	0.250
Non-phylo	0.803	0.407	1.974 (29)	0.058	4	2	3.681	2.450	–	0.125
***Development time***										
Phylo	− 0.113	1.421	− 0.079 (16)	0.938	0	0	2.669	1.414	–	0.500
Non-phylo	0.203	1.490	0.136 (16)	0.893	2	1	0.035	2.000	–	0.250

## Discussion

Our results support the presence of general physiological costs across both vertebrate and invertebrate taxa following immune challenge, as indicated by negative effects on the core facets of organismal life-history—survival and reproduction. Such costs are supported by research showing increasing resting metabolic rates following an immune system activation in the range of 8 to 57% across mammals, including humans [3, 59], and up to 25–28% in insects [60]. A notable feature of our meta-analyses is that all data came from standard laboratory or wild populations, which had not been experimentally manipulated in any other way, over-and-above the administration of an immune challenge. Thus, responses to immune challenge were measured under benign (control) conditions only, at least for the laboratory-based studies, which made up the majority of samples (*N*_ES lab_ = 213 vs. *N*_ES wild_ = 22). Previously, it has been proposed that the severity of immune costs may be limited in natural populations because individuals should be able to compensate for these costs under non-stressful conditions, for example, where nutritional resources are abundant [61]. Indeed, a general context dependence in host response to immune stress has been well documented in both theoretical [24, 62] and empirical models, for example, following dietary stress or climate change [3, 8, 35, 61]. The results of our meta-analysis, however, suggest that longer-term costs of immune challenge are likely to manifest, even in the absence of environmental stress as an exacerbating factor. Examples of such long-lasting costs include damage associated with autoimmunity in vertebrates (i.e. when the immune system attacks self-antigens) [63, 64], self-harm and auto-reactivity in invertebrates [65], or costs introduced by an increased production of reactive oxygen species that attack nucleic acids, proteins or lipids [26]. Interestingly, such production of reactive oxygen species may also be generated by reproduction itself, driven by increased physiological demands requiring higher levels of oxidative metabolism [66, 67]. In this context, animals may have limited capacity to fully offset the costs of an immune challenge, even under benign conditions.

The results of the meta-regressions, which tested the influence of a range of moderators, illustrated some intriguing associations. Firstly, we observed a female-biased reduction in reproductive output following immune challenge; a result that aligns with theoretical expectations and a large body of empirical evidence that predict physiological and/or evolutionary trade-offs manifested between reproduction and immunity, and which are resolved differently across the sexes (i.e. females investing relatively more than males in immune response at the expense of immediate reproductive output) [11]. Secondly, we uncovered a signature of female bias in survival responses to immune challenge, whereby females tended to suffer greater reductions in survival than males. This finding was notable, because it is seemingly inconsistent with life-history theory predicting that sex differences in life-history will generally favour selection on females that increase investment into immunity relative to males, to augment their survival when faced with immune challenge [27, 28, 68]. Moreover, when reconciled with our observation that mated individuals suffered shorter survival post-challenge than virgins (from an analysis of a subset of data limited to invertebrates only), these findings support the prediction that female biases in the costs of reproduction may, at least in invertebrates, render females the more sensitive sex to the costs of immune deployment.

The lack of a sex-specific response in proximate immune trait expression in our study aligns with the results of a recent meta-analysis by Kelly et al. (2018), which tested specifically for sexual dimorphism in specific immune traits. In that study, the authors reported a pattern of an overall marginal female bias (effect larger in females than males) in expression of proximate immune traits across all of the analysed studies. This effect was however, not statistically significant once phylogeny had been taken into account [30]. Interestingly, one of the subsets of data that did show evidence of a female-biased immunity in the study by Kelly and colleagues (2018) was one comparing induced (deployment) versus constitutive (maintenance) immunity in the innate branch of the immune system. This effect appears to have been largely driven by a more pronounced effect in immune challenged animals compared with animals that had only been sampled for background immunity [30]. Likewise, a previous meta-analysis by Nunn and colleagues (2009), comparing two insect proximate immune traits in females and males, showed inconsistent patterns of sex-biased immunity; while their results supported a female-bias in immune expression in one assayed trait (phenoloxidase [PO]), they failed to do so in another (haemocyte number). Nunn et al. (2009) also conducted a comparative analysis on sex-specific immunity in white blood counts in adult mammals, in which they, again, detected female-biased immunity in subsets of the data (i.e. two out of five white blood cell types were more abundant in females). Further details regarding key differences between the studies of Nunn et al. (2009), Kelly et al. (2018), and the analysis and results of immune trait expression that were tested in the current study are reported in the Additional file 1 (pp 68–70: “Detailed discussion on comparison with previous meta-analysis exploring proximate immune expression in animals”, [12, 69–74]).

The type of immune treatment agent used in the studies across the dataset, exerted a pronounced effect on several traits in our meta-analysis. Both replicating and non-replicating treatment agents had a general positive effect on proximate immune trait expression, but only replicating immune treatment agents (i.e. those involving live microbes) induced significant negative effects on life-history traits (survival and reproduction). Only for survival, however, was this effect large enough to generate a significant contrast between replicating and non-replicating treatments. These results are in perfect alignment with expectations, as we expected costs associated with the infection of a living, replicating immune agent to impose added costs to those induced by immune activation alone. A living microbe—as opposed to an immobilised, non-replicating elicitor of the immune system—has the potential to actively utilise host resources, invade tissues and organs and interfere with cellular processes [49–52], as well as interfere with host behaviour [75]. Also, the high speed of microbe replication under favourable conditions (for example, *E. coli* duplicates in 20–30 min [76]), suggests that the administered dose is likely to show an initial exponential increase, before the host manages to overcome the invading microbe. Thus, the effects of a living microbe will plausibly last longer—and peak later—compared to the effects associated with immune activation by a non-replicating microbe. Such time-delays could contribute to explaining the stronger effect of treatment agent on survival compared to reproduction, especially considering that reproductive output is often measured close to the time of the immune challenge treatment, while survival is often measured over a much longer stretch of time. Finally, it is possible that the negative effects of immune challenge on host survival are partly, or entirely, driven by direct pathological effects [50, 63–65], and such effects would be expected to be more pronounced in experiments administering a live, replicating immune challenge.

We failed to find any clear phylogenetic patterns in our data, nor did we find consistent differences between studies of vertebrates (which possess innate and acquired immune systems) and invertebrates (which possess innate systems only). However, we note a large bias towards invertebrates in our data set and are thus cautious not to over-interpret the significance of this lack of differences between the two groups. Note also, that while our analyses of morphology and development times displayed a modest to large phylogenetic signal, both models contained a limited number of species (14 and 5), which is less than what has been recommended as a minimum number (i.e. 20 species or more) required to conduct an accurate appraisal of phylogenetic signals [77, 78].

Across all data, there was an overall lack of clear phenotypic response to immune challenge when animals had been challenged as juveniles. Indeed, the point value for survival post-challenge in juveniles was similar to that of adults. Yet, juveniles seemed more robust to challenge when it came to effects on later reproduction following transition to adulthood. This is likely an artefact of a time delay built into these experiments, given reproduction cannot be measured until juveniles have advanced through to adulthood and mated, by which time the effects of immune challenge have likely dissipated. Furthermore, juveniles might invest relatively more in somatic maintenance than adults given that fewer resources are directly allocated to reproduction performance at this stage [79]. It is also possible that some or all of the juveniles tested were influenced by residual maternally inherited immune protection, which aided in boosting their immune system [80–84], hence providing them with an extra means of protection at that stage. In addition, at the time of immune challenge, juveniles did not have the added cost of mating faced by many adults [85, 86]. However, while there is plenty of explanations supporting the notion that juveniles may be more resilient to effects of immune challenge than adults, it is possible that the overall lack of a pronounced effect in juveniles is a statistical artefact of greater variation during this ontogenetic stage, whereby some outcomes (e.g. in immune trait expression) were reinforced by lower statistical power due to lower number of data points in this age class.

## Conclusions

This meta-analysis provides new and general insights into costs associated with immune deployment, by showing that these costs are context-dependent on the type of immune challenge used, type of traits measured, and the conditions under which the costs are measured. Moreover, our study reveals variation in the sensitivity of each sex to immune challenge, manifesting in sex differences in survival and reproductive outcomes whereby females exhibited larger negative effects. These results emphasise the importance of including both sexes into experimental designs in future studies that seek to explore general responses to immune deployment, in acknowledgement that sex differences are likely to affect the outcomes and conclusions of these studies. Moreover, ecological studies that specifically aim to establish how the actual trade-off between life-history traits and immunity change across ecological contexts should measure the effects across sexes, and carefully consider the experimental designs adopted to facilitate future comparisons across populations and species. While an increasing number of studies in ecology and evolution now acknowledge sexual dimorphism in immune responses (e.g. [35, 87–90]), other areas of science still tend to lag behind; for example, less than 10% of publications in the field of immunology account for sex differences in their analysis [27]. Finally, while challenging, future studies should also seek to fully resolve the mechanistic basis of these sex differences in response to immune deployment, to gain a full understanding of what process that drive sexual dimorphism in immunity.

## Methods

### Literature search and study inclusion criteria

We conducted two separate literature searches, both of which followed the procedure outlined in PRISMA [91] (the exact search string is outlined in the supplementary material Additional file 1, Table S1, p5), including scanning review papers, conducting backwards and forwards searches, exploring our personal existing libraries and contacting a number of researchers directly (Fig. 1a). The first search generated 6086 papers after duplications were removed (Fig. 1a). However, in this process, we noticed that some areas of this research field applied different keywords (i.e. in the parasite-host literature compared to the evolutionary ecological and immunological literature) to their studies. Therefore, we conducted an additional search (setting the cut-off date to that of the first literature search), using a modified indexing (Additional file 1, Table S1). This second search generated 3452 research papers, which was reduced to 2692 after duplicates with the previous literature search had been eliminated (Fig. 1b).

In both literature searches, the majority of papers could not be dismissed based on title alone and were therefore subject to a more thorough scan. In brief, to qualify for inclusion in the meta-analysis, a study had to include an experiment in which some individuals were subjected to an immune challenge and others to a procedural control, and explore the associated effects on components of life-history or fitness (survival, reproductive output, development times, morphology) or on proximate immune traits (studies selected included the following immune parameters: phenoloxidase [PO], antimicrobial activity, Phytohemagglutinin-induced wing-swelling, antibody production and encapsulation response). Immune challenges that adopted a eukaryote immune agent were not included (e.g. challenge with fungi, parasitoids), nor were studies in which the host organism was an intermediate vector. Furthermore, we only included gonochoristic species that were obligately sexually reproducing, because one of our main priorities was to explore the role of sex in shaping immune responses. We focused only on studies that had conducted experiments on natural “outbred” populations (including “wild-type” laboratory maintained populations) of their associated study species, which were maintained and/or assayed under benign environmental or standard laboratory conditions (i.e. we leveraged data only from studies in which the authors claimed that populations were wild-type individuals in which each individual possessed a different genotype—thus, not including studies of clones or inbred lines—and in which individuals were not assayed under conditions of environmental stress). We did this to increase the generality of our results, since the results of studies leveraging inbred lines, selection lines, genetically modified lines or clones are more likely to be limited to the particular genotypes or conditions used [i.e. subject to G × E interactions] rather than generalisable to the species as a whole. Finally, studies containing additional treatment levels were excluded, unless we were able to extract enough data from the control group of such studies (e.g. if there were three diet treatments, we could use the data from the un-manipulated diet group assuming it contained a control and an immune challenge). For a full list of conditions for inclusion in our study, refer to Additional file 1, pp. 6–7, “Inclusion criteria for data extraction”.

### Data extraction and data management

One researcher extracted all the data (MN), but ambiguous cases were discussed between the two authors until a unanimous decision was reached. Each study had to include information on sample size, in association with raw means/proportions or numbers/odds ratios/hazard ratios, or statistics allowing the calculation of effect sizes and variance. Furthermore, we collected information on author names, title, publication year, journal, species, type of immune treatment, administration mode (i.e. dietary (N_ES_ *n* = 13), external exposure (*N*_ES_ = 20), implant (*N*_ES_ = 12) and injection (*N*_ES_ = 190), and whenever possible, information on sex, age at immune treatment and any additional information that could bear implications for data interpretation. When data was only presented in figure format, we extracted means and variances using the free online tool WebPlotDigitizer (https://automeris.io/WebPlotDigitizer/). Moreover, in the cases where the data was of generally high quality (hence, worth pursuing), but lacking in crucial information such as sample size or standard errors, we emailed the author(s) to request the missing information. In total, emails were sent out to 48 different research groups, generating a reply rate of 50% and a data return rate of 33%.

For the component of the data addressing survival, we used odds ratios (OR), because this enabled us to utilise more of the available data (i.e. the majority of the data was in the form of frequencies or proportions close to zero or one, which was not suitable for transformation to standardised means). All odds ratios were transformed to log odds ratios (ln OR) in the statistical analysis [92], but were back-transformed to OR in figures for easier interpretation. For our data, odds ratios should be interpreted as demonstrating no effect of immune challenge on survival when data was 1 (0 for ln OR), a decreased survival following immune challenge when values were less than 1 (< 0 for ln OR), and an increased survival when values were more than 1 (> 0 for ln OR).

For all other data, the response variable was transformed to Hedges’ *g*, which is an effect size that calculates the standardised mean differences between two groups—here, between immune challenged and control individuals—while also correcting for the innate upward bias associated with effect sizes of standardised mean differences, in particular when low-sample studies are involved [92, 93]. For our data, a positive Hedges’ *g* is interpreted as the effect being larger in the treatment group relative to the control group, whereas a negative Hedges’ *g* indicates the opposite (e.g. a positive Hedge’s *g* for a reproductive value means that immune-challenged individuals had a generally higher reproductive output relative to control-challenged individuals).

We adopted the default formulas used to calculate both OR and Hedges’ *g* [92, 93], and applied the formula from Nakagawa and Cuthill (2007) in the calculation of associated correction factors (*J*) [94]. In the few cases where proportions were presented instead of means, the most appropriate transformation for all our data points was logit [92], which were calculated using the online calculator made available by Campbell collaboration (https://www.campbellcollaboration.org/effect-size-calculato.html).

Throughout, means were considered statistically significant when the confidence intervals did not transect 0 for Hedge’s *g*, or 1 for odds ratios.

### Phylogenetic tree

Phylogenetic trees were created with the online NCBI Taxonomy Browser Common tree tool (https://www.ncbi.nlm.nih.gov/Taxonomy/CommonTree/wwwcmt.cgi), and imported in R, using the *ape* package. Because all trees contained one or more polytomies, they were modified in *Mesquite* [95]. In particular, the phylogenetic relationships between families in the infraorder *Passerida* were supplemented with information from the phylogenies presented in the interactive Tree of Life for *Passerida* (https://itol.embl.de/) [96], whereas the polytomies associated with holeometabola insects were resolved based on information presented in several different studies [97–100]. All the generated trees were topological (without branch length) and were therefore transformed to ultrametric trees using *compute.brlen* (applying Grafen’s computation) from the *ape* package. Final phylogenetic trees are presented in Additional file 1, Fig. S1, S5, S9, S17, and S21.

### Statistical analysis

All statistical analysis were run in RStudio, v. 1.1.383 [101], using the packages *metafor* [102] and *MCMCglmm* [103]. The focal analyses were conducted using the *metafor* package, as were model-specific heterogeneity statistics (*I*^*2*^ for total variance, variance due to study, phylogeny, and group identity [when relevant], as well as effect size specific variance, and phylogenetic heritability [*H*^2^]) [104]. As a rule of thumb, *I*^*2*^ values up to 25% are regarded as low, up to 50% as moderate and around 75% or more as high [56]. Moreover, estimates of conditional (i.e. fixed and random effects) and marginal (i.e. fixed effects) variance (*R*^2^) were assessed in *MCMCglmm*, based on the modified approach to estimate *R*^2^ in multilevel models [105]. The analyses using *MCMCglmm* were run across 650,000 iterations, applying a thinning of 50 and a burn in of 150,000, thus generating 10,000 samples of the chain. We used a parameter-expanded prior that was close to non-informative (*V* = 1, nu = 0.002 and *G* = *V* = 1, nu = 1, alpha.mu = 0, alpha. *V* = 1000) in all models, and all models were checked for autocorrelations and chain convergence using Gelman-Rubin statistics [106].

First, we ran a meta-analytic model for each trait, in which the intercept was the only fixed factor, and study identity, species identity, focal group identity (i.e. when multiple traits were measured from the same group of individuals, the group shared a code; not included in analyses of survival, where this was redundant because measurements were never repeated in the same group) and effect size identity (i.e. unique identifier for each data point) were fitted as random effects. In addition, we ran a second meta-analytic model for each trait, in which we accounted for phylogenetic signal by fitting a phylogenetic correlation matrix, constructed from the phylogenetic trees (Additional file 1, Figure S1, S5, S9, S17, and S21).

Second, moderators were fitted to both the phylogenetic and the non-phylogenetic model, for each response trait, to explore the contribution of each moderator, in isolation (univariate meta-regression model) and in full (meta-regression containing all moderators, and whenever power allowed it, the interactions between them). While we had extracted information for a number of potential moderators, there was only sufficient information in the full data set to meaningfully pursue two moderators in concert; “life-history stage” (because sex is difficult to determine in most sub-adult animals, sex-specific data at the time of injection was only available for juveniles in one study, and hence we combined data on sex and age into one composite moderator of three levels; juvenile, adult female and adult male) and “treatment agent” (whether or not the immune challenge treatment involved a living [i.e. replicating] or non-living [i.e. non-replicating agent, such as a heat-killed bacterium] challenge—see Additional file 1, Table S3 for more detail). Moreover, because of limited sample sizes, the interaction associated with these moderators was only tested in the analysis of survival and immune trait expression. Note that our assignment of age refers to the ontogenetic stage at which the individual was immune challenged (juvenile or adulthood), rather than the time of sampling, which generally varied across studies (i.e. traits were estimated at different ages or time intervals).

To rule out the possibility that any observed effects were driven simply by the type of immune system an animal possessed (acquired and innate systems of vertebrates versus innate systems only in invertebrates), we also conducted an additional test in which we explored the influence of these two major bilaterian metazoan groups on the effect sizes (for simplicity, referred to as “animal kingdom group”). Note that this test could only be applied to the non-phylogenetic models because of the collinearity of this taxonomic factor (vertebrates versus invertebrates) with phylogeny.

In addition to the analysis of the full data set, we also conducted two analyses on subsets of the data. First, we extracted data that only contained adult individuals to further probe the effect of mating status (mated or virgin) on host response to an immune challenge. Note that this data was limited to invertebrates, as there were not sufficient samples sizes to conduct a meaningful comparison across vertebrates. There was only sufficient data to conduct this sub-analysis on three of the five traits: survival, reproduction, and immunity. Also, because our data were not specifically aimed at extracting information on mating status relative to immune trait expression, these results should be interpreted with caution given their non-balanced nature across studies and species (e.g. we did not specifically extract studies comparing mated animals to non-mated animals from within the same experiment, nor always from within the same species). Second, we extracted the subset of data that investigated the effects of immune challenge specifically on immune trait expression, and probed whether any such effects were contingent on the class of immune trait measured (Additional file 1, Fig. S12a-b). Due to the limited sample sizes within each of these classes of immune trait, our comparisons ended up being limited to studies on invertebrates that had measured phenoloxidase (PO) and antimicrobial activity (Additional file 1, Fig. S13). The rationale to adopt a higher-resolution analysis of the effects on immune challenge on immune trait expression, however, was grounded on findings showing that the expression of such immune parameters can be positively correlated, neutral or negatively correlated with each other [70–74].

All models were fitted with a variance-covariance matrix controlling for interdependence between data points due to shared control groups (i.e. some studies applied different treatments but were compared to the same control group). The variance-covariance matrix was generated following the procedure adopted by Moatt and colleagues [107], which is based on equations presented in [108]. An example of the R code used for all analyses (with minor modification) can be found in Additional file 3.

### Publication bias

Assessment of publication bias was conducted using three methods: (1) visual assessment of funnel plots, (2) Egger’s regression to model residuals and (3) Trim-and-fill analysis on model residuals. The visual assessment was primarily conducted on model residuals plotted against precision. Egger’s regression statistically tests if there is a relationship between effect sizes and the study precision; because of interdependence in our data, we modelled meta-analytical and meta-regression residuals against precision values (1/sqrt V) [109]. We ran the trim-and-fill using both the estimator L_0_ and R_0_ [110]. Because the trim-and-fill analysis in *metafor* can only be done on fixed or random models (not on meta-regression), and given the interdependence in the data, we conducted trim-and-fill analysis on the residuals that were created in the *MCMCglmm* models [102, 109]. Likewise, Egger’s regression and final funnel plots were created from residuals generated in *MCMCglmm*.

## Supplementary information

**Additional file 1.** Data search and data tables (Tables S1-S3). Supplementary information on: - Survival (Table S4-S11, Figure S1-S4, R^2^ full model). Reproduction (Table S12-S16, Figures S5-S8, R^2^ full model). Immune trait expression (Table S17-S25, Figures S9-S116, R^2^ full model). Morphology (Table S26-S30, Figure S17–20, R^2^ full model). Development time (Table S31-S25, Figure S21-S23, R^2^ full model). Mating subset data, females only (Figure S24). - Detailed discussion on comparison with previous meta-analysis exploring proximate immune expression in animals (Discussion and associated References).

**Additional file 2.** Supplementary source data tables associated with the four main figures presented in the manuscript. Tables associated with Fig. 2 - meta-analyses (Table A2.S1_1 – A2.S5_1). Tables associated with Fig. 3 - meta-regressions (Table A2.S6 - A2.S9). Tables associated with Fig. 4 - meta-regression subsample for mating status (Table A2.S10 – A2.S12).

**Additional file 3.** R code example: this file includes the R code that was used for one of the traits. Note that with the exception of the survival analysis, which used lnOR as the response variable, the R code was largely identical for all traits; any modifications were related to the response variable or the inclusion/exclusion of a specific table or graph if there was a lack of data.

## Data Availability

The dataset supporting the conclusions of this article is available in the figshare repository [111]. Papers included in the meta-analysis are listed in Additional file 1: Table S2.

## References

[1] Janeway C, Travers P, Walport M, Shlomchik MJ (2001). Immunobiology: the immune system in health and disease.

[2] Sheldon B, Verhulst S (1996). Ecological immunology: costly parasite defences and trade-offs in evolutionary ecology. Trends Ecol Evol.

[3] Lochmiller R, Deerenberg C (2000). Trade-offs in evolutionary immunology: just what is the cost of immunity?. Oikos.

[4] Roff DA: Life history evolution. Sunderland: Sinauer; 2002.

[5] Stearns S (1989). Trade-offs in life-history evolution. Funct Ecol.

[6] Stearns S (1992). The evolution of life histories.

[7] Rolff J, Siva-Jothy M (2003). Invertebrate ecological immunology. Science.

[8] Lazzaro BP, Little TJ (2009). Immunity in a variable world. Philos Trans R Soc B-Biol Sci.

[9] Schmid-Hempel P (2005). Evolutionary ecology of insect immune defence. Annu Rev Entomol.

[10] Sadd BM, Schmid-Hempel P (2009). Principles of ecological immunology. Evol Appl.

[11] Schwenke RA, Lazzaro BP, Wolfner MF (2016). Reproduction–immunity trade-offs in insects. Annu Rev Entomol.

[12] McKean K, Yourth C, Lazzaro B, Clark A (2008). The evolutionary costs of immunological maintenance and deployment. BMC Evol Biol.

[13] Zerofsky M, Harel E, Silverman N, Tatar M (2005). Aging of the innate immune response in Drosophila melanogaster. Aging Cell.

[14] Masuzzo A, Manière G, Viallat-Lieutaud A, Avazeri É, Zugasti O, Grosjean Y, Kurz CL, Royet J (2019). Peptidoglycan-dependent NF-κB activation in a small subset of brain octopaminergic neurons controls female oviposition. eLife.

[15] Kurz CL, Charroux B, Chaduli D, Viallat-Lieutaud A, Royet J. Peptidoglycan sensing by octopaminergic neurons modulates Drosophila oviposition. Elife. 2017;6. pmid: 28264763. 10.7554/eLife.21937 PMC5365318 28264763

[16] Miyata S, Begun J, Troemel ER, Ausubel FM (2008). DAF-16-dependent suppression of immunity during reproduction in Caenorhabditis elegans. Genetics.

[17] Ahmed AM, Hurd H (2006). Immune stimulation and malaria infection impose reproductive costs in Anopheles gambiae via follicular apoptosis. Microb Infect.

[18] McNamara KB, van Lieshout E, Simmons LW (2014). Females suffer a reduction in the viability of stored sperm following an immune challenge. J Evol Biol.

[19] Roth O, Kurtz J (2008). The stimulation of immune defence accelerates development in the red flour beetle (Tribolium castaneum). J Evol Biol.

[20] Uller T, Isaksson C, Olsson M (2006). Immune challenge reduces reproductive output and growth in a lizard. Funct Ecol.

[21] Ilmonen P, Taarna T, Hasselquist D (2000). Experimentally activated immune defence in female pied flycatchers results in reduced breeding success. Proc Biol Sci.

[22] Bonneaud C, Mazuc J, Gonzalez G, Haussy C, Chastel O, Faivre B, Sorci G (2003). Assessing the cost of mounting an immune response. Am Nat.

[23] Schulenburg H, Kurtz J, Moret Y, Siva-Jothy MT (2009). Introduction. Ecological immunology. Philos Trans Royal Soc B: Biol Sci.

[24] Restif O, Amos W (2010). The evolution of sex-specific immune defences. Proc Royal Soc B-Bio Sci.

[25] Grindstaff JL, Brodie ED, Ketterson ED (2003). Immune function across generations: integrating mechanism and evolutionary process in maternal antibody transmission. Proc R Soc Lond B Biol Sci.

[26] Hasselquist D, Nilsson J-Å (2012). Physiological mechanisms mediating costs of immune responses: what can we learn from studies of birds?. Anim Behav.

[27] Klein SL, Flanagan KL (2016). Sex differences in immune responses. Nat Rev Immunol.

[28] Nunn CL, Lindenfors P, Pursall ER, Rolff J (2009). On sexual dimorphism in immune function. Philos Trans R Soc Lond Ser B Biol Sci.

[29] Cook MB, McGlynn KA, Devesa SS, Freedman ND, Anderson WF (2011). Sex disparities in Cancer mortality and survival. Cancer Epidemiol Biomark Prev.

[30] Kelly CD, Stoehr AM, Nunn C, Smyth KN, Prokop ZM (2018). Sexual dimorphism in immunity across animals: a meta-analysis. Ecol Lett.

[31] Andersson M (1994). Sexual selection.

[32] Bateman AJ (1948). Intra-sexual selection in *Drosophila*. Heredity (Edinb).

[33] Rolff J (2002). Bateman's principle and immunity. Proc R Soc Lond B Biol Sci.

[34] Zuk M (1990). Reproductive strategies and disease susceptibility: an evolutionary viewpoint. Parasitol Today.

[35] McKean K, Nunney L (2005). Bateman's principle and immunity: phenotypically plastic reproductive strategies predict changes in immunological sex differences. Evolution.

[36] Penn DJ, Smith KR (2007). Differential fitness costs of reproduction between the sexes. Proc Natl Acad Sci.

[37] Bolund E, Lummaa V, Smith KR, Hanson HA, Maklakov AA (2016). Reduced costs of reproduction in females mediate a shift from a male-biased to a female-biased lifespan in humans. Sci Rep.

[38] Kirkwood TB, Holliday R (1979). The evolution of ageing and longevity. Proc Royal Soc London Series B Biol Scie.

[39] Kirkwood TB (1977). Evolution of ageing. Nature.

[40] Lawniczak MKN, Barnes AI, Linklater JR, Boone JM, Wigby S, Chapman T (2007). Mating and immunity in invertebrates. Trends Ecol Evol.

[41] Simon AK, Hollander GA, McMichael A: Evolution of the immune system in humans from infancy to old age. Proc R Soc B Biol Sci 2015, 282(1821):20143085. 10.1098/rspb.2014.3085 PMC4707740 26702035

[42] Grindstaff JL (2008). Maternal antibodies reduce costs of an immune response during development. J Exp Biol.

[43] Lemaitre B, Hoffmann J (2007). The host defense of *Drosophila melanogaster*. Annu Rev Immunol.

[44] Meister M, Lagueux M (2003). Drosophila blood cells. Cell Microbiol.

[45] Crozatier M, Meister M (2007). Drosophila haematopoiesis. Cell Microbiol.

[46] Gardiner EM, Strand MR (2000). Hematopoiesis in larval Pseudoplusia includens and Spodoptera frugiperda. Arch Insect Biochem Physiol.

[47] Nardi JB, Pilas B, Ujhelyi E, Garsha K, Kanost MR (2003). Hematopoietic organs of Manduca sexta and hemocyte lineages. Dev Genes Evol.

[48] Hillyer JF (2016). Insect immunology and hematopoiesis. Dev Comp Immunol.

[49] Ribet D, Cossart P (2015). How bacterial pathogens colonize their hosts and invade deeper tissues. Microb Infect.

[50] Bhavsar AP, Guttman JA, Finlay BB (2007). Manipulation of host-cell pathways by bacterial pathogens. Nature.

[51] Agudelo-Romero P, Carbonell P, Perez-Amador MA, Elena SF (2008). Virus adaptation by manipulation of host's gene expression. PLoS One.

[52] Paschos K, Allday MJ (2010). Epigenetic reprogramming of host genes in viral and microbial pathogenesis. Trends Microbiol.

[53] Vojtech LN, Sanders GE, Conway C, Ostland V, Hansen JD (2009). Host immune response and acute disease in a zebrafish model of Francisella pathogenesis. Infect Immun.

[54] Sridhar S, Brokstad KA, Cox RJ (2015). Influenza vaccination strategies: comparing inactivated and live attenuated influenza vaccines. Vaccines.

[55] Hariharan IK, Wake DB, Wake MH (2015). Indeterminate growth: could it represent the ancestral condition?. Cold Spring Harb Perspect Biol.

[56] Higgins JPT, Thompson SG, Deeks JJ, Altman DG (2003). Measuring inconsistency in meta-analyses. BMJ.

[57] Nakagawa S, Noble DWA, Senior AM, Lagisz M (2017). Meta-evaluation of meta-analysis: ten appraisal questions for biologists. BMC Biol.

[58] Lim JN, Senior AM, Nakagawa S (2014). Heterogeneity in individual quality and reproductive trade-offs within species. Evolution.

[59] Martin LB, Weil ZM, Nelson RJ (2008). Seasonal changes in vertebrate immune activity: mediation by physiological trade-offs. Philos Trans Royal Soc B: Biol Sci.

[60] Ardia DR, Gantz JE, Schneider BC, Strebel S (2012). Costs of immunity in insects: an induced immune response increases metabolic rate and decreases antimicrobial activity. Funct Ecol.

[61] Schmid-Hempel P (2003). Variation in immune defence as a question of evolutionary ecology. Proc R Soc B Biol Sci.

[62] Stoehr AM, Kokko H (2006). Sexual dimorphism in immunocompetence: what does life-history theory predict?. Behav Ecol.

[63] Råberg L, Grahn M, Hasselquist D, Svensson E (1998). On the adaptive significance of stress-induced immunosuppression, vol. 265.

[64] Hanssen SA, Hasselquist D, Folstad I, Erikstad KE (2004). Costs of immunity: immune responsiveness reduces survival in a vertebrate. Proc R Soc Lond B Biol Sci.

[65] Sadd BM, Siva-Jothy MT (2006). Self-harm caused by an insect's innate immunity. Proc R Soc B Biol Sci.

[66] Tatar M, Carey JR (1995). Nutrition mediates reproductive trade-offs with age-specific mortality in the beetle Callosobruchus Maculatus. Ecology.

[67] Flatt T (2011). Survival costs of reproduction in Drosophila. Exp Gerontol.

[68] Zuk M, Stoehr A (2002). Immune defense and host life history. Am Nat.

[69] Barthel A, Staudacher H, Schmaltz A, Heckel DG, Groot AT (2015). Sex-specific consequences of an induced immune response on reproduction in a moth. BMC Evol Biol.

[70] Adamo SA (2004). How should behavioural ecologists interpret measurements of immunity?. Anim Behav.

[71] Cotter S, Kruuk L, Wilson K (2004). Costs of resistance: genetic correlations and potential trade-offs in an insect immune system. J Evol Biol.

[72] Elrod-Erickson M, Mishra S, Schneider D (2000). Interactions between the cellular and humoral immune responses in *Drosophila*. Curr Biol.

[73] Graham AL, Shuker DM, Pollitt LC, Auld S, Wilson AJ, Little TJ (2011). Fitness consequences of immune responses: strengthening the empirical framework for ecoimmunology. Funct Ecol.

[74] Nehme NT, Quintin J, Cho JH, Lee J, Lafarge M-C, Kocks C, Ferrandon D (2011). Relative roles of the cellular and humoral responses in the *Drosophila* host defense against three gram-positive bacterial infections. PLoS One.

[75] Adamo SA (2012). The strings of the puppet master: how parasites change host behavior. Host manipulation by parasites.

[76] Maitra A, Dill KA (2015). Bacterial growth laws reflect the evolutionary importance of energy efficiency. Proc Natl Acad Sci U S A.

[77] Blomberg SP, Garland T, Ives AR (2003). Testing for phylogenetic signal in comparative data: behavioral traits are more labile. Evolution.

[78] Tamara M, Sébastien L, Bruno B, Stéphane D, Thibaut J, Katja S, Wilfried T (2012). How to measure and test phylogenetic signal. Methods Ecol Evol.

[79] Boggs CL (2009). Understanding insect life histories and senescence through a resource allocation lens. Funct Ecol.

[80] Mousseau TA, Dingle H (1991). Maternal effects in insect life histories. Annu Rev Entomol.

[81] Mousseau TA, Uller T, Wapstra E, Badyaev AV (2009). Evolution of maternal effects: past and present. Philos Trans Royal Soc B: Biol Sci.

[82] Little TJ, O'Connor B, Colegrave N, Watt K, Read AF (2003). Maternal transfer of strain-specific immunity in an invertebrate. Curr Biol.

[83] Moret Y (2006). Trans-generational immune priming’: specific enhancement of the antimicrobial immune response in the mealworm beetle, *Tenebrio molitor*. Proc Royal Soc B-Biol Sci.

[84] Zanchi C, Troussard JP, Moreau J, Moret Y (2012). Relationship between maternal transfer of immunity and mother fecundity in an insect. Proc Royal Soc B-Biol Sci.

[85] Arnqvist G, Rowe L (2005). Sexual conflict.

[86] Chapman T, Arnqvist G, Bangham J, Rowe L (2003). Sexual conflict. Trends Ecol Evol.

[87] McKean K, Nunney L (2008). Sexual selection and immune function in *Drosophila melanogaster*. Evolution.

[88] Rolff J, Armitage SAO, Coltman DW (2005). Genetic constraints and sexual dimorphism in immune defense. Evolution.

[89] Siva-Jothy M, Moret Y, Rolff J (2005). Insect immunity: an evolutionary ecology perspective. Adv Insect Physiol.

[90] Zuk M, Mckean K (1996). Sex differences in parasite infections: patterns and processes. Int J Parasitol.

[91] Moher D, Liberati A, Tetzlaff J, Altman DG (2009). Preferred reporting items for systematic reviews and meta-analyses: the PRISMA statement. PLoS Med.

[92] Lipsey MW, Wilson DB (2001). Practical meta-analysis.

[93] Borenstein M, Hedges LV, Higgins JPT, Rothstein HR: Effect Sizes Based on Means. In: Introduction to Meta-Analysis. Chichester: Wiley 2009: 21–32.

[94] Nakagawa S, Cuthill IC (2007). Effect size, confidence interval and statistical significance: a practical guide for biologists. Biol Rev.

[95] Maddison WP, Maddison DR (2017). Mesquite: a modular system for evolutionary analysis. Version 3.2 (build 801).

[96] Letunic I, Bork P (2007). Interactive tree of life (iTOL): an online tool for phylogenetic tree display and annotation. Bioinformatics.

[97] Peters RS, Meusemann K, Petersen M, Mayer C, Wilbrandt J, Ziesmann T, Donath A, Kjer KM, Aspöck U, Aspöck H (2014). The evolutionary history of holometabolous insects inferred from transcriptome-based phylogeny and comprehensive morphological data. BMC Evol Biol.

[98] Misof B, Liu S, Meusemann K, Peters RS, Donath A, Mayer C, Frandsen PB, Ware J, Flouri T, Beutel RG (2014). Phylogenomics resolves the timing and pattern of insect evolution. Science.

[99] Danforth BN, Brady SG, Sipes SD, Pearson A (2004). Single-copy nuclear genes recover cretaceous-age divergences in bees. Syst Biol.

[100] Song F, Li H, Jiang P, Zhou X, Liu J, Sun C, Vogler AP, Cai W (2016). Capturing the phylogeny of Holometabola with mitochondrial genome data and Bayesian site-heterogeneous mixture models. Genome Biol Evol.

[101] R Development Core Team (2012). R: A language and environment for statistical computing. R Foundation for statistical computing.

[102] Viechtbauer W. Conducting Meta-Analyses in R with the metafor Package. Journal of Statistical software. 2010;36(3):48.

[103] Hadfield JD. MCMC Methods for multi-response generalized linear mixed models: the MCMCglmm R Package. Journal of Statistical software. 2010;33(2):22.

[104] Nakagawa S, Lagisz M, Hector KL, Spencer HG (2012). Comparative and meta-analytic insights into life extension via dietary restriction. Aging Cell.

[105] Nakagawa S, Schielzeth H (2013). A general and simple method for obtaining R2 from generalized linear mixed-effects models. Methods Ecol Evol.

[106] Gelman A, Rubin DB (1992). Inference from iterative simulation using multiple sequences. Stat Sci.

[107] Moatt JP, Nakagawa S, Lagisz M, Walling CA (2016). The effect of dietary restriction on reproduction: a meta-analytic perspective. BMC Evol Biol.

[108] Gleser LJ, Olkin I (2009). Stochastically dependent effect sizes. The handbook of research synthesis and meta-analysis.

[109] Nakagawa S, Santos EA (2012). Methodological issues and advances in biological meta-analysis. Evol Ecol.

[110] Duval S, Tweedie R (2000). Trim and fill: a simple funnel-plot–based method of testing and adjusting for publication bias in meta-analysis. Biometrics.

[111] Nystrand M, Dowling DK: Effects of immun challenge on expression of life-history and immune trait expression in sexually reproducing metazoans - a meta-analysis. figshare 2020, Data set:doi: 10.26180/5f44732b3dabf. 10.1186/s12915-020-00856-7 PMC7541220 33028304

