# Supplementary material for: Effects of immune challenge on expression of life-history and immune trait expression in sexually reproducing metazoans—a meta-analysis
Source: BMC Biol. 2020 Oct 7;18:135. doi: 10.1186/s12915-020-00856-7 (PMC7541220; doi:10.1186/s12915-020-00856-7)
Supplement: Supplementary file 1 — Additional file 1. Data search and data tables (Tables S1-S3). Supplementary information on: - Survival (Table S4-S11, Figure S1-S4, R2 full model). Reproduction (Table S12-S16, Figures S5-S8, R2 full model). Immune trait expression (Table S17-S25, Figures S9-S116, R2 full model). Morphology (Table S26-S30, Figure S17–20, R2 full model). Development time (Table S31-S25, Figure S21-S23, R2 full model). Mating subset data, females only (Figure S24). - Detailed discussion on comparison with previous meta-analysis exploring proximate immune expression in animals (Discussion and associated References). [file 12915_2020_856_MOESM1_ESM.pdf]

Data search and Data table 1-3

- Table S1. Data search string & Inclusion criteria for searches
- Table S2. List of included studies
- Table S3. Number of effect sizes for each type of treatment agent

Survival

- Table S4. Effect of treatment agent and life-history stage, and the interaction between the two, on survival (non-phylogenetic model)
- Table S5. Effect of animal kingdom group (vertebrates vs. invertebrates) on survival
- Table S6. Effect of all moderators, including which animal kingdom group (vertebrates vs. invertebrates) the host belonged to, on survival (non-phylogenetic model).
- Table S7. Effect of all moderators and the interaction between treatment agent and life-history status, including which animal kingdom group (vertebrates vs. invertebrates) the host belonged to, on survival (non-phylogenetic model).

TABLE S8-11: ANALYSES IN WHICH PHYLOGENY HAS BEEN ACCOUNTED FOR:

- Table S8 (associated with Figure 2a). Effect of “life-history status” on survival (phylogenetic model).
- Table S9 (associated with Figure 2a). Effect of “treatment agent” (replicating agent [bacteria and virus] vs. non-replicating agent [heat-killed bacteria or virus, LPS, implant]) on survival (phylogenetic model).
- Table S10 (associated with Figure 3a). Effect of all moderators on survival (phylogenetic model).
- Table S11. Effect of treatment agent and life-history stage, and the interaction between the two, on survival (*phylogenetic model*).
- Figure S1. Phylogenetic tree of study species included in the analysis of survival, modified in Mesquite.
- Figure S2. Forest plot of the non-phylogenetic meta-analytic model (lnOR are backtransformed to original scale for increased comprehension).
- Figure S3. Forest plot showing the effect of major animal kingdom group on organismal survival, following an immune challenge.
- Figure S4. Upper left-hand pane shows the funnel plot from the original meta-analytic (nonphylogenetic) model generated in metaphor (y-axis shows SE).
- R^2 results from full model

Reproduction

- Table S12. Effect of animal kingdom group (vertebrates vs. invertebrates) on reproduction
- Table S13. Effect of all moderators on reproduction (non-phylogenetic model), but where the effect of animal kingdom (vertebrate vs. invertebrate) has been added to the model.

TABLE S14-16: ANALYSES IN WHICH PHYLOGENY HAS BEEN ACCOUNTED FOR:

- Table S14 (associated with Figure 2b). Effect of “life-history status” on reproduction (phylogenetic model).
- Table S15 (associated with Figure 2b). Effect of “treatment agent” (replicating agent [bacteria and virus] vs. non-replicating agent [heat-killed bacteria or virus, LPS, implant]) on reproduction (phylogenetic model).
- Table S16 (associated with Figure 3b). Effect of all moderators on reproduction (phylogenetic model).
- Figure S5. Phylogenetic tree of study species included in the analysis of reproductive success, modified in Mesquite.
- Figure S6. Forest plot of the non-phylogenetic meta-analytic model.
- Figure S7. Forest plot showing the effect of major animal kingdom group on organismal reproduction, following an immune challenge.
- Figure S8. Upper left-hand pane shows the funnel plot from the original meta-analytic (nonphylogenetic) model generated in metaphor (y-axis shows SE).
- R^2 results from full model

Immune trait expression

- Table S17. Effect of all moderators and the interaction between life-history status and treatment agent on immune trait expression (non-phylogenetic model).
- Table S18. Effect of animal kingdom group (vertebrates or invertebrates) on immune trait expression.
- Table S19. Effect of all moderators on immune trait expression (*non-phylogenetic model*), but where the effect of animal kingdom group (invertebrates or vertebrates) has been added to the model.
- Table S20 Effect of all moderators, including the interaction between life-history and treatment agent, on immune trait expression (*non-phylogenetic model*), but where the effect of animal kingdom group (invertebrates or vertebrates) has been added to the model.

<u>TABLE S21-24: ANALYSES IN WHICH PHYLOGENY HAS BEEN ACCOUNTED FOR:</u>

- Table S21 (associated with Figure 2c). Effect of “life-history status” on immune trait expression (*phylogenetic model*).

- Table S22 (associated with Figure 2c). Effect of “treatment agent” (replicating agent [bacteria and virus] vs. non-replicating agent [heat-killed bacteria or virus, LPS, implant]) on immune trait expression (*phylogenetic model*).

- Table S23 (associated with Figure 3c). Effect of all moderators (main effects only) on immune trait expression (*phylogenetic model*).

- Table S24. Effect of all moderators and the interaction between life-history status and treatment agent on immune trait expression (*phylogenetic model*).

- Figure S9. Phylogenetic tree of study specie included in the analysis of immune trait expression, modified in Mesquite.

- Figure S10. Forest plot of the non-phylogenetic meta-analytic model.

- Figure S11. Forest plot showing the effect of major animal kingdom group on immune trait expression, following an immune challenge.

SUBSAMPLE ANALYSIS EXPLORING THE EFFECT OF IMMUN ASSAY VARIABLE:

- Table S25. Effect of “immune trait” (PO or antimicrobial activity) and life-history stage on immune trait expression following an immune challenge (*non-phylogenetic model*).

- Figure S12a. Forest plot of effect sizes for all immune traits.

- Figure S12b. Forest plot of contrast between effect sizes for all immune traits (reference level is antimicrobial activity).

- Figure S13. Forest plot of the subset of immune trait expression data (only including PO and antimicrobial activity).

- Figure S14. Forest plot showing the effect of life-history status on the subset of immune trait expression data (only including PO and antimicrobial activity).

- Figure S15. Full meta-regression forest plot of immune trait expression subset data containing antimicrobial activity and PO (reference level = males, PO, intercept = -0.096 ± SE 0.480, CI = -1.036 – 0.844).

Forest plots full data set:

- Figure S16. Upper left-hand pane shows the funnel plot from the original meta-analytic (non-phylogenetic) model generated in metaphor (SE displayed on y-axis).

- R^2 results full model

Morphology

- Table S26. Effect of major animal kingdom group (vertebrate vs. invertebrate) on morphology.

- Table S27. Effect of all moderators on morphology (non-phylogenetic model), but where the effect of animal kingdom group (vertebrate vs. invertebrate) has been added to the model.

TABLE S28-30: ANALYSES IN WHICH PHYLOGENY HAS BEEN ACCOUNTED FOR:

- Table S28 (associated with Figure 2d). Effect of “life-history status” on morphology (*phylogenetic model*).

- Table S29 (associated with Figure 2d). Effect of “treatment agent” (replicating agent [bacteria and virus] vs. non-replicating agent [heat-killed bacteria or virus, LPS, implant]) on morphology (*phylogenetic model*).

- Table S30 (associated with Figure 3d). Effect of all moderators on morphology (*phylogenetic model*).

- Figure S17. Phylogenetic tree of study species included in the analysis of morphological traits, modified in Mesquite.

- Figure S18. Forest plot of the non-phylogenetic meta-analytic model.

- Figure S19. Forest plot showing the effect of major animal kingdom group on morphology, following an immune challenge.

- Figure S20. Upper left-hand pane shows the funnel plot from the original meta-analytic (non-phylogenetic) model generated in metaphor (y-axis shows SE).

- R^2 results full model

Development time

TABLE S1: ANALYSES IN WHICH PHYLOGENY HAS BEEN ACCOUNTED FOR:

- Table S21. Effect of “treatment agent” (replicating agent [bacteria and virus] vs. non-replicating agent [heat-killed bacteria or virus, LPS, implant]) on development times (*phylogenetic model*).
- Figure S22. Phylogenetic tree of study species included in the analysis of development times, modified in Mesquite.
- Figure S23. Forest plot of the non-phylogenetic meta-analytic model.
- R^2 results full model

Mating subset data, females only

- Figure S24. Mating subset only including females.

- Table S2. Effect of animal kingdom group (vertebrates vs. invertebrates) on survival

Detailed discussion on comparison with previous meta-analysis exploring proximate immune expression in animals

- Discussion & References

ADDITIONAL FILE 1: DATA SEARCH, INCLUSION CRITERIA AND DATA EXTRACTION; LIST OF INCLUDED STUDIES; LIST OF TREATMENT AGENTS

Data search

The institutional subscription for the Web of science – all databases include Web of Science core collection, Current contents, Data citation index, Medline, SciELO, Alerts, Journal Citation Reports.

Table S1. Meta-analysis search string

a) **SEARCH STRING OF MAIN (FIRST) LITERATURE SEARCH**, November 26, 2016.
TS=("immune function*" OR "immune system" OR "immune systems" OR "immune respons*" OR "immunity" OR "immunocompetence" OR "immune challeng*" OR "immunolog*")<br>*DocType=All document types; Language=All languages;*<br><br>*AND*
TS=("evolution" OR "evolutionary" OR "ecology" OR "ecological" OR "ecoimmunolog*" OR "ecological immunolog*" OR "eco immunolog*" OR "genetics" OR "genetical" OR "behaviour" OR "behavior" OR "behavioural" OR "behavioral")<br>*DocType=All document types; Language=All languages;*<br><br>*AND*
TS=("survival" OR "mortality" OR "reproduction" OR "fitness" OR "fertility" OR "reproductive success" OR "longevity" OR "fecundity" OR "life span" OR "lifespan" OR "aging" OR "ageing" OR "senescence")
b) **SEARCH STRINGS OF SECOND LITERATURE SEARCH,** November 27, 2016 cut-off (actual search conducted on February 28th, 2017)
TS=(host fitness)<br><br>*AND*
TS=(virus OR viral OR bacteria OR bacterium OR bacterial OR fungi OR fungus OR pathogen OR pathogenic OR LPS OR Lipopolysaccharide)<br><br>*AND*
TS=("survival" OR "mortality" OR "reproduction" OR "fitness" OR "fertility" OR "reproductive success" OR "longevity" OR "fecundity" OR "life span" OR "lifespan" OR "aging" OR "ageing" OR "senescence")<br>*DocType=All document types; Language=All languages;*

Inclusion criteria for data extraction

1. They study had to investigate one more key life-history traits, i.e. *reproduction* (e.g. egg number, number of eclosed offspring, clutch size, offspring-to-adult viability), *survival, or development.* We also included studies that specifically addressed *morphological traits* that are generally tightly linked to reproductive success and/or survival (i.e. body size, body mass, body length or body growth).

2. We only used sexually reproducing, gonadochristic host organisms. The strain should be “wild-type”; hence, knock-out strains, inbred strains, clonal strains, selection lines or in any other way “genetically modified” strains were excluded.

3. A requirement for inclusion was that the host organism had been actively treated with an immune challenge (thus not including studies that only measured natural, back-ground levels of immunity) complying with one or more of the following categories:

   a. Bacterial challenge (alive or inactivated)
   b. Virus (alive or inactivated)
   c. Other established immune elicitor (e.g. LPS, Peptidoglycans, PHA, Nylon filament insertion).

   However, we did *not* include studies that used Eukaryotes (e.g. fungi, parasitoids, protozoans) to immune challenge the host organism, nor did we use studies that only injured (i.e. pricked) the animal to induce and immune response.

   d. Studies that explored highly specilised and unique systems were not included. Specifically, obligate endosymbionts, such as Woolbachia and Rikosettia, that commonly influence, alter or even determine the outcome of organismal immune function, were excluded, as were studies conducted on vector organisms.
   e. The study had to include a proper control, in which control animal had been actively challenged – i.e. procedural control (i.e. injected with a buffer, or treated with media similar to that introduced to the immune treated animals but without containing the immune trigger). Because we wanted to be able to separate the effects of the actual immune challenge from that of mechanical injury alone, we did not include controls in which the animal had only been handled.

4. Only traits that were a direct measure of organismal immune response were included (e.g. haemocyte number, haemocyte activity, PO, proPO, lytic activity/antimicrobial activity, white blood count [neutrophils, monocytes, basophils, heterophils, or NK cells]). Hence,

studies exploring traits that may be associated with immunity – and hence, use them as an indirect indicator of immune response (such as metabolism or haematocrit levels) - were not included.

5. Studies that contained additional treatment levels (for example, temperature or diet treatments) were only included if the treatment control-group ("untreated") category could be fully separated out from the treated groups.

6. In cases where highly correlated data had been collected in same individual, and where no averages or overall estimates were available, we used the "final data point" to avoid pseudo-replication (for example, developmental data sometimes contained information on both the time to pupation and the time to adult eclosion in the same individual, in which case we only used the time to adult eclosion data point). However, whenever such measurements were conducted in different individuals, all data was used.

Table S2. List of included studies

Animal	Study details	Survival	Reproduction	Immune trait expression	Morphology	Development times	Sex	Age	Animal class	Animal phylum	Author	Article
Acheta domesticus	lab	survival	egg number (adults only)	NA	body size	NA	female unknown	adult juvenile	Insecta	Arthropoda	Bascunan-Garcia, A. P.; Lara, C.; Cordoba-Aguilar, A. (2010)	Immune investment impairs growth, female reproduction and survival in the house cricket, Acheta domesticus
Ambystoma tigrinum	lab	proportion dead alive at the end of experiment	NA	NA	NA	NA	unknown	juvenile	Amphibia	Chordata	Kerby, J. L.; Hart, A. J.; Storfer, A. (2011)	Combined Effects of Virus, Pesticide, and Predator Cue on the Larval Tiger Salamander (Ambystoma tigrinum)
Anopheles gambiae	lab	NA	egg number	antimicrobial activtiy	NA	NA	female	adult	Insecta	Arthropoda	Ahmed, A. M.; Baggott, S. L.; Maingon, R.; Hurd, H. (2003)	The costs of mounting an immune response are reflected in the reproductive fitness of the mosquito Anopheles gambiae
Anopheles stephensi	lab	survival	egg number	NA	NA	NA	female	adult	Insecta	Arthropoda	Ohm, J. R.; Teeple, J.; Nelson, W. A.; Thomas, M. B.; Read, A. F.; Cator, L. J. (2016)	Fitness consequences of altered feeding behavior in immune-challenged mosquitoes
Apis mellifera	wild-caught lab	survival	NA	NA	NA	NA	female	adult	Insecta	Arthropoda	Riessberger-Galle, U.; Lopez, J. H.; Schuehly, W.; Crockett, S.; Krainer, S.; Crailsheim, K. (2015)	Immune responses of honeybees and their fitness costs as compared to bumblebees
Bombus terrestris	wild	mean colony survival length	reproductive fitness	NA	NA	NA	female	adult	Insecta	Arthropoda	Cisarovsky, G.; Koch, H.; Schmid-Hempel, P. (2012)	A field study on the influence of food and immune priming on a

												bumblebee-gut parasite system
Bombus terrestris	lab	NA	number of workers producedm number of queens produced	NA	fat body of workers	NA	female	adult	Insecta	Arthropoda	Moret, Y.; Schmid-Hempel, P. (2004)	Social life-history response to individual immune challenge of workers of Bombus terrestris L.: a possible new cooperative phenomenon
Clupea pallasii	lab	survival	NA	NA	NA	NA	unknown	juvenile	Actinopterygii	Chordata	Hershberger, P K.; Gregg, J.; Pacheco, C.; Winton, J.; Richard, J.; Traxler, G. (2007)	Larval Pacific herring, Clupea pallasii (Valenciennes), are highly susceptible to viral haemorrhagic septicaemia and survivors are partially protected after their metamorphosis to juveniles
Ctenophorus fordi	wild	NA	clutch size, egg size	NA	NA	NA	female	adult	Reptilia	Chordata	Uller, T.; Isaksson, C.; Olsson, M. (2006)	Immune challenge reduces reproductive output and growth in a lizard
Culex pipiens	lab	NA	number of eggs, number of larvae	NA	NA	NA	female	adult	Insecta	Arthropoda	Ciota, A. T.; Ehrbar, D. J.; Matacchiero, A.C.; Van Slyke, G.A.; Kramer, L. D. (2013)	The evolution of virulence of West Nile virus in a mosquito vector: implications for arbovirus adaptation and evolution
Culex pipiens	lab	NA	NA	PO	body mass	development time to 4th metamorphosis	female male unknown* (*juvenile body fat & juvenile PO)	juvenile	Insecta	Arthropoda	Op de Beeck, L.; Janssens, L.; Stoks, R. (2016)	Synthetic predator cues impair immune function and make the biological pesticide Bti more lethal for vector mosquitoes

Culiseta melanura	lab	NA	number of larvae	NA	NA	NA	female	adult	Insecta	Arthropoda	Scott, T. W.; Lorenz, L.H. (1998)	Reduction of Culiseta melanura fitness by eastern equine encephalomyelitis virus
Delichon urbicum	wild	NA	nestling number (prop)	NA	NA	NA	female	adult	Aves	Chordata	Marzal, A.; Reviriego, M.; de Lope, F.; Moller, A. P. (2007)	Fitness costs of an immune response in the house martin (Delichon urbica)
Drosophila melanogaster	lab	survival	NA	NA	body mass dry mass	NA	male	adult	Insecta	Arthropoda	Arnold, P. A.; Johnson, K. N.; White, C. R. (2013)	Physiological and metabolic consequences of viral infection in Drosophila melanogaster
Drosophila melanogaster	lab	survival	NA	NA	NA	NA	male	adult	Insecta	Arthropoda	Corby-Harris, V.; Promislow, D.E.L. (2008)	Host ecology shapes geographical variation for resistance to bacterial infection in Drosophila melanogaster
Drosophila melanogaster	lab	NA	offspring number	NA	NA	NA	female male	adult	Insecta	Arthropoda	Khan,I.; Prasad, N.G. (2013)	Male Drosophila melanogaster show adaptive mating bias in response to female infection status
Drosophila melanogaster	lab	survival	number of eggs, egg-to-adult viability, number of eclosed offspring	NA	NA	NA	female	adult	Insecta	Arthropoda	Kutzer, M.A.M.; Armitage, S.A.O. (2016)	The effect of diet and time after bacterial infection on fecundity, resistance, and tolerance in Drosophila melanogaster

Drosophila melanogaster	lab	survival	number of offspring	NA	NA	NA	female male	adult	Insecta	Arthropoda	Lazzaro, B. P.; Flores, H. A.; Lorigan, J. G.; Yourth, C. P. (2008)	Genotype-by-environment interactions and adaptation to local temperature affect immunity and fecundity in Drosophila melanogaster
Drosophila melanogaster	lab	longevity (mean)	fecundity (number of eggs), viability egg-to-adults), prop. reproductive success (paternity)	antimicrobial activity (mean)	NA	NA	female male	adult	Insecta	Arthropoda	Nystrand, M.; Dowling, D. K. (2014)	Dose-dependent effects of an immune challenge at both ultimate and proximate levels in Drosophila melanogaster
Dryophytes chrysoscelis, Pseudacris feriarum, Rana clamitans, Rana sylvatica	lab	survival	NA	NA	NA	NA	unknown	juvenile	Amphibia	Chordata	Haislip, N.A.; Hoverman, J.T.; Miller D.L.; Gray, M.J. (2012)	Natural stressors and disease risk: does the threat of predation increase amphibian susceptibility to ranavirus?
Enallagma cyathigerum	lab	survival	NA	haemocytes	NA	growth rate	unknown	juvenile	Insecta	Arthropoda	Janssens, L.; Stoks.R. (2014)	Non-pathogenic aquatic bacteria activate the immune system and increase predation risk in damselfly larvae
Gryllus campestris	lab	survival	NA	NA	body mass change 3 daysafter treatment (g)	NA	male	adult	Insecta	Arthropoda	Jacot, A.; Scheuber, H.; Brinkhof, M.W.G. (2004)	Costs of an induced immune response on sexual display and longevity in field crickets
Gryllus texensis	lab	lifespan (mean days)	hatching success (mean number) and fertilizaion success (mean prop.)	NA	weigh at last day injections (kg at day 16)	NA	female	adult	Insecta	Arthropoda	Shoemaker, K. L.; Adamo, S.A. (2007)	Adult female crickets, Gryllus texensis, maintain reproductive output after repeated immune challenges

Gryllus texensis	lab	survival	NA	NA	NA	na	female	adult	Insecta	Arthropoda	Shoemaker, K.L.; Parsons, N.M.; Adamo, S.A. (2006)	Egg-laying behaviour following infection in the cricket Gryllus texensis
Gryllus texensis	lab	NA	NA	PO	NA	NA	female	adult	Insecta	Arthropoda	Stahlschmidt, Z.R.; Acker, M.; Kovalko, I.; Adamo, S.A. (2015)	The double-edged sword of immune defence and damage control: do food availability and immune challenge alter the balance?
Gryllus texensis	lab	NA	ovipositioning (eggs/day)	PO	NA	NA	female	adult	Insecta	Arthropoda	Stahlschmidt, Z. R.; Rollinson, N.; Acker, M.; Adamo, S.A. (2013)	Are all eggs created equal? Food availability and the fitness trade-off between reproduction and immunity
Helicoverpa armigera	lab	adult longevity (prop)	NA	antimicrobial activity, PO (mean)	NA	larval duiration, pupal duration ((mean)	female* male unknown (females only for reprod., unknown for immun. and dev.)	juvenile	Insecta	Arthropoda	McNamara, K.B.; van Lieshout, E.; Jones, T.M.; Simmons, L.W. (2013)	Age-dependent trade-offs between immunity and male, but not female, reproduction
Heliothis virescens	lab	survival	egg number	NA	NA	NA	female	adult	Insecta	Arthropoda	Staudacher, H.; Menken, S.B.J.; Groot, A.T. (2015)	Effects of immune challenge on the oviposition strategy of anoctuid moth
Hemideina crassidens	lab	NA	egg length, proportion egg layers, egg mass	NA	body condition (fat load)	NA	female	adult	Insecta	Arthropoda	Kelly, C.D. (2011)	Reproductive and physiological costs of repeated immune challenges in female Wellington tree weta (Orthoptera: Anostostomatidae)

Hetaerina americana	wild	survival	NA	PO	NA	NA	male	adult	Insecta	Arthropoda	Contreras-Garduno, J.; Lanz-Mendoza, H.; Cordoba-Aguilar, A. (2007)	The expression of a sexually selected trait correlates with different immune defense components and survival. in males of the American rubyspot
Hetaerina titia	lab	survival	NA	NA	NA	NA	male	adult	Insecta	Arthropoda	Gonzalez-Tokman, D.M.; Cordoba-Aguilar, A. (2010)	Survival after experimental manipulation in the territorial damselfly Hetaerina titia (Odonata: Calopterygidae): more ornamented males are not more pathogen resistant
Hirundo rustica	wild	NA	NA	NA	body mass across day 2 and 3 post-infection combined	NA	unknown	juvenile	Aves	Chordata	Romano, A.; Rubolini, D.; Caprioli, M.; Boncoraglio, G.; Ambrosini, R.; Saino, N. (2011)	Sex-Related Effects of an Immune Challenge on Growth and Begging Behavior of Barn Swallow Nestlings
Mus musculus	lab	NA	embryos viable early, number ovulated ovas	NA	NA	NA	male	adult	Mammalia	Chordata	Moshkin, M. P.; Kondratyuk, E. Y.; Litvinova, E. A.; Gerlinskaya, L. A. (2010)	The activation of specific immunity in male mice stimulates fertility of their breeding partners: The phenomenon of Lot's daughters
Passer domesticus	lab	NA	number of fledglings (mean)	NA	NA	NA	female	adult	Aves	Chordata	Bonneaud, C.; Mazuc, J.; Gonzalez, G.; Haussy, C.; Chastel, O.; Faivre, B.; Sorci, G. (2003)	Assessing the cost of mounting an immune response

Passer domesticus	lab	NA	NA	NA	body mass change, tarsus length	NA	male	adult	Aves	Chordata	Moreno-Rueda,G. (2010)	Experimental test of a trade-off between moult and immune response in house sparrows Passer domesticus
Passer domesticus	lab	NA	NA	NA	body mass change	NA	female	adult	Aves	Chordata	Moreno-Rueda,G. (2011)	Trade-off between immune response and body mass in wintering house sparrows (Passer domesticus)
Phodopus sungorus	lab	NA	offspring number, litter mass, prop. Successful pregnancies	NA	NA	NA	female		Mammalia	Chordata	French, S.S.; Chester, E.M.; Demas, G.E. (2013)	Maternal immune activation affects litter success, size and neuroendocrine responses related to behavior in adult offspring
Phodopus sungorus	lab	NA	number of pups	bacterial clearance	body mass	NA	female	adult	Mammalia	Chordata	French, S.S.; Chester, E.M.; Demas, G.E. (2016)	Timing of Maternal Immunization Affects Immunological and Behavioral Outcomes of Adult Offspring in Siberian Hamsters (Phodopus sungorus)
Plocepasser mahali	wild	NA	NA	NA	NA	NA	female male	adult	Mammalia	Chordata	Cram, D.L.; Blount, J.D.; York, J.E.; Young, A.J. (2015)	Immune Response in a Wild Bird Is Predicted by Oxidative Status, but Does Not Cause Oxidative Stress
Plocepasser mahali	wild	NA	NA	wing swelling (mean)	body mass (24 h post challenge, mean)	NA	male	adult	Mammalia	Chordata	York, J E.; Radford, A.N.; Groothuis, T.G.; Young, A.J. (2016)	Dominant male song performance reflects current immune state in a coopera-tively breeding songbird

Rattus norvegicus	lab	NA	pup number	NA	NA	NA	female	adult	Mammalia	Chordata	Paris, J.J.; Brunton, P.J.; Russell, J.A.; Frye, C.A. (2011)	Immune stress in late pregnant rats decreases length of gestation and fecundity, and alters later cognitive and affective behaviour of surviving pre-adolescent offspring
Rattus norvegicus	lab	NA	litter size	NA	body weight	time until maturation	female	juvenile	Mammalia	Chordata	Sominsky, L.; Meehan, C.L.; Walker, A.K.; Bobrovskaya, L.; McLaughlin, E A.; Hodgson, D.M. (2012)	Neonatal immune challenge alters reproductive development in the female rat
Stomoxys calcitrans	lab	NA	egg number	NA	NA	NA	female	adult	Insecta	Arthropoda	Geden, C.; Garcia-Maruniak, A.; Lietze, V.U.; Maruniak, J.; Boucias, D.G. (2011)	Impact of House Fly Salivary Gland Hypertrophy Virus (MdSGHV) on a Hetero-logous Host, Stomoxys calcitrans
Streptopelia decaocto	wild	survival	NA	NA	body mass at fledgling (g)	NA	unknown	juvenile	Aves	Chordata	Eraud, C.; Jacquet, A.; Faivre, B. (2009)	Survival cost of an early immune soliciting in nature
Teleogryllus oceanicus	lab	NA	sperm viability	encapsulation response, antimicrobial activity	NA	NA	male	juvenile	Insecta	Arthropoda	Simmons, L.W. (2014)	Resource allocation trade-off between sperm quality and immunity in the field cricket, Teleogryllus oceanicus
Tenebrio molitor	lab	survival	NA	NA	NA	NA	male	adult	Insecta	Arthropoda	Krams, I.; Daukste, J.; Kivleniece, I.; Krama, T.; Rantala, M.J.; Ramey, G.; Sausa, L. (2011)	Female choice reveals terminal investment in male mealworm beetles, Tenebrio molitor, after a repeated activation of the immune system

Tenebrio molitor	lab	NA	NA	PO	NA	NA	male	adult	Insecta	Arthropoda	Sadd, B.; Holman, L.; Armitage, H.; Lock, F.; Marland, R.; Siva-Jothy, M.T. (2006)	Modulation of sexual signalling by immune challenged male mealworm beetles (Tenebrio molitor, L.): evidence for terminal investment and dishonesty
Tenebrio molitor	lab	NA	number of larvae (mean)	antimicrobial activity (mean)	NA	NA	female male	adult	Insecta	Arthropoda	Zanchi, C.; Troussard, J.P.; Martinaud, G. ; Moreau, J.; Moret, Y. (2011)	Differential expression and costs between maternally and paternally derived immune priming for offspring in an insect
Tribolium castaneum	lab	NA	NA	antimicrobial activity	NA	NA	female male	adult	Insecta	Arthropoda	Khan, I.; Prakash, A.; Agashe, D. (2016)	Immunosenescence and the ability to survive bacterial infection in the red flour beetle Tribolium castaneum
Tribolium castaneum	lab	NA	NA	NA	body size (larvae)	proportion developed into adults	unknown	juvenile	Insecta	Arthropoda	Milutinovic, B.; Fritzlar, S.; Kurtz, J. (2014)	Increased Survival in the Red Flour Beetle after Oral Priming with Bacteria-Conditioned Media
Tribolium castaneum	lab	NA	offspring number	NA	NA	adult eclosion (time to)	female	juvenile	Insecta	Arthropoda	Roth, O.; Kurtz, J. (2008)	The stimulation of immune defence accelerates development in the red flour beetle (Tribolium castaneum)
Troglodytes aedon	wild	NA	clutch size, fledged offspring, replacement clutch	antibody proudction	NA	NA	female	adult	Aves	Chordata	Bowers, E.K.; Smith, R.A.; Hodges, C.J.; Zimmerman, L.M.; Thompson, C.F.; Sakaluk, S.K. (2012)	Sex-biased terminal investment in offspring induced by maternal immune challenge in the house wren (Troglodytes aedon)

Zootermopsis angusticollis	lab	survival	NA	NA	NA	NA	unknown	juvenile	Insecta	Arthropoda	Calleri, D.V.; Reid, E.M.; Rosengaus, R.B.; Vargo, E.L.; Traniello, J.F.A. (2006)	Inbreeding and disease resistance in a social insect: effects of heterozygosity on immune-competence in the termite Zootermopsis angusticollis

											adolescent offspring
Rattus norvegicus	lab	NA	litter size	NA	body weight	time until maturation	female	Mammalia	Chordata	Sominsky, L.; Meehan, C.L.; Walker, A.K.; Bobrovskaya, L.; McLaughlin, E A.; Hodgson, D.M. (2012)	Neonatal immune challenge alters reproductive development in the female rat
Stomoxys calcitrans	lab	NA	egg number	NA	NA	NA	female	Insecta	Arthropoda	Geden, C.; Garcia-Maruniak, A.; Lietze, V.U.; Maruniak, J.; Boucias, D.G. (2011)	Impact of House Fly Salivary Gland Hypertrophy Virus (MdSGHV) on a Hetero-logous Host, Stomoxys calcitrans
Streptopelia decaocto	wild	survival	NA	NA	body mass at fledgling (g)	NA	unknown	Aves	Chordata	Eraud, C.; Jacquet, A.; Faivre, B. (2009)	Survival cost of an early immune soliciting in nature
Teleogryllus oceanicus	lab	NA	sperm viability	encapsulation response, antimicrobial activity	NA	NA	male	Insecta	Arthropoda	Simmons, L.W. (2014)	Resource allocation trade-off between sperm quality and immunity

											in the field cricket, Teleogryllus oceanicus
Tenebrio molitor	lab	survival	NA	NA	NA	NA	male	Insecta	Arthropoda	Krams, I.; Daukste, J.; Kivleniece, I.; Krama, T.; Rantala, M.J.; Ramey, G.; Sausa, L. (2011)	Female choice reveals terminal investment in male mealworm beetles, Tenebrio molitor, after a repeated activation of the immune system
Tenebrio molitor	lab	NA	NA	PO	NA	NA	male	Insecta	Arthropoda	Sadd, B.; Holman, L.; Armitage, H.; Lock, F.; Marland, R.; Siva-Jothy, M.T. (2006)	Modulation of sexual signalling by immune challenged male mealworm beetles (Tenebrio molitor, L.): evidence for terminal investment and dishonesty
Tenebrio molitor	lab	NA	number of larvae (mean)	antimicrobial activity (mean)	NA	NA	both	Insecta	Arthropoda	Zanchi, C.; Troussard, J.P.; Martinaud, G. ; Moreau, J.; Moret, Y. (2011)	Differential expression and costs between maternally and paternally derived immune

											priming for offspring in an insect
Tribolium castaneum	lab	NA	NA	antimicrobial activity	NA	NA	both	Insecta	Arthropoda	Khan, I.; Prakash, A.; Agashe, D. (2016)	Immunosenescence and the ability to survive bacterial infection in the red flour beetle Tribolium castaneum
Tribolium castaneum	lab	NA	NA	NA	body size (larvae)	proportion developed into adults	juvenile	Insecta	Arthropoda	Milutinovic, B.; Fritzlar, S.; Kurtz, J. (2014)	Increased Survival in the Red Flour Beetle after Oral Priming with Bacteria-Conditioned Media
Tribolium castaneum	lab	NA	offspring number	NA	NA	adult eclosion (time to)	female	Insecta	Arthropoda	Roth, O.; Kurtz, J. (2008)	The stimulation of immune defence accelerates development in the red flour beetle (Tribolium castaneum)

Troglodytes aedon	wild	NA	clutch size, fledged offspring, replacement clutch	antibody proudction	NA	NA	female	Aves	Chordata	Bowers, E.K.; Smith, R.A.; Hodges, C.J.; Zimmerman, L.M.; Thompson, C.F.; Sakaluk, S.K. (2012)	Sex-biased terminal investment in offspring induced by maternal immune challenge in the house wren (Troglodytes aedon)
Zootermopsis angusticollis	lab	survival	NA	NA	NA	NA	unknown	Insecta	Arthropoda	Calleri, D.V.; Reid, E.M.; Rosengaus, R.B.; Vargo, E.L.; Traniello, J.F.A. (2006)	Inbreeding and disease resistance in a social insect: effects of heterozygosity on immune-competence in the termite Zootermopsis angusticollis

Table S3. Number of effect sizes for each type of treatment agent (replicating or non-replicating agent), also providing details of how the treatment agent was administered (Mode of infection) and the sort of immune challenge used.

	Effect sizes (N)		
**Mode of infection**	**non-replicating**	**replicating**	**Grand Total**
**diet**		**13**	**13**
bacteria - alive		8	8
virus - alive		5	5
**external**		**20**	**20**
bacteria - alive		13	13
virus - alive		7	7
**implant**	**12**		**12**
nylon	12		12
**injection**	**151**	**39**	**190**
antigen	12		12
bacteria - alive		35	35
bacteria - dead	37		37
LPS	98		98
PHA	4		4
virus - alive		4	4
**Grand Total**	**163**	**72**	**235**

ADDITIONAL FILE 1 - SURVIVAL

Table S4. Effect of treatment agent and life-history stage, and the interaction between the two, on survival (***non-phylogenetic model***). Effect sizes used for statistical tests were logged OR (back-transformed in brackets. Reference level in full model is adult males that were challenged with a non-replicating agent. AIC-values were generated from a maximum likelihood model.

**MODERATORS** *(AIC = 162.16)*	**ES lnOR (OR)**	**SE**	**CI LOWER (OR)**	**CI UPPER (OR)**
Life-history status				
*Adult females**	*-0.268 (0.765)*	*0.122*	*-0.507 (0.602)*	*-0.030 (0.971)*
Juveniles	0.361(1.435)	0.193	-1.976 (0.138)	2.698 (14.856)
Treatement agent				
*Replicating agent**	*-1.519 (0.219)*	*0.535*	*-2.568 (0.077)*	*-0.471 (0.624)*
Life-hist. stat. x Treat. agent				
Replic. agent × adult female	1.081 (2.947)	0.583	-0.063 (0.939)	2.224 (9.245)
Replic. agent × juvenile	0.178 (1.195)	1.456	-2.676 (0.069)	3.031(20.720)

QM(df = 5) = 14.3502, p-val = 0.0135; QM interaction only (df = 2) = 3.4325, p-val = 0.1797

Table S5. Effect of animal kingdom group (vertebrates *vs*. invertebrates) on survival. Effect sizes used for statistical tests were logged OR (lnOR). Sample size of effect sizes for each group of the moderator is given in a separate column, followed by the associated number of studies in brackets.

**MODERATORS**	**ES (lnOR)**	**N_{ES} (studies)**	**SE**	**CI LOWER**	**CI UPPER**
Vertebrates	-2.162 (0.115)	8 (4)	0.923	-3.970 (0.019)	-0.354 (0.702)
Invertebrates	-1.094 (0.335)	43 (19)	0.386	-1.851 (0.157)	-0.338 (0.713)
Contrast (Vert – Invert)	-1.068 (0.344)	--	1.000	-3.028 (0.048)	0.892 (2.441)

QM(df = 1) = 1.1403, p-val = 0.2856

Table S6. Effect of all moderators, including which animal kingdom group (vertebrates *vs*. invertebrates) the host belonged to, on survival (***non-phylogenetic model***). Effect sizes used for statistical tests were logged OR, also displaying back-transformed lnOR in brackets. Reference level for the full model is adult, invertebrate males that were challenged with a non-replicating agent. AIC-values are generated from a ML model.

**MODERATORS** *(AIC = 161.91)*	**ES lnOR (OR)**	**SE**	**CI LOWER (OR)**	**CI UPPER (OR)**
Life-history status				
Adult females	-0.223 (0.800)	0.119	-0.457 (0.633)	0.011 (1.011)
Juveniles	1.174 (3.234)	0.009	-0.803 (0.448)	3.151 (23.347)
Treatment agent				
*Replicating agent**	*-1.332 (0.264)*	*0.489*	*-2.291 (0.101)*	*-0.372 (0.689)*
Animal kingdom group				
Vertebrates	-1.620 (0.198)	1.260	-4.090 (0.017)	0.850 (1.340)

QM(df = 4) = 12.6850, p-val = 0.0129

Table S7. Effect of all moderators and the interaction between treatment agent and life-history status, including which animal kingdom group (vertebrates *vs*. invertebrates) the host belonged to, on survival (***non-phylogenetic model***). Effect sizes used for statistical tests were logged OR, also displaying back-transformed lnOR in brackets. Reference level for the full model is adult, invertebrate males that were challenged with a non-replicating agent. AIC-values were generated from a ML model.

**MODERATORS** *(AIC = 162.17)*	**ES lnOR (OR)**	**SE**	**CI LOWER (OR)**	**CI UPPER (OR)**
Life-history status				
*Adult females**	*-0.267 (0.765)*	*0.122*	*-0.506 (0.603)*	*-0.029 (0.972)*
Juveniles	1.090 (2.974)	1.298	-1.455 (0.233)	3.635 (37.895)
Treatment agent				
*Replicating agent**	*-1.532 (0.216)*	*0.532*	*-2.574 (0.076)*	*-0.489 (0.613)*
Animal kingdom group				
Vertebrates	-1.645 (0.193)	1.263	-4.120 (0.016)	0.831 (1.295)
Treat. agent x life-hist. stat.				
Replic. Agent × Adult female	1.085 (2.958)	0.582	-0.056 (0.945)	2.226 (9.259)
Replic. Agent × juvenile	0.405 (1.499)	1.444	-2.424 (0.089)	3.234 (25.381)

QM(df = 6) = 16.2494, p-val = 0.0125; interaction term only: QM(df = 2) = 3.4988, p-val = 0.1739

TABLE S8-11: ANALYSES IN WHICH PHYLOGENY HAS BEEN ACCOUNTED FOR

Table S8 (associated with Figure 2a). Effect of “life-history status” on survival (***phylogenetic model***). Since the majority of studies only assigned sex to adult individuals, sex and age were combined into one moderator consisting of three levels: adult females, adult males, and juveniles. Effect sizes used for statistical tests were lnOR. However, back-transformed values (OR) are given in brackets. Sample size of effect sizes for each group of the moderator is given in a separate column, followed by the associated number of studies in brackets.

**MODERATORS** *(AIC = 169.21)*	**ES lnOR (OR)**	N_{ES} **(studies)**	**SE**	**CI LOWER(OR)**	**CI UPPER (OR)**
Adult female*	-1.334 (0.263)	21 (10)	0.443	-2.202 (0.111)	-0.466 (0.627)
Adult male*	-1.106 (0.331)	16 (8)	0.440	-1.977 (0.138)	-0.237 (0.789)
Juvenile	-1.272 (0.280)	14 (7)	0.705	-2.654 (0.070)	0.110 (1.116)
Contrast (female-male)	-0.227 (0.797)	-	0.120	-0.462 (0.630)	0.007 (1.007)
Contrast (juv-male)	-0.165 (0.848)	-	0.833	-1.798 (0.166)	1.468 (4.339)
Contrast (juv-female)	0.062 (1.064)	-	0.833	-1.570 (0.208)	1.694 (5.440)

QM(df = 2) = 3.6177, p-val = 0.1638

Table S9 (associated with Figure 2a). Effect of “treatment agent” (replicating agent [bacteria and virus] vs. non-replicating agent [heat-killed bacteria or virus, LPS, implant]) on survival (***phylogenetic model***). Effect sizes used for statistical tests were logged OR (lnOR). Sample size of effect sizes for each group of the moderator is given in a separate column, followed by the associated number of studies in brackets.

**MODERATORS** *(AIC = 162.84)*	**ES lnOR (OR)**	N_{ES} **(studies)**	**SE**	**CI LOWER**	**CI UPPER**
Replic. agent*	-2.012 (0.134)	23 (11)	0.428	-2.850 (0.058)	-0.173 (0.309)
Non-replic. agent	-0.700 (0.497)	28 (13)	0.381	-1.447 (0.235)	0.047 (1.048)
Contrast (Replic. –Non-replic.)*	-1.312 (0.269)	--	0.484	-2.260 (0.104)	-0.363 (0.696)

QM(df = 1) = 7.3414, p-val = 0.0067

Table S10 (associated with Figure 3a). Effect of all moderators on survival (***phylogenetic model***). Effect sizes used for statistical tests were logged OR (lnOR). Reference level in full model is adult mated males that were challenged with a non-replicating agent. AIC-values are generated from a ML model.

**MODERATORS** *(AIC = 163.75)*	**ES lnOR (OR)**	**SE**	**CI LOWER (OR)**	**CI UPPER (OR)**
Life-history status				
Adult females	-0.225 (0.799)	0.119	-0.459 (0.632)	0.010 (1.010)
Juveniles	0.317(1.373)	0.770	-1.192 (0.304)	1.825 (6.205)
Treatment agent				
*Replicating agent**	*-1.354 (0.258)*	*0.492*	*-2.318 (0.099)*	*-0.389 (0.678)*

QM(df = 3) = 10.8987, p-val = 0.0123

Table S11. Effect of treatment agent and life-history stage, and the interaction between the two, on survival (***phylogenetic model***). Displayed effect sizes, that were used for statistical tests are lnOR, but backtransformed values are shown brackets. Reference level in full model is adult males that were challenged with a non-replicating agent. AIC-values were generated from a maximum likelihood model.

**MODERATORS** *(AIC = 164.16)*	**ES lnOR (OR)**	**SE**	**CI LOWER (OR)**	**CI UPPER (OR)**
Life-history status				
*Adult females**	*-0.268 (0.765)*	*0.122*	*-0.507 (0.602)*	*-0.030 (0.971)*
Juveniles	0.361(1.435)	0.193	-1.976 (0.138)	2.698 (14.856)
Treatement agent				
*Replicating agent**	*-1.519 (0.219)*	*0.535*	*-2.568 (0.077)*	*-0.471 (0.624)*
Life-hist. stat. x Treat. agent				
Replic. agent × adult female	1.081 (2.947)	0.583	-0.063 (0.939)	2.224 (9.245)
Replic. agent × juvenile	0.178 (1.195)	1.456	-2.676 (0.069)	3.031(20.720)

QM(df = 5) = 13.6693, p-val = 0.0179; interaction term only: QM (df = 2) = 3.4885, p-val = 0.1748.

FIGURES

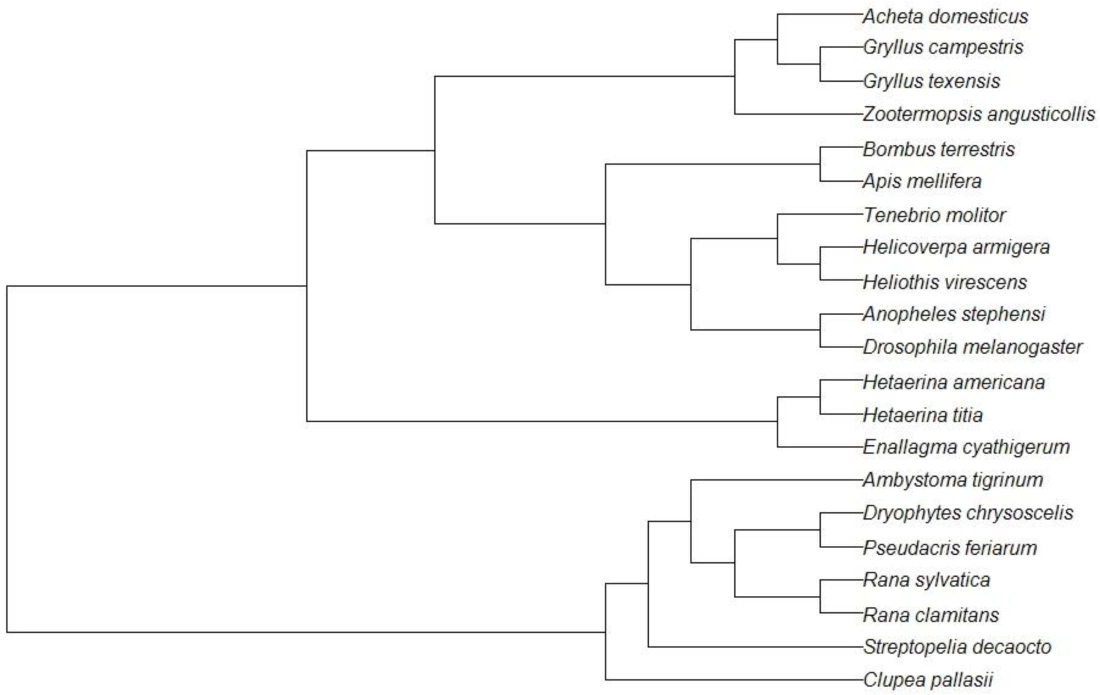

Figure S1. Phylogenetic tree of study species included in the analysis of survival, modified in Mesquite.

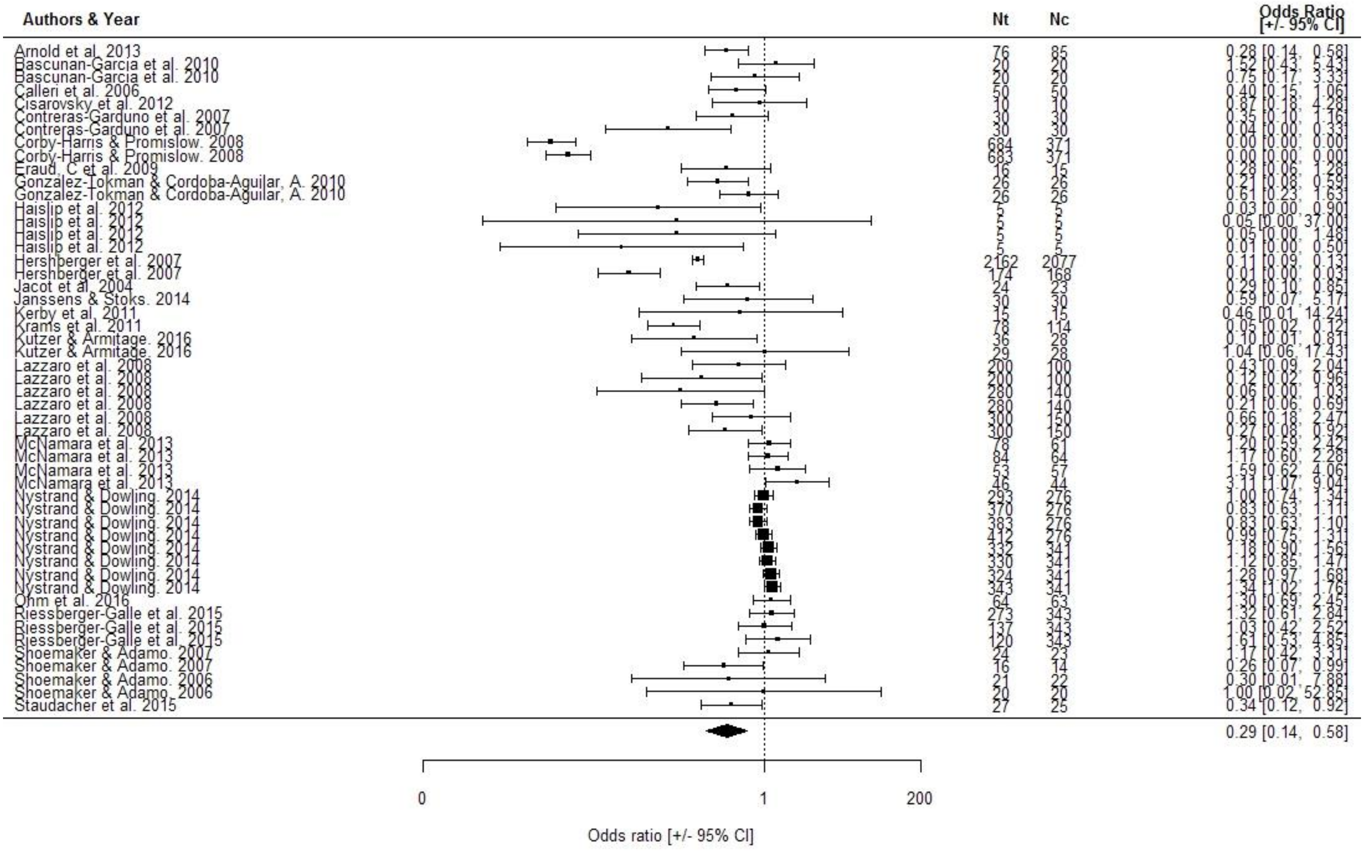

Figure S2. Forest plot of the non-phylogenetic meta-analytic model (lnOR are backtransformed to original scale for increased comprehension). Where values are < 1, the control group has higher survival than the treated group, whereas values > 1 indicated the opposite.

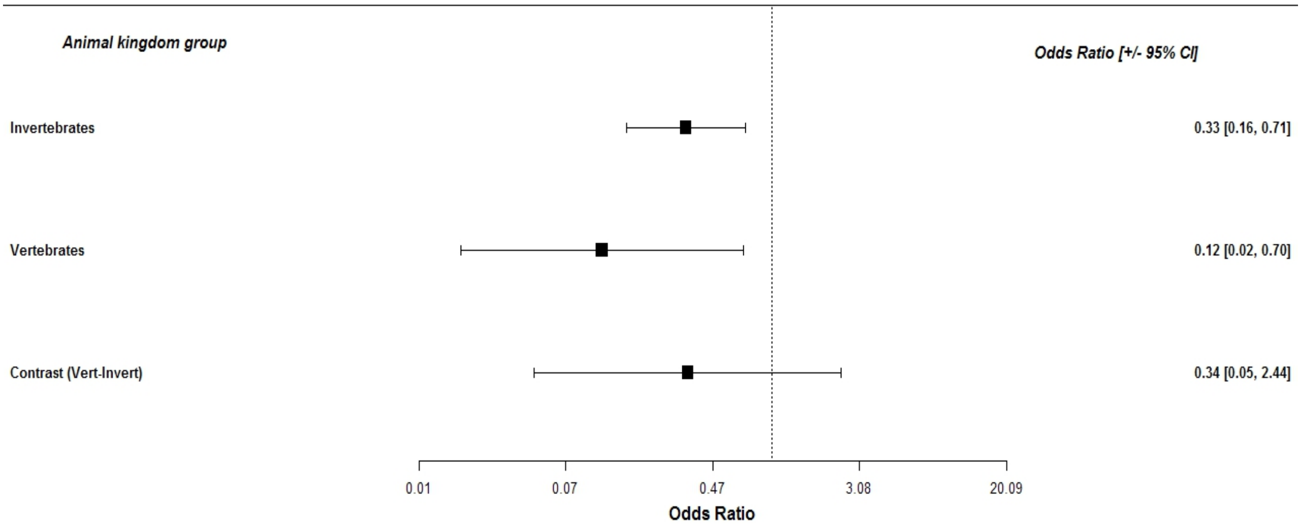

Figure S3. Forest plot showing the effect of major animal kingdom group on organismal survival, following an immune challenge.

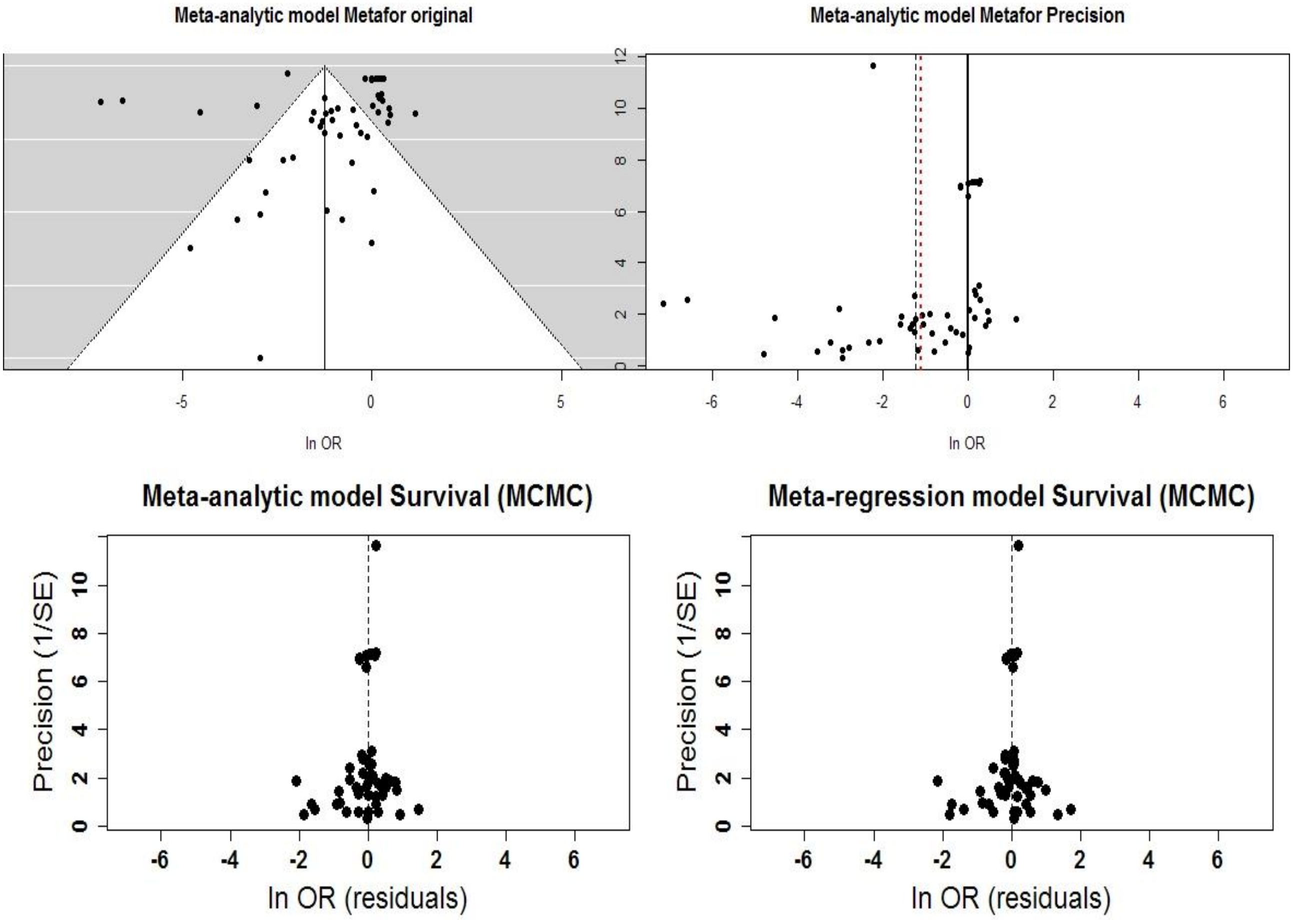

Figure S4. Upper left-hand panes shows the funnel plot from the original meta-analytic (non-phylogenetic) model generated in metaphor (y-axis shows SE). Upper right hand panel shows the same data, but where the y-axis show precision (1/SE). For comparison, we also show the mean from the metafor model (red hatched line) and the posterior mean from the corresponding MCMCglmm model (solid black line). Lower left-hand panel illustrates the funnel plot from the meta-analytic model generated in MCMCglmm, but in which the x-axis display residuals and the y-axis precision (1/SE). Finally, the right-hand panel shows the corresponding model for the meta-regression data (main effects). Zero effect sizes (i.e. no effect of treatment) are plotted as hatched black lines intersecting zero [0] in all the modified funnel plots.

R^2 RESULTS FULL MODEL

Marginal: 11.09 %

Conditional: 99.32 %

Hence, random = 88.23 %

ADDITIONAL FILE 1 - REPRODUCTION

Table S12. Effect of major animal kingdom group (vertebrate *vs.* invertebrate) on reproduction. Effect sizes used for statistical tests were Hedges' *g*. Sample size of effect sizes for each group of the moderator is given in a separate column, followed by the associated number of studies in brackets.

**MODERATORS**	**ES (H*g*)**	**N_{ES} (studies)**	**SE**	**CI LOWER**	**CI UPPER**
Vertebrates	-0.088	8 (4)	0.155	-0.391	0.216
Invertebrates	-0.147	44 (21)	0.084	-0.311	0.017
Contrast (Vert-Invert)	0.059	--	0.176	-0.285	0.404

QM (df = 1) = 0.1142, p-val = 0.7355

Table S13. Effect of all moderators on reproduction (*non-phylogenetic model*), but where the effect of animal kingdom (vertebrate *vs.* invertebrate) has been added to the model. Effect sizes used for statistical tests were Hedges' *g*. The reference level in the full model reflects adult females that were challenged with a non-replicating agent. AIC-values are generated from a ML model.

**MODERATORS** *(AIC = 122.94)*	**ES (H*g*)**	**SE**	**CI LOWER**	**CI UPPER**
Life-history status				
*Adult females**	*-0.261*	*0.123*	*-0.502*	*-0.020*
Juveniles	-0.001	0.272	-0.533	0.531
Treatment agent				
Replicating agent	-0.183	0.187	-0.549	0.183
Animal kingdom group				
Vertebrates	0.024	0.196	-0.361	0.408

QM (df = 4) = 7.3317, p-val = 0.1194

TABLE S14-16: ANALYSES IN WHICH PHYLOGENY HAS BEEN ACCOUNTED FOR

Table S14 (associated with Figure 2b). Effect of "life-history status" on reproduction (***phylogenetic model***). Since the majority of studies only assigned sex to adult individuals, sex and age were combined into one moderator consisting of three levels: adult females, adult males, and juveniles. Effect sizes used for statistical tests were Hedges' *g*. Sample size of effect sizes for each group of the moderator is given in a separate column, followed by the associated number of studies in brackets.

**MODERATORS** *(AIC = 122.18)*	**ES (H*g*)**	**N_{ES} (studies)**	**SE**	**CI LOWER**	**CI UPPER**
*Adult female**	*-0.199*	*58(24)*	*0.080*	*-0.356*	*-0.042*
Adult male	0.068	10 (4)	0.133	-0.193	0.329
Juvenile	0.098	12 (4)	0.208	-0.310	0.506
Contrast (female-male)*	*-0.267*	-	*0.122*	*-0.506*	*-0.028*
Contrast (juv-male)	0.030	-	0.247	-0.454	0.514
Contrast (juv-female)	0.297	-	0.223	-0.140	0.734

QM(df = 2) = 6.2256, p-val = 0.0445

Table S15 (associated with Figure 2b). Effect of "treatment agent" (replicating agent [bacteria and virus] vs. non-replicating agent [heat-killed bacteria or virus, LPS, implant]) on reproduction (***phylogenetic model***). Effect sizes used for statistical tests were Hedges' *g*. Sample size of effect sizes for each group of the moderator is given in a separate column, followed by the associated number of studies in brackets.

**MODERATORS** *(AIC = 124.24)*	**ES (H*g*)**	**N_{ES} (studies)**	**SE**	**CI LOWER**	**CI UPPER**
*Replic. agent**	*-0.300*	*18 (7)*	*0.137*	*-0.568*	*-0.032*
Non-replic. agent	-0.067	62 (22)	0.085	-0.234	0.101
Contrast (Replic. –Non-replic.)	-0.234	--	0.161	-0.550	0.082

QF (df = 1) = 2.1044, p-val = 0.1469

Table S16 (associated with Figure 3b). Effect of all moderators on reproduction (***phylogenetic model***). Effect sizes used for statistical tests were Hedges' *g*. The reference level in the full model reflects adult females that were challenged with a non-replicating agent. AIC-values are generated from a ML model.

**MODERATORS** *(AIC = 122.94)*	**ES (H*g*)**	**SE**	**CI LOWER**	**CI UPPER**
Life-history status				
*Adult females**	*-0.261*	*0.122*	*-0.500*	*-0.021*
Juveniles	-0.017	0.256	-0.519	0.485
Treatment agent				
Replicating agent	-0.187	0.173	-0.526	0.153

QM (df = 3) = 7.3770, p-val = 0.0608

FIGURES

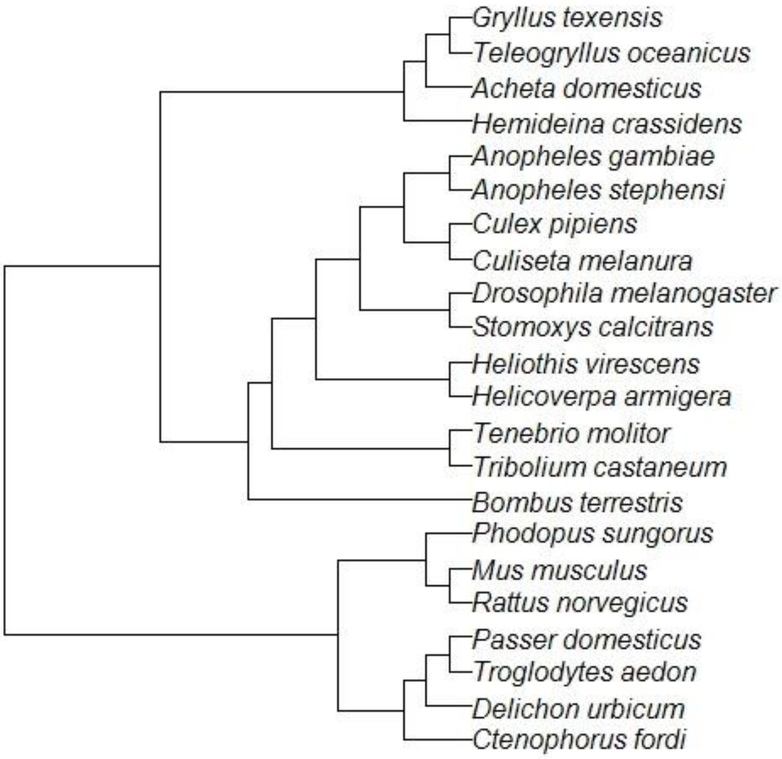

Figure S5. Phylogenetic tree of study species included in the analysis for reproductive success, modified in Mesquite.

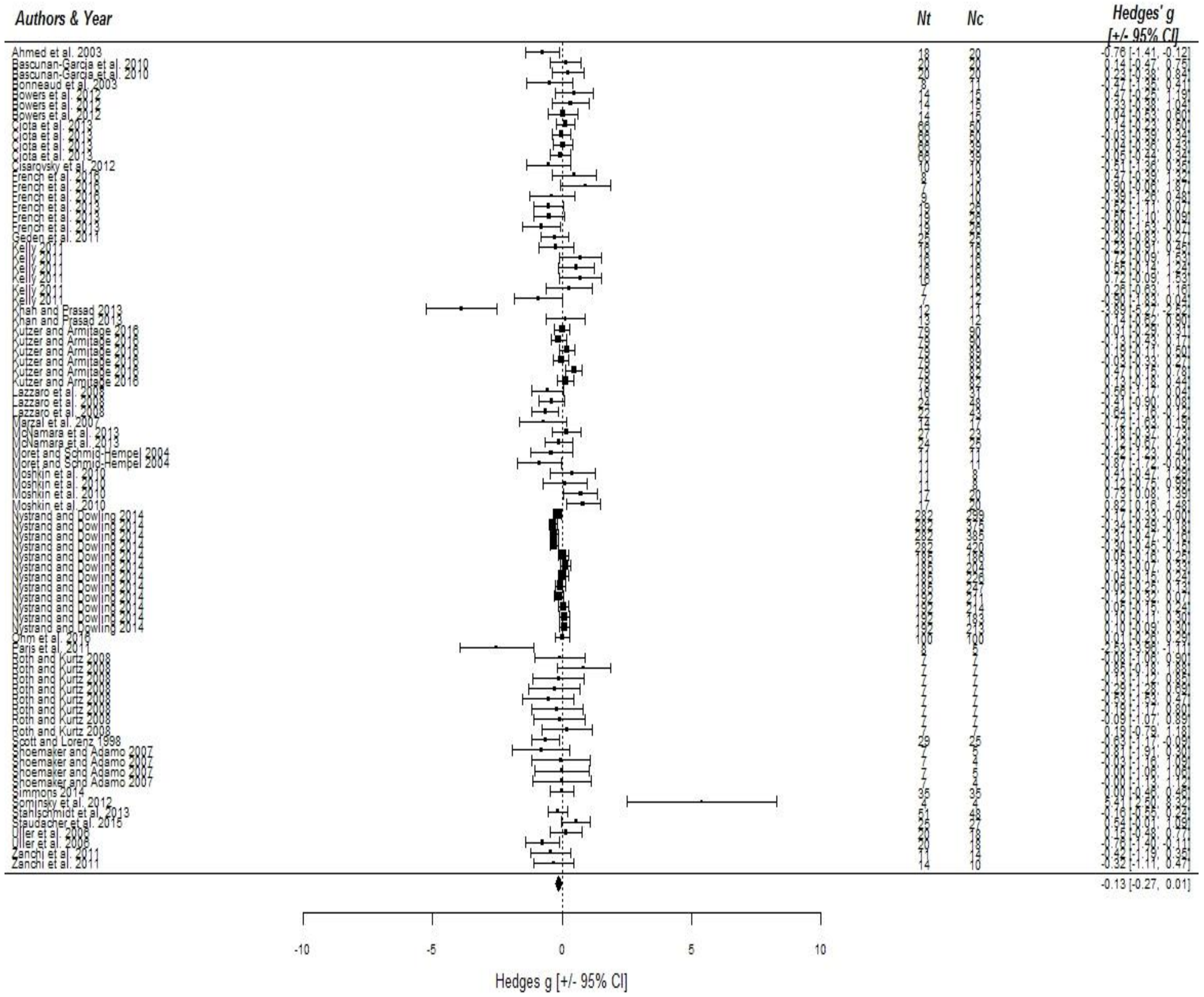

Figure S6. Forest plot of the non-phylogenetic meta-analytic model. Where values are < 0, the control group had higher reproductive success relative to the treated group, whereas values > 0 indicated the opposite.

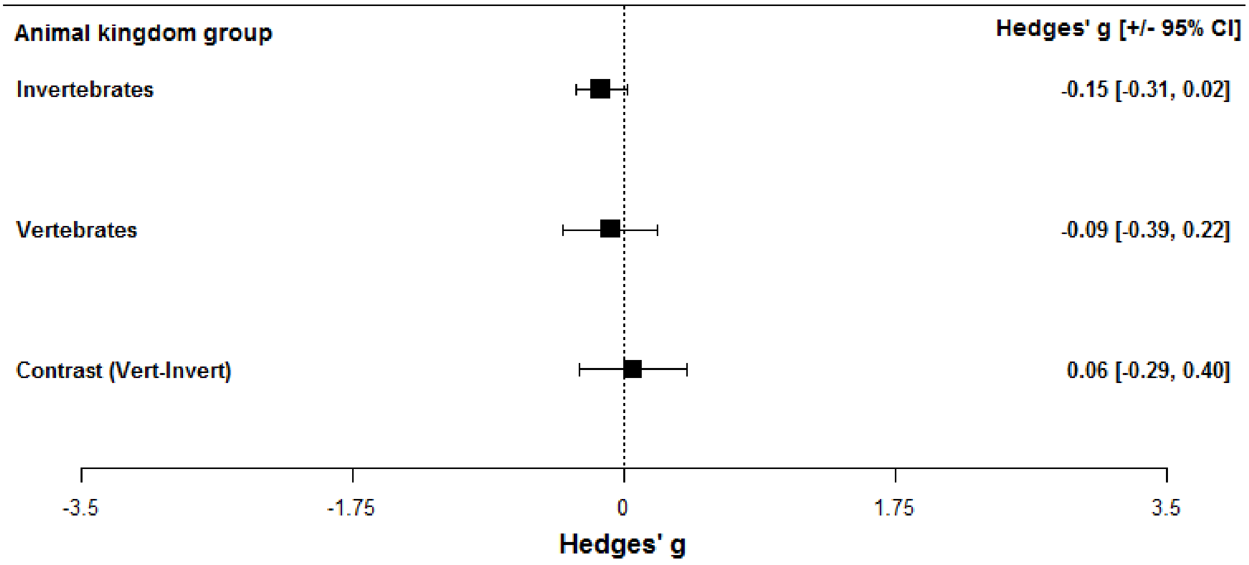

Figure S7. Forest plot showing the effect of major animal kingdom group on organismal reproduction, following an immune challenge.

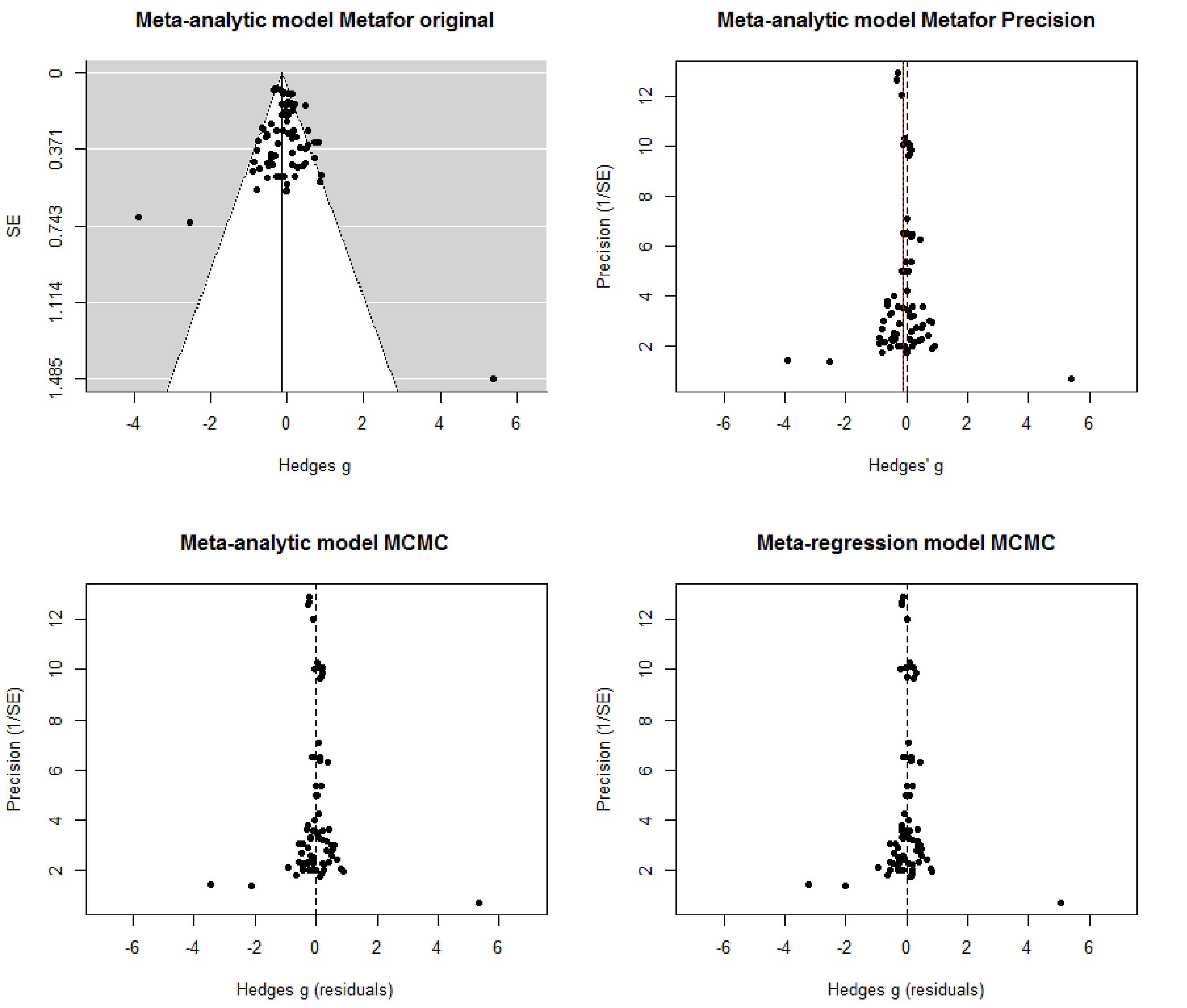

Figure S8. Funnel plot for reproductive success: upper left-hand panes shows the funnel plot from the original meta-analytic (*non-phylogenetic*) model generated in metafor (y-axis shows SE). Upper right hand panel shows the same data, but where the y-axis show precision (1/SE). For comparison, we also show the mean from the metafor model (red hatched line) and the posterior mean from the corresponding MCMCglmm model (solid black line). Lower left-hand panel illustrates the funnel plot from the meta-analytic model generated in MCMCglmm, but in which the x-axis display residuals and the y-axis precision (1/SE). Finally, the right-hand panel shows the corresponding model for the meta-regression data (main effects). Zero effect sizes (i.e. no effect of treatment) are plotted as hatched black lines intersecting zero [0] in all the modified funnel plots.

R^2 RESULTS FULL MODEL

Marginal: 15.51 %

Conditional: 80.97 %

Hence, random = 65.46 %

(Inclusion of animal kingdom in the model generates values of 14.88 % marginal, and 82.77 % conditional, hence 67.89 % from random factors)

ADDITIONAL FILE 1 – PROXIMATE IMMUNE TRAIT EXPRESSION

Table S17. Effect of all moderators and the interaction between life-history status and treatment agent on immune trait expression (***non-phylogenetic model***). Effect sizes used for statistical tests were Hedges' *g*. The reference level in the full model reflects adult females that were challenged with a non-replicating agent. AIC-values are generated from a ML model.

**MODERATORS** *(AIC =168.07)*	**ES (H*g*)**	**SE**	**CI LOWER**	**CI UPPER**
Life-history status				
Adult females	0.077	0.276	-0.463	0.617
Juveniles	-0.208	1.064	-2.293	1.878
Treatment agent				
Replicating agent	-0.050	0.332	-0.601	0.701
Life-history status x Treatment Agent				
Replicating agent x Adult females	-0.137	0.477	-1.072	0.799
Replicating agent x juvenile	0.206	1.428	-2.593	3.006

QM (df = 5) = 0.1703, p-val = 0.9994; interaction only: QM (df = 2) = 0.1169, p-val = 0.9432

Table S18. Effect of animal kingdom group (vertebrate or invertebrate) on immune trait expression. Effect sizes used for statistical tests were Hedges' *g*. Sample size of effect sizes for each group of the moderator is given in a separate column, followed by the associated number of studies in brackets.

**MODERATORS**	**ES (H*g*)**	**N_{ES} (studies)**	**SE**	**CI LOWER**	**CI UPPER**
Invertebrates	1.124	7 (4)	0.701	-0.250	2.498
Vertebrates	0.741	47 (12)	0.379	-0.002	1.484
Contrast (Vert-Invert)	0.383	--	0.797	-1.179	1.945

QM (df = 1) = 0.2310, p-val = 0.6308

Table S19. Effect of all moderators on immune trait expression (*non-phylogenetic model*), but where the effect of animal kingdom group (invertebrates or vertebrates) has been added to the model. Effect sizes used for statistical tests were Hedges' *g*. The reference level in the full model reflects adult females that were challenged with a non-replicating agent. AIC-values are generated from a ML model.

**MODERATORS** *(AIC = 166.00)*	**ES (H*g*)**	**SE**	**CI LOWER**	**CI UPPER**
Life-history status				
Adult females	0.038	0.239	-0.431	0.508
Juveniles	0.005	0.860	-1.681	1.690
**Treatment agent**				
Replicating agent	0.006	0.256	-0.496	0.508
**Animal Kingdom group**				
Vertebrates	0.381	0.887	-1.357	2.119

QM (df = 4) = 0.2417, p-val = 0.9933

Table S20. Effect of all moderators, including the interaction between life-history and treatment agent, on immune trait expression (***non-phylogenetic model***), but where the effect of animal kingdom group (invertebrates or vertebrates) has been added to the model. Effect sizes used for statistical tests were Hedges' *g*. The reference level in the full model reflects adult females that were challenged with a non-replicating agent. AIC-values are generated from a ML model.

**MODERATORS** *(AIC = 166.00)*	**ES (H*g*)**	**SE**	**CI LOWER**	**CI UPPER**
Life-history status				
Adult females	0.083	0.276	-0.458	0.624
Juveniles	-0.089	1.145	-2.332	2.155
**Treatment agent**				
Replicating agent	0.057	0.332	-0.595	0.708
**Animal Kingdom group**				
Vertebrates	0.388	0.929	-1.432	2.208
Life-history status x Treatment Agent				
Replicating agent x Female	-0.142	0.477	-1.078	0.793
Replicating agent x Juvenile	0.202	1.485	-2.709	3.113

QM (df = 6) = 0.3591, p-val = 0.9992; interaction only: QM (df = 2) = 0.1204, p-val = 0.9416

TABLE S21-24: ANALYSES IN WHICH PHYLOGENY HAS BEEN ACCOUNTED FOR

Table S21 (associated with Figure 2c). Effect of “life-history status” on immune trait expression (***phylogenetic model***). Since the majority of studies only assigned sex to adult individuals, sex and age were combined into one moderator consisting of three levels: adult females, adult males, and juveniles. Effect sizes used for statistical tests were Hedges’ *g*. Sample size of effect sizes for each group of the moderator is given in a separate column, followed by the associated number of studies in brackets.

**MODERATORS** *(AIC = 164.20)*	**ES (H*g*)**	**N_{ES} (studies)**	**SE**	**CI LOWER**	**CI UPPER**
*Adult female**	*0.880*	*24 (9)*	*0.406*	*0.085*	*1.675*
Adult male*	*0.843*	*20 (7)*	*0.416*	*0.029*	*1.658*
Juvenile	0.732	10 (4)	0.660	-0.562	2.025
Contrast (female-male)	0.037	--	0.234	-0.423	0.495
Contrast (juv-male)	-0.112	--	0.780	-1.640	1.417
Contrast (juv-female)	-0.148	--	0.775	-1.667	1.370

QF (df = 2) = 0.0541, p-val = 0.9733

Table S22 (associated with Figure 2c). Effect of “treatment agent” (replicating agent [bacteria and virus] vs. non-replicating agent [heat-killed bacteria or virus, LPS, implant]) on immune trait expression (***phylogenetic model***). Effect sizes used for statistical tests were Hedges’ *g.* Sample size of effect sizes for each group of the moderator is given in a separate column, followed by the associated number of studies in brackets.

**MODERATORS** *(AIC = 162.25)*	**ES (H*g*)**	**N_{ES} (studies)**	**SE**	**CI LOWER**	**CI UPPER**
*Replic. agent**	*0.822*	*14 (4)*	*0.380*	*0.078*	*1.566*
*Non-replic. Agent**	*0.826*	*40 (14)*	*0.327*	*0.185*	*1.468*
Contrast (Replic. –Non-replic.)	-0.004	--	0.248	-0.490	0.482

QM (df = 1) = 0.0003, p-val = 0.9859

Table S23 (associated with Figure 3c). Effect of all moderators (main effects only) on immune trait expression (***phylogenetic model***). Effect sizes used for statistical tests were Hedges' *g*. The reference level in the full model reflects adult females that were challenged with a non-replicating agent. AIC-values are generated from a ML model.

**MODERATORS** (AIC =166.20)	**ES (Hg)**	**SE**	**CI LOWER**	**CI UPPER**
**Life-history status**				
Adult females	0.035	0.239	-0.434	0.503
Juveniles	-0.110	0.789	-1.656	1.436
**Treatment agent**				
Replicating agent	0.002	0.255	-0.499	0.503

QM (df = 3) = 0.0490, p-val = 0.9972

Table S24. Effect of all moderators and the interaction between life-history status and treatment agent on immune trait expression (***phylogenetic model***). Effect sizes used for statistical tests were Hedges' *g*. The reference level in the full model reflects adult females that were challenged with a non-replicating agent. AIC-values are generated from a ML model.

**MODERATORS** *(AIC = 170.07)*	**ES (H*g*)**	**SE**	**CI LOWER**	**CI UPPER**
Life-history status				
Adult females	0.077	0.276	-0.463	0.617
Juveniles	-0.208	1.064	-2.293	1.878
Treatment agent				
Replicating agent	-0.050	0.332	-0.601	0.701
Life-history status x Treatment Agent				
Replicating agent x Adult females	-0.137	0.477	-1.072	0.799
Replicating agent x Juvenile	0.206	1.428	-2.593	3.006

QM (df = 5) = 0.1703, p-val = 0.9994; interaction only: QM (df = 2) = 0.1169, p-val = 0.9432

FIGURES

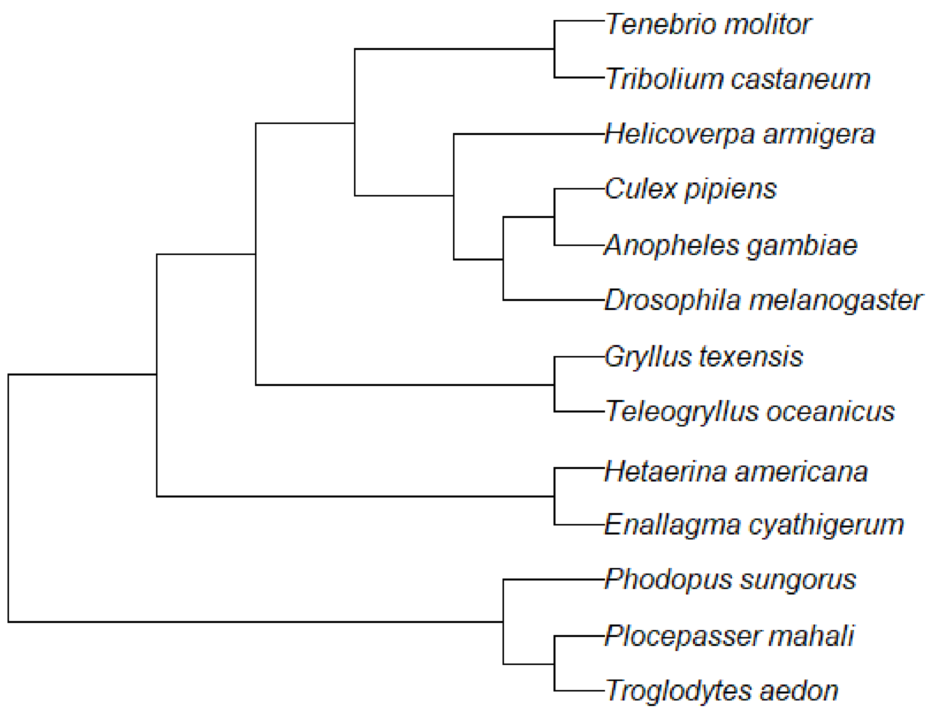

Figure S9. Phylogenetic tree of studies analysed for immune trait expression, modified in Mesquite.

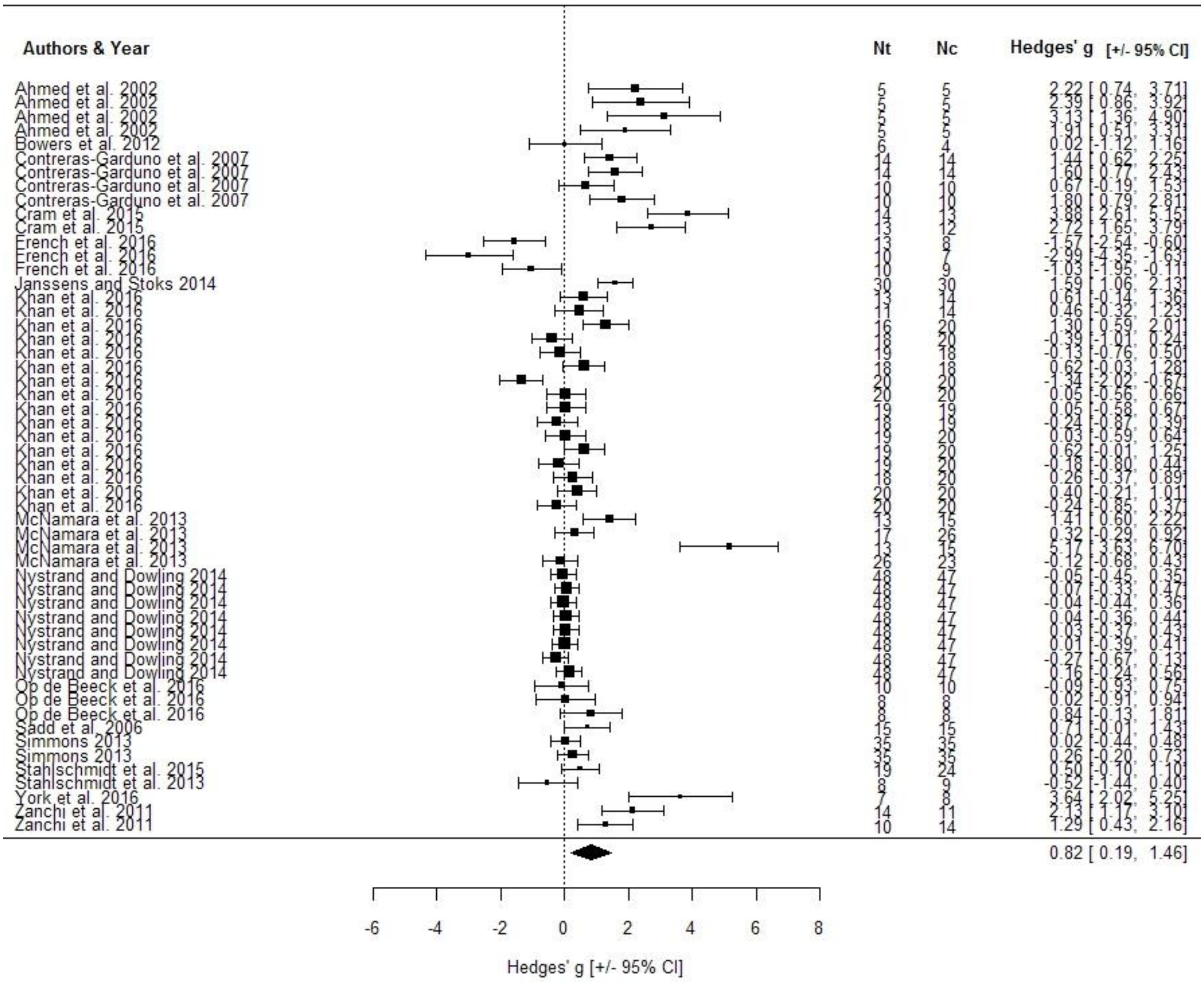

Figure S10. Forest plot of the non-phylogenetic meta-analytic model (immune trait expression).

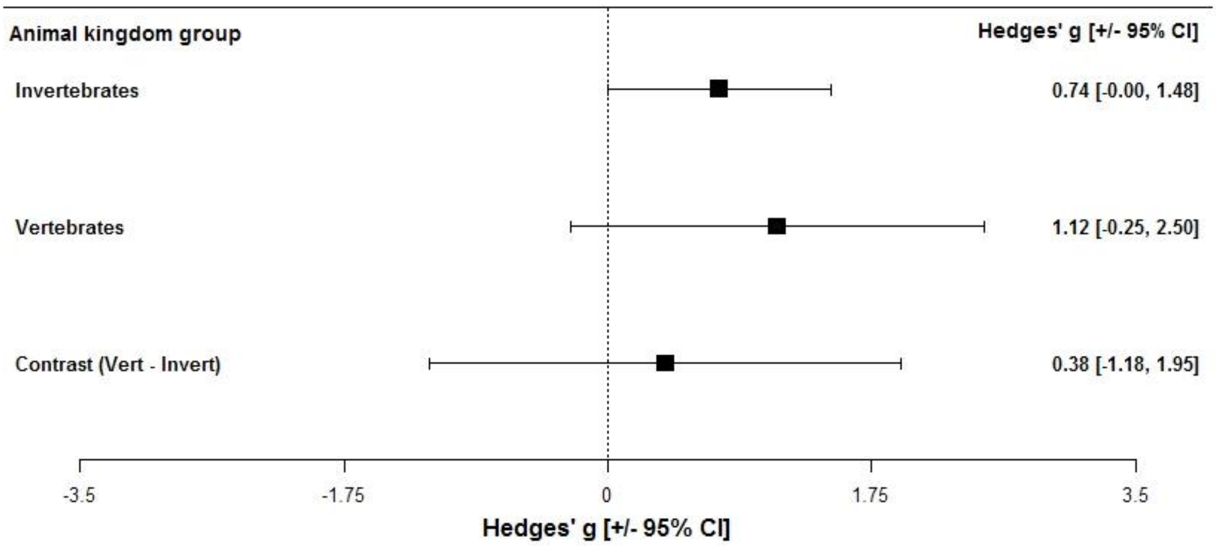

Figure S11. Forest plot showing the effect of major animal kingdom group on immune trait expression, following an immune challenge.

SUBSAMPLE ANALYSIS EXPLORING THE EFFECT OF IMMUN ASSAY VARIABLE

Table S25. Effect of “immune trait” (PO or antimicrobial activity) and life-history stage on immune trait expression following an immune challenge (*non-phylogenetic model*). Effect sizes used for statistical tests were Hedges’ *g*. The reference level in the full model reflects adult males that were assayed for PO and challenged with a non-replicating immune agent. None of the interactions (treatment agent x immune variable or life-history x immune variable contributed to explain host immune trait expression, and are therefore not displayed in the table).

MODERATORS	ES (H*g*)	SE	CI LOWER	CI UPPER
**Life-history status**				
Adult females	0.055	0.214	-0.364	0.475
Juveniles	-0.096	0.764	-1.593	1.402
**Immune variable**				
*Antimicrobial activity**	*1.533*	*0.459*	*0.633*	*2.433*
**Treatment Agent**				
Replicating agent	0.007	0.216	-0.416	0.429

QM(df = 4) = 11.3669, p-val = 0.0227

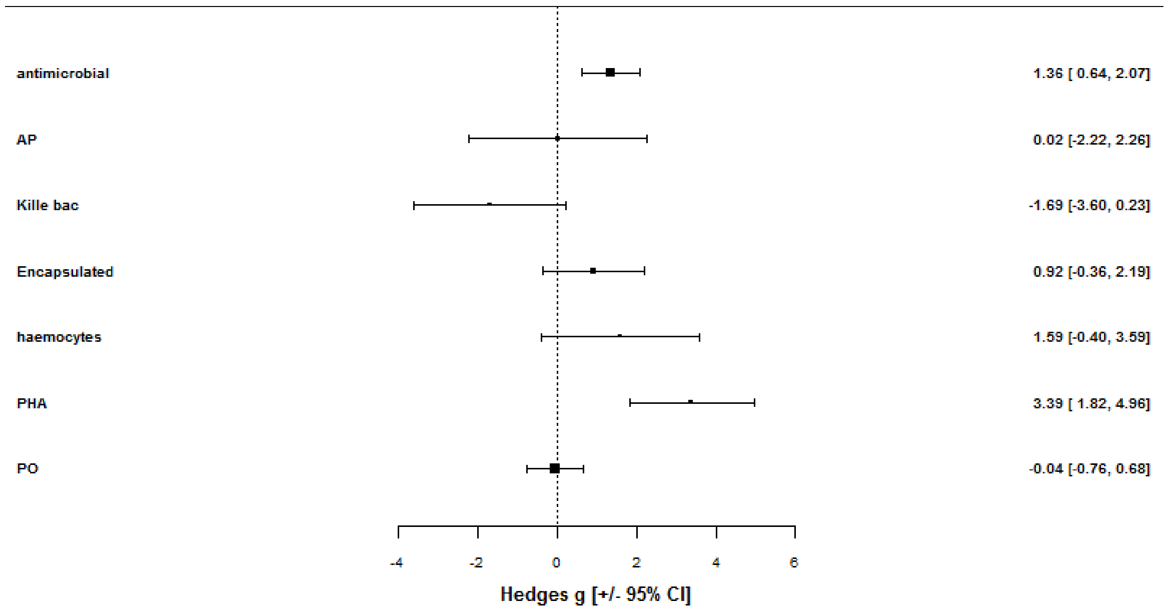

Figure S12a. Forest plot of effect sizes for all immune traits.

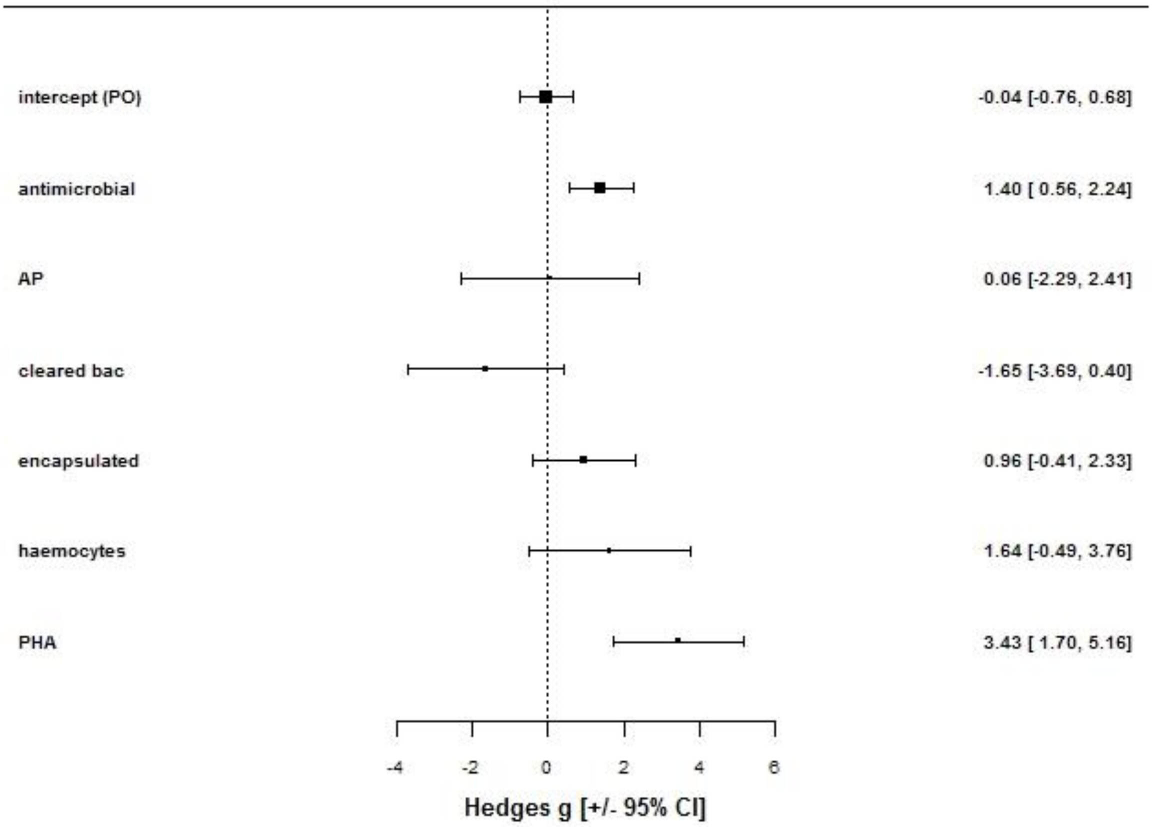

Figure S12b. Forest plot of contrast between effect sizes for all immune traits (reference level is antimicrobial activity).

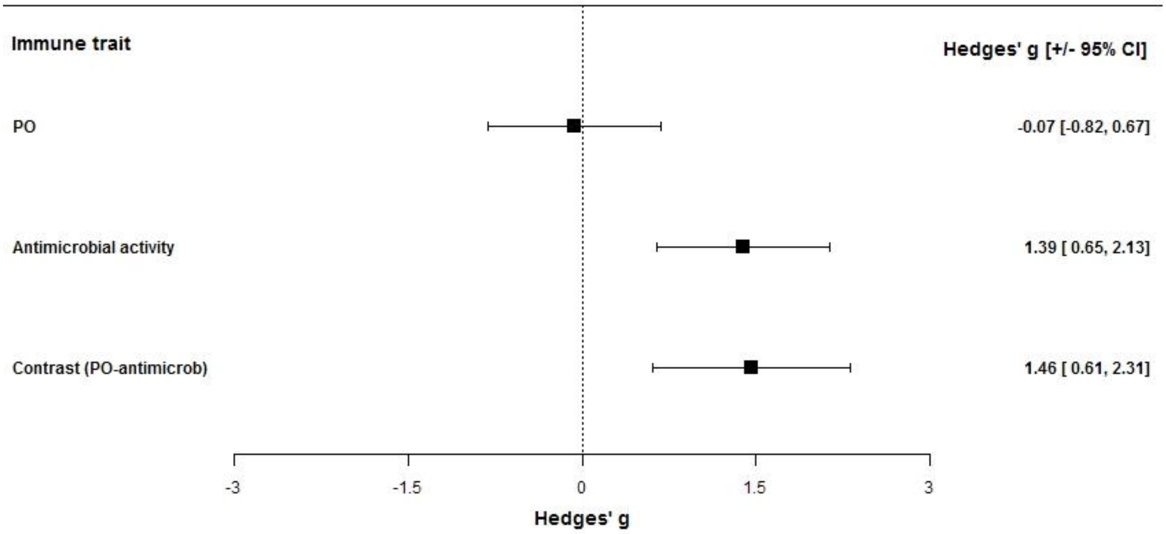

Figure S13. Forest plot of the subset of immune trait expression data (only including PO and antimicrobial activity).

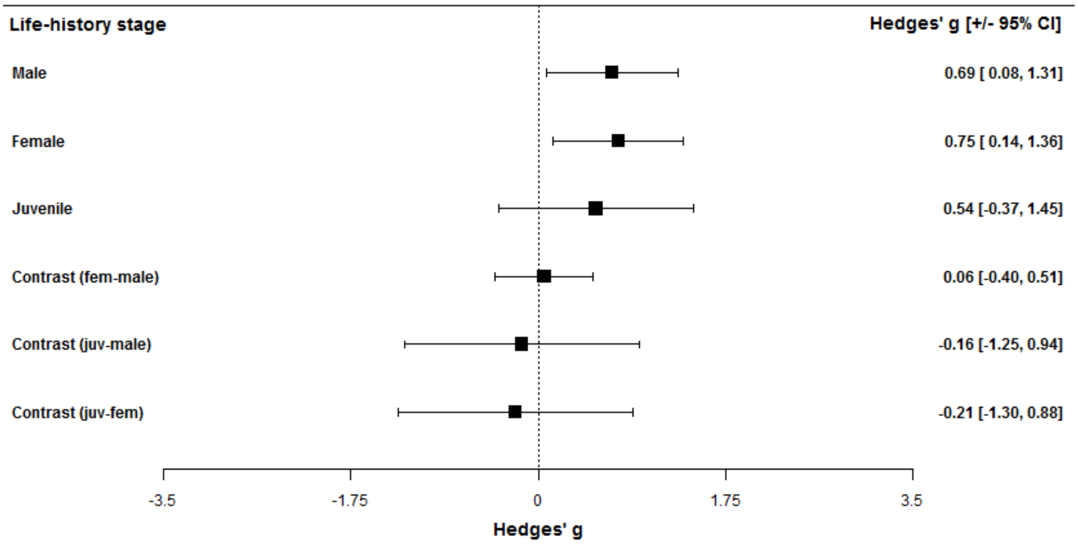

Figure S14. Forest plot showing the effect of life-history status on the subset of immune trait expression data (only including PO and antimicrobial activity).

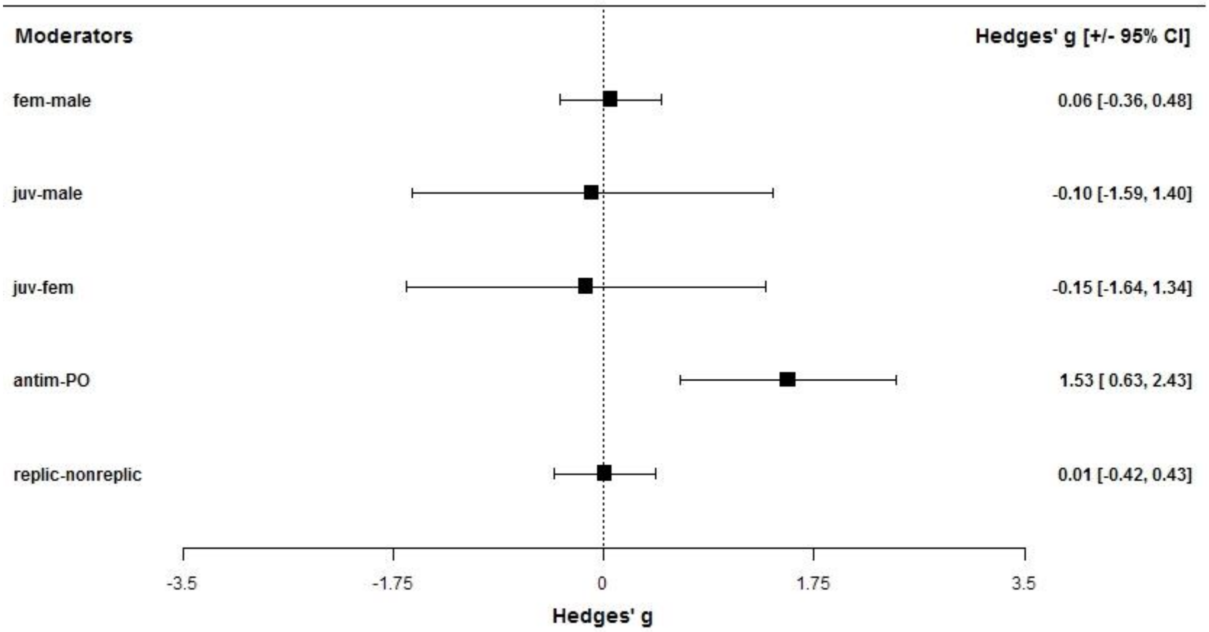

Figure S15. Full meta-regression forest plot of immune trait expression subset data containing antimicrobial activity and PO (reference level = males, PO, intercept = -0.096 ± SE 0.480, CI = -1.036 – 0.844).

Forest plots full data set

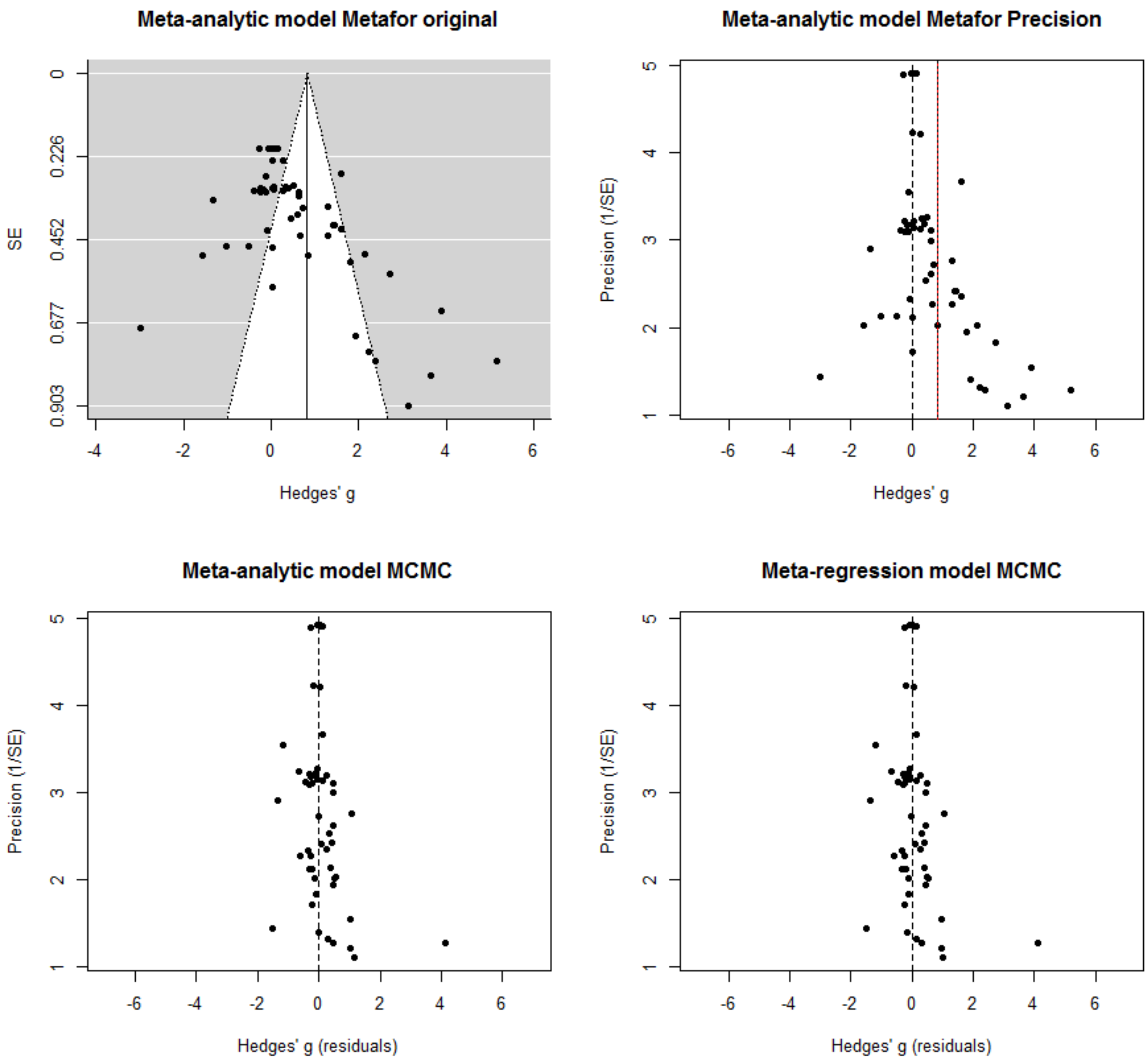

Figure S16. Upper left-hand panes shows the funnel plot from the original meta-analytic (non-phylogenetic) model generated in metaphor (SE displayed on y-axis). Upper right hand panel shows the same data, but where the y-axis illustrate precision (1/SE). For comparison, we also show the mean from the metafor model (red hatched line) and the posterior mean from the corresponding MCMCglmm model (hatched black line). Lower left-hand panel illustrates the funnel plot from the meta-analytic model generated in MCMCglmm, but in which the x-axis display residuals and the y-axis precision (1/SE). Finally, the right-hand panel shows the corresponding model for the meta-regression data (main effects). Zero effect sizes (i.e. no effect of treatment) are plotted as solid black lines intersecting zero [0] in all the modified funnel plots.

R^2 RESULTS FULL MODEL

Marginal: 0.14 %

Conditional: 88.14 %

Random = 88.00 %

(*Inclusion of animal kingdom in the model generates values of 0.66 % marginal, and 89.38 % conditional, hence 88.72 % from random factors)

ADDITIONAL FILE 1 - MORPHOLOGY

Table S26. Effect of major animal kingdom group (vertebrate *vs.* invertebrate) on morphology. Effect sizes used for statistical tests were Hedges' *g*. Sample size of effect sizes for each group of the moderator is given in a separate column, followed by the associated number of studies in brackets.

**MODERATORS** *(AIC = 68.53)*	**ES (H*g*)**	**N_{ES} (studies)**	**SE**	**CI LOWER**	**CI UPPER**
Vertebrates	0.289	12 (7)	0.174	-0.053	0.630
Invertebrates	-0.121	19 (8)	0.146	-0.407	0.165
Contrast (Vert-Invert)	0.410	--	0.227	-0.036	0.855

QM(df = 1) = 3.2501, p-val = 0.0714

Table S27. Effect of all moderators on morphology (*non-phylogenetic model*), but where the effect of animal kingdom group (vertebrate *vs*. invertebrate) has been added to the model. Effect sizes used for statistical tests were Hedges' *g*. The reference level in the full model reflects adult females that were challenged with a non-replicating agent. AIC-values are generated from a ML model.

**MODERATORS** *(AIC = 66.20)*	**ES (H*g*)**	**SE**	**CI LOWER**	**CI UPPER**
Life-history status				
Adult females	0.430	0.390	-0.335	1.195
Juveniles	0.068	0.334	-0.587	0.722
Treatment agent				
Replicating agent	0.189	0.371	-0.537	0.915
Animal kingdom group				
Vertebrates	0.435	0.314	-0.181	1.051

QM(df = 4) = 3.4710, p-val = 0.4823

* all vertebrates were challenged with non-replicating agents only, so interpret with caution

TABLE S28-S30 ANALYSES IN WHICH PHYLOGENY HAS BEEN ACCOUNTED FOR

Table S28 (associated with Figure 2d). Effect of "life-history status" on morphology (***phylogenetic model***). Since the majority of studies only assigned sex to adult individuals, sex and age were combined into one moderator consisting of three levels: adult females, adult males, and juveniles. Effect sizes used for statistical tests were Hedges' *g*. Sample size of effect sizes for each group of the moderator is given in a separate column, followed by the associated number of studies in brackets.

**MODERATORS** *(AIC = 66.59)*	**ES (Hg)**	**N_{ES} (studies)**	**SE**	**CI LOWER**	**CI UPPER**
Adult female	0.266	7 (5)	0.321	-0.363	0.894
Adult male	-0.037	5 (4)	0.350	-0.722	0.649
Juvenile	0.041	19 (6)	0.276	-0.582	0.500
Contrast (fem-male)	0.303	--	0.338	-0.361	0.966
Contrast (juv-male)	0.005	--	0.297	-0.586	0.577
Contrast (juv-female)	-0.307	--	0.268	-0.831	0.217

QM(df = 2) = 1.4220, p-val = 0.4912

Table S29 (associated with Figure 2d). Effect of "treatment agent" (replicating agent [bacteria and virus] vs. non-replicating agent [heat-killed bacteria or virus, LPS, implant]) on morphology (***phylogenetic model***). Effect sizes used for statistical tests were Hedges' *g.* Sample size of effect sizes for each group of the moderator is given in a separate column, followed by the associated number of studies in brackets.

**MODERATORS** *(AIC = 72.38)*	**ES (Hg)**	**N_{ES} (studies)**	**SE**	**CI LOWER**	**CI UPPER**
Replic. agent	-0.043	12 (3)	0.440	-0.906	0.819
Non-replic. Agent	0.115	19 (12)	0.318	-0.561	0.692
Contrast (Replic. –Non-replic.)	-0.109		0.435	-0.962	0.744

QM(df = 1) = 0.0624, p-val = 0.8028

Table S30 (associated with Figure 3d). Effect of all moderators on morphology (***phylogenetic model***). Effect sizes used for statistical tests were Hedges' g. The reference level in the full model reflects adult females that were challenged with a non-replicating agent. AIC-values are generated from a ML model.

**MODERATORS** *(AIC = 68.22)*	**ES (Hg)**	**SE**	**CI LOWER**	**CI UPPER**
**Life-history status**				
Adult females	0.292	0.353	-0.401	0.985
Juveniles	-0.023	0.306	-0.624	0.578
**Treatment agent**				
Replicating agent	0.058	0.464	-0.851	0.967

QM(df = 3) = 1.2905, p-val = 0.7314

FIGURES

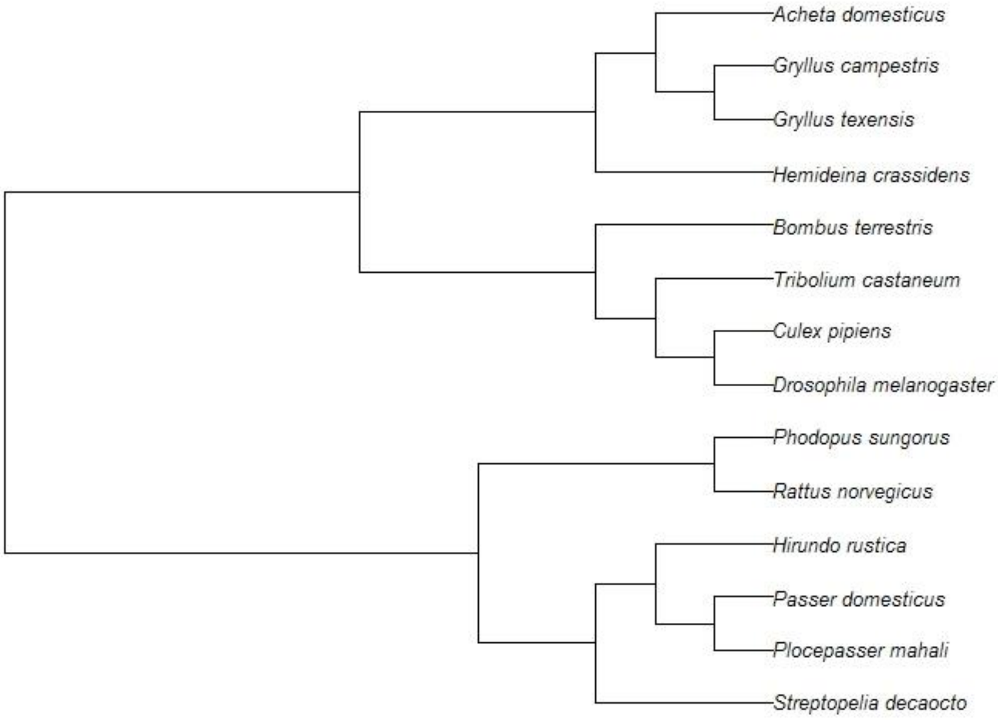

Figure S17. Phylogenetic tree of study species included in the analysis of morphological traits, modified in Mesquite.

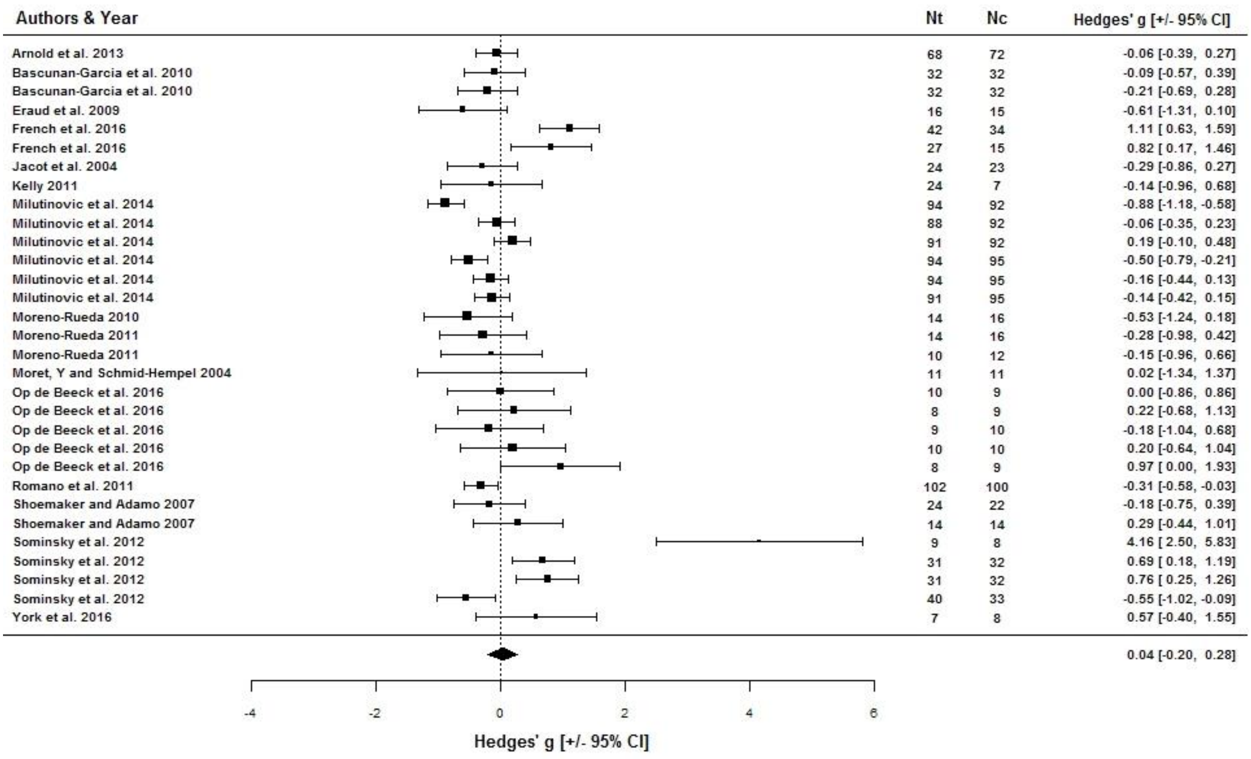

Figure S18. Forest plot of the non-phylogenetic meta-analytic model. Where values are < 0, the control group had larger morphological trait expression relative to the treated group, whereas values > 0 indicated the opposite.

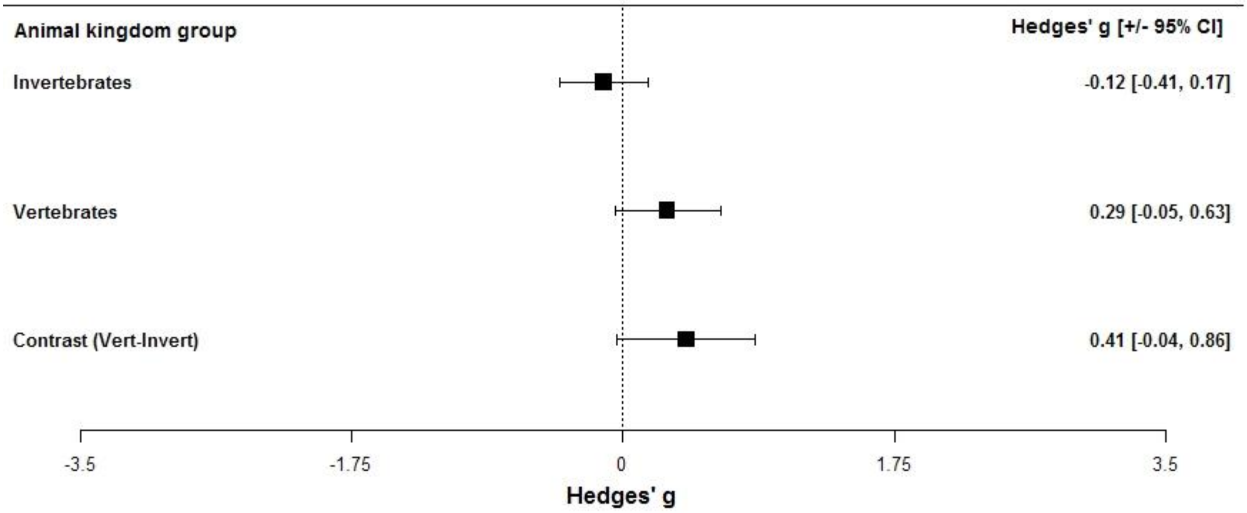

Figure S19. Forest plot showing the effect of the major animal kingdom group on morphology, following an immune challenge.

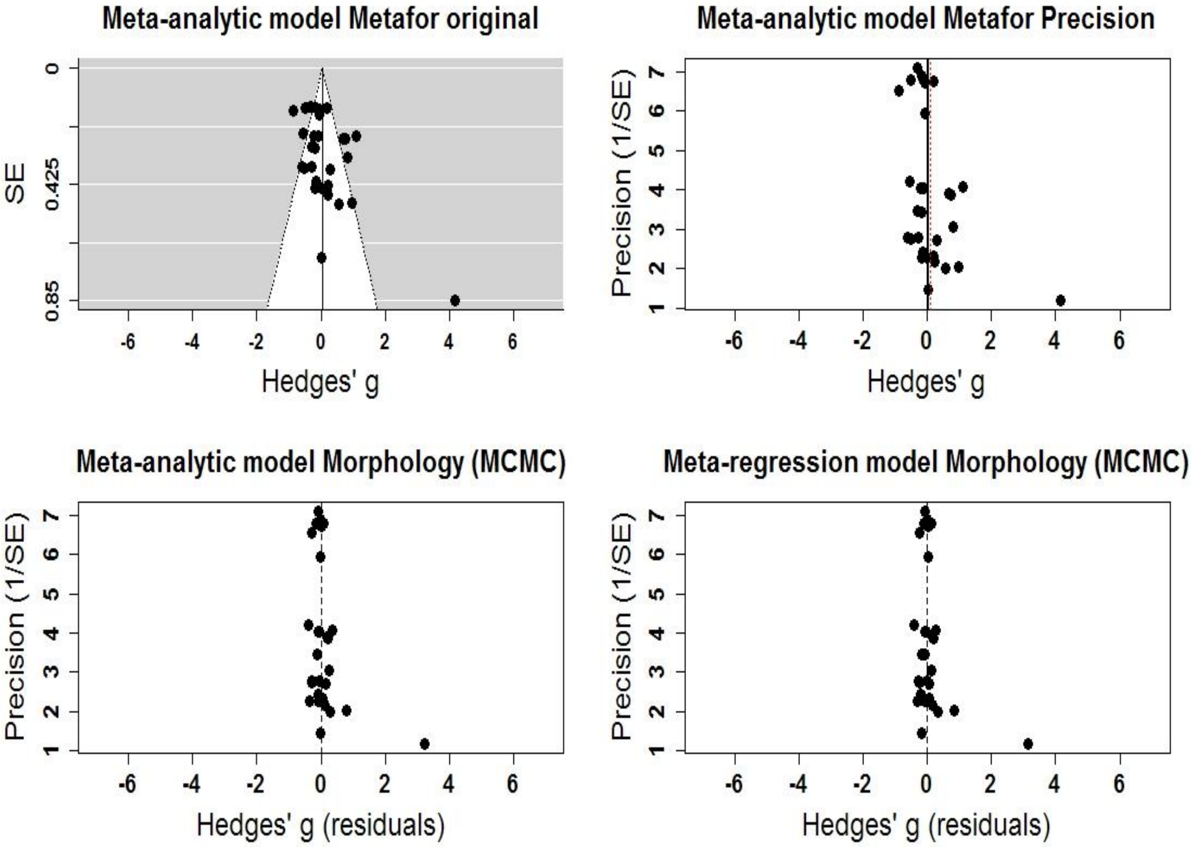

Figure S20. Upper left-hand panes shows the funnel plot from the original meta-analytic (non-phylogenetic) model generated in metaphor (SE displayed on y-axis). Upper right hand panel shows the same data, but where the y-axis illustrate precision (1/SE). For comparison, we also show the mean from the metafor model (red hatched line) and the posterior mean from the corresponding MCMCglmm model (solid black line). Lower left-hand panel illustrates the funnel plot from the meta-analytic model generated in MCMCglmm, but in which the x-axis display residuals and the y-axis precision (1/SE). Finally, the right-hand panel shows the corresponding model for the meta-regression data (main effects). Zero effect sizes (i.e. no effect of treatment) are plotted as hatched black lines intersecting zero [0] in all the modified funnel plots.

R^2 RESULTS FULL MODEL

Marginal: 4.52 %

Conditional: 83.83 %

Hence, random = 78.81 %

ADDITIONAL FILE 1 – DEVELOPMENT TIME

TABLE S31: ANALYSIS IN WHICH PHYLOGENY HAS BEEN ACCOUNTED FOR

Table S31. Effect of "treatment agent" (replicating agent [bacteria and virus] vs. non-replicating agent [heat-killed bacteria or virus, LPS, implant]) on development times (***phylogenetic model***). Effect sizes used for statistical tests were Hedges' *g.* Sample size of effect sizes for each group of the moderator is given in a separate column, followed by the associated number of studies in brackets.

**MODERATORS** *(AIC = 30.44)*	**ES (Hg)**	**N_{ES} (studies)**	**SE**	**CI LOWER**	**CI UPPER**
Replic. agent	-0.034	11 (3)	0.347	-0.714	0.645
Non-replic. Agent	-0.203	13 (3)	0.321	-0.831	0.426
Contrast (Replic. –Non-replic.)	0.168	--	0.228	-0.278	0.614

QM(df = 1) = 0.5460, p-val = 0.4600

FIGURES

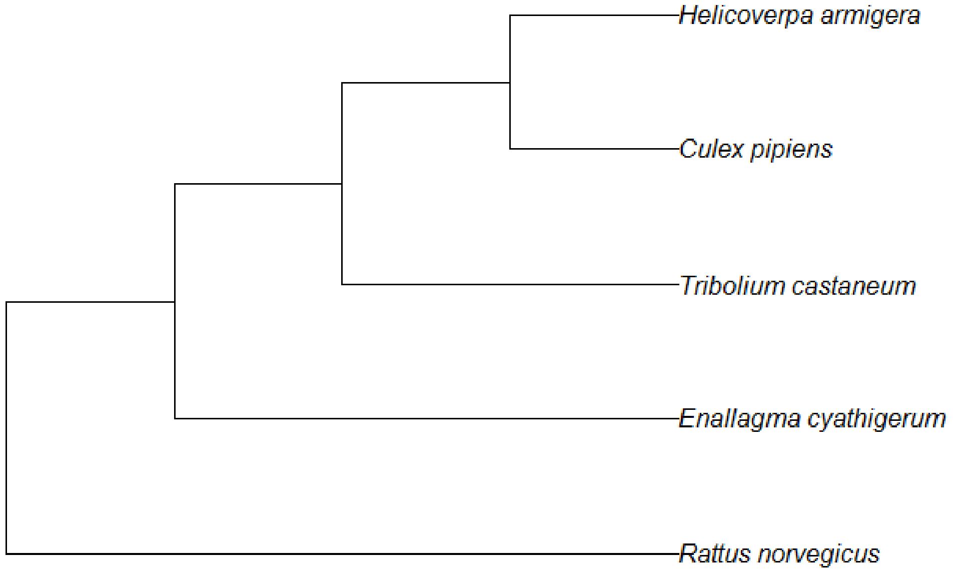

Figure S21. Phylogenetic tree of study species included in the analysis of development times, modified in Mesquite.

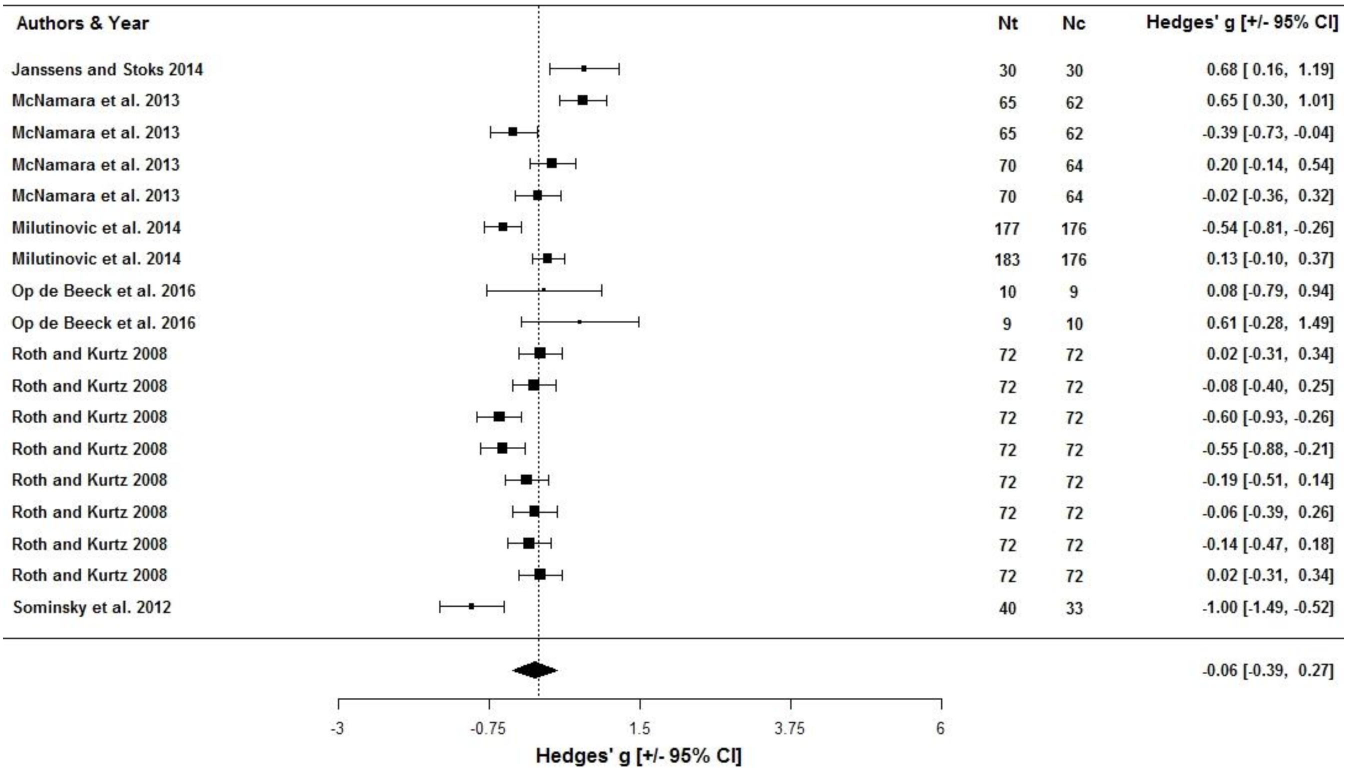

Figure S22. Forest plot of the non-phylogenetic meta-analytic model.

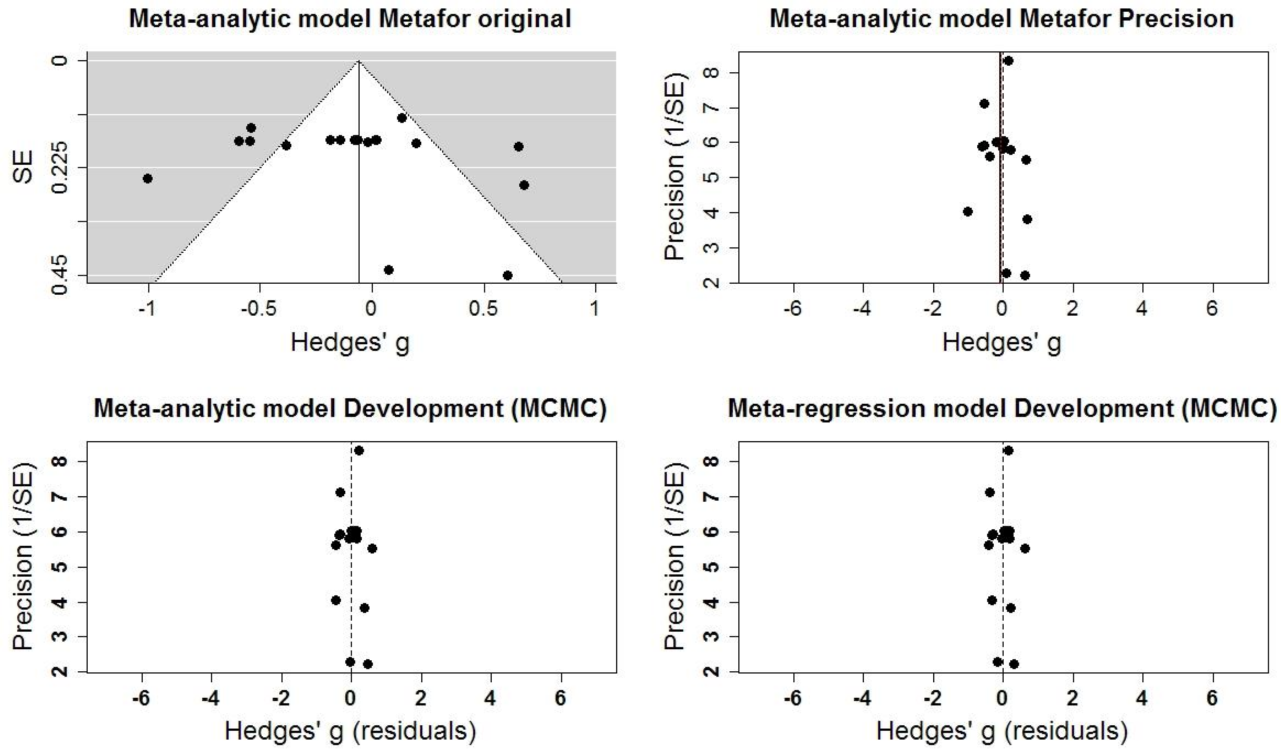

Figure S23. Upper left-hand panes shows the funnel plot from the original meta-analytic (non-phylogenetic) model generated in metafor (SE displayed on y-axis). Upper right hand panel shows the same data, but where the y-axis illustrate precision (1/SE). For comparison, we also show the mean from the metafor model (red hatched line) and the posterior mean from the corresponding MCMCglmm model (solid black line). Lower left-hand panel illustrates the funnel plot from the meta-analytic model generated in MCMCglmm, but in which the x-axis display residuals and the y-axis precision (1/SE). Finally, the right-hand panel shows the corresponding model for the meta-regression data (main effects). Zero effect sizes (i.e. no effect of treatment) are plotted as hatched black lines intersecting zero [0] in all the modified funnel plots.

R^2 RESULTS FULL MODEL

Marginal: 5.90 %

Conditional: 88.87 %

Random = 82.97 %

ADDITIONAL FILE 1 : Mating subset data, females only

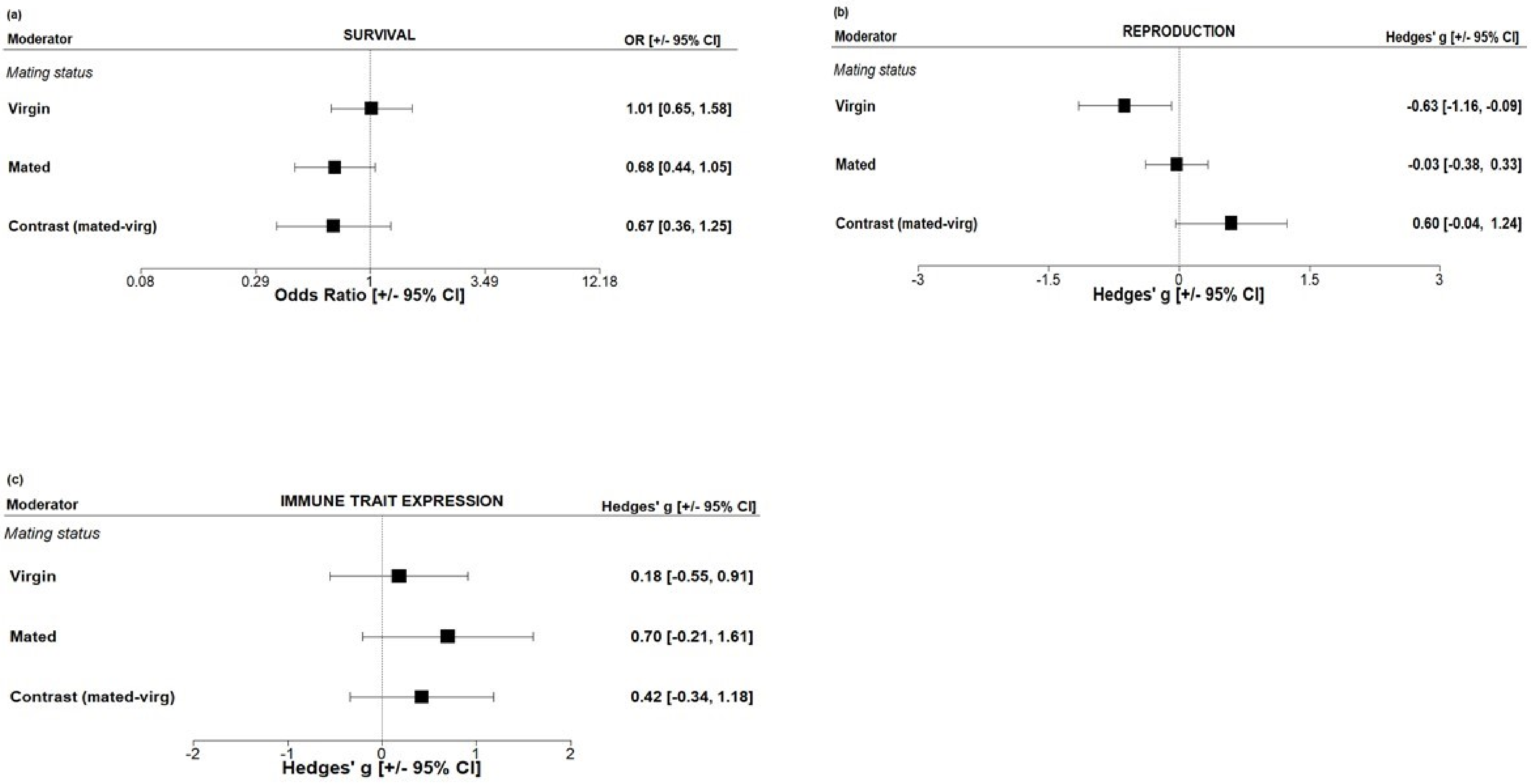

Figure S24. Mating subset only including females.

ADDITIONAL FILE 1 -

Detailed discussion on comparison with previous meta-analysis exploring proximate immune expression in animals

---

It is worth pointing out that there are some fundamental differences in the approach taken by the two previous meta-analyses addressing sex differences in immune trait expression and ours. The first key difference between the data in our study and those of Nunn *et al.* (2009) and Kelly et al. (2018), is that our study specifically explored – and was limited to – studies of immune deployment, hence excluding data on immune maintenance (i.e. background levels of immune trait expression that are measured in the absence of any immune challenge). In contrast, the two previous meta-analyses assessed their immune trait parameters from studies that consisted of a mix of maintenance and deployment immune measures [however, with Kelly *et al.* (2018) conducting a formal comparison between the two types – deployment versus maintenance]. Moreover, neither Nunn *et al.* (2009) nor Kelly *et al.* (2018) based their analysis exclusively on studies that were designed using a challenge and a control, but rather, effect sizes were based on direct comparison between sexes (i.e. female relative to male value rather than treatment relative to control). In contrast, only 15 % of our sampled articles measured females and males within the same study (8 of 52). This is because many studies directly addressing female-male differences did not qualify for our selection criterion, whereby all treatments had to be associated with a procedural control. Such divergence in the approach between studies is important to acknowledge, because immune deployment following an immune challenge is likely to produce different cost dynamics compared to those associated with maintenance [1, 2], and this can vastly influence the interpretation of data. As an example to illustrate this point, a recent study exploring immune-induced effects on gene-expression and fitness in a moth (*Heliothis virescens*) found higher expression levels of candidate immune genes in females

following a bacterial challenge compared to males, and this was also correlated with a reduction in fitness-related traits. In contrast, males had higher constitutive expression levels (background levels) of these same genes, and their fitness-related traits were unaffected by an immune challenge. Thus, it is possible that the acute costs are higher in females, but that the ongoing maintenance costs are higher in males [1].

The second key difference relates to our main analysis on proximate immune trait expression; here, we addressed overall immune trait expression, regardless of which specific component of the immune system that had been assayed in a particular study. In contrast, both earlier meta-analyses investigated two (Nunn *et al.* 2009) or many (Kelly *et al.* 2018) specific immune traits. Indeed, divergence across different immune traits is common, and different immune traits have been found to display positive correlations, no correlation, or negative correlations with each other [3-7]. Thus, to further explore the possibility that negative correlations between immune traits were behind the lack of sex-specific immune response in our data, we conducted an additional analysis on immune trait expression in which we separated out the different immune variables measured. This analysis showed that all traits were either unaffected (confidence interval intersecting zero: antibody production [AP], bacteria cleared, encapsulation, haemocyte number, PO), or were upregulated following an immune challenge (antimicrobial activity, PHA; Supp mat C, Fig. S6a-b). Likewise, a more detailed exploration of the two traits containing sufficient data (conducted in insects only) followed similar patterns to those recorded for the full data sat containing both vertebrates and invertebrates (in insects, antimicrobial activity but not PO, was upregulated following an immune challenge). No sex or age-specific effects were detected in the full data set, nor in the analysis limited to invertebrates. Likewise, there was no evidence that immune trait expression was influenced by the treatment agent

administered in either analysis. Nevertheless, both our study and those by others reinforce the pitfalls associated with focusing only on one or a few proximate immune traits, when attempting to estimate the costs of immunity.

References

1. Barthel A, taudacher H, Schmaltz A, Heckel DG, Groot AT: **Sex-specific consequences of an induced immune response on reproduction in a moth**. *BMC Evol Biol* 2015, **15**(1):282.
2. McKean K, Yourth C, Lazzaro B, Clark A: **The evolutionary costs of immunological maintenance and deployment**. *BMC Evol Biol* 2008, **8**(1):76.
3. Adamo SA: **How should behavioural ecologists interpret measurements of immunity?** *Anim Behav* 2004, **68**:1443-1449.
4. Graham AL, Shuker DM, Pollitt LC, Auld S, Wilson AJ, Little TJ: **Fitness consequences of immune responses: strengthening the empirical framework for ecoimmunology**. *Funct Ecol* 2011, **25**(1):5-17.
5. Elrod-Erickson M, Mishra S, Schneider D: **Interactions between the cellular and humoral immune responses in *Drosophila***. *Curr Biol* 2000, **10**(13):781-784.
6. Nehme NT, Quintin J, Cho JH, Lee J, Lafarge M-C, Kocks C, Ferrandon D: **Relative Roles of the Cellular and Humoral Responses in the <italic>Drosophila</italic> Host Defense against Three Gram-Positive Bacterial Infections**. *PLoS One* 2011, **6**(3):e14743.
7. Cotter S, Kruuk L, Wilson K: **Costs of resistance: genetic correlations and potential trade-offs in an insect immune system**. *J Evol Biol* 2004, **17**:421 - 429.
