# Supplementary material for: Effects of immune challenge on expression of life-history and immune trait expression in sexually reproducing metazoans—a meta-analysis
Source: BMC Biol. 2020 Oct 7;18:135. doi: 10.1186/s12915-020-00856-7 (PMC7541220; doi:10.1186/s12915-020-00856-7)
Supplement: Supplementary file 2 — Additional file 2. Supplementary source data tables associated with the four main figures presented in the manuscript. Tables associated with Fig. 2 - meta-analyses (Table A2.S1_1 – A2.S5_1). Tables associated with Fig. 3 - meta-regressions (Table A2.S6 - A2.S9). Tables associated with Fig. 4 - meta-regression subsample for mating status (Table A2.S10 – A2.S12). [file 12915_2020_856_MOESM2_ESM.pdf]

ADDITIONAL FILE 2 – Statistical tables of the source data depicted in the main Figures 1-4.

<u>Tables associated with Figure 2 - meta-analysis:</u>

- Table A2.S1_1. This is the source data to Figure 2a for the effect of “life-history status” on survival (non-phylogenetic model).
- Table A2.S1_2. This is the source data to Figure 2a for the effect of “treatment agent” (replicating agent vs. non-replicating agent) on survival (non-phylogenetic model).
- Table A2.S2_1. This is the source data to Figure 2b for the effect of “life-history status” on reproduction (non-phylogenetic model).
- Table A2.S2_2. This is the source data to Figure 2b for the effect of “treatment agent” (replicating agent vs. non-replicating agent) on reproduction (non-phylogenetic model).
- Table A2.S3_1. This is the source data to Figure 2c for the effect of “life-history status” on immune trait expression nonphylogenetic model).
- Table A2.S3_2. This is the source data to Figure 2c for the effect of “treatment agent” (replicating agent vs. non-replicating immune agent) on immune trait expression (non-phylogenetic model).
- Table A2.S4_1. This is the source data to Figure 2d for the effect of “life-history status” on morphology (non-phylogenetic model).
- Table A2.S4_2. This is the source data to Figure 2d for the effect of “treatment agent” (replicating agent vs. non-replicating agent) on morphology (*non-phylogenetic model*).
- Table A2.S5_1. This is the source data to Figure 2e for the effect of “treatment agent” (replicating agent vs. non-replicating agent) on development times (non-phylogenetic model).

<u>Tables associated with Figure 3 - meta-regressions:</u>

- Table A2.S6. This is the source data to Figure 3a for the effect of all moderators on survival (non-phylogenetic model).
- Table A2.S7. This is the source data to Figure 3b for the effect of all moderators on reproduction (non-phylogenetic model).
- Table A2.S8. This is the source data to Figure 3c for the wffect of all moderators (main effects only) on immune trait expression (nonphylogenetic model).
- Table A2.S9. This is the source data to Figure 3d for the effect of all moderators on morphology (non-phylogenetic model).

<u>Tables associated with Figure 4 - meta-regression subsample for mating status:</u>

- Table A2.S10. This is the source data to Figure 4a for the effect of “mating status” (virgin or mated) on survival.
- Table A2.S11. This is the source data to Figure 4b for the effect of “mating status (virgin or mated) on reproduction.
- Table A2.S12. This is the source data to Figure 4c for the effect of “mating status (virgin or mated) on immune trait expression.

Statistical tables of the source data depicted in the main Figures 1-4

Tables associated with Figure 2 - meta-analyses

Table A2.S1_1. This is the source data to Figure 2a for the effect of "life-history status" on survival (*non-phylogenetic model*). Since the majority of studies only assigned sex to adult individuals, sex and age were combined into one moderator consisting of three levels: adult females, adult males, and juveniles. Effect sizes used for statistical tests were lnOR. However, back-transformed values (OR) are given in brackets. Sample size of effect sizes for each group of the moderator is given in a separate column, followed by the associated number of studies in brackets.

**MODERATORS** *(AIC = 167.21)*	**ES lnOR (OR)**	**N_{ES} (studies)**	**SE**	**CI LOWER(OR)**	**CI UPPER (OR)**
Adult female*	-1.334 (0.263)	21 (10)	0.443	-2.202 (0.111)	-0.466 (0.627)
Adult male*	-1.107 (0.331)	16 (8)	0.444	-1.977 (0.138)	-0.237 (0.789)
Juvenile	-1.272 (0.280)	14 (7)	0.705	-2.654 (0.070)	0.110 (1.116)
Contrast (female-male)	-0.227 (0.797)	-	0.120	-0.462 (0.630)	0.007 (1.007)
Contrast (juv-male)	-0.165 (0.848)	-	0.833	-1.798 (0.166)	1.468 (4.339)
Contrast (juv-female)	0.062 (1.064)	-	0.833	-1.570(0.208)	1.694 (5.440)

QM(df = 2) = 3.6177, p-val = 0.1638

Table A2.S1_2. This is the source data to Figure 2a for the effect of "treatment agent" (replicating agent vs. non-replicating agent) on survival (*non-phylogenetic model*). Effect sizes used for statistical tests were logged OR (lnOR). Sample size of effect sizes for each group of the moderator is given in a separate column, followed by the associated number of studies in brackets.

**MODERATORS** *(AIC = 160.84)*	**ES lnOR (OR)**	**N_{ES} (studies)**	**SE**	**CI LOWER**	**CI UPPER**
Replic. agent*	-2.011 (0.134)	23 (11)	0.428	-2.648 (0.058)	-0.971 (0.309)
Non-replic. agent	-0.700 (0.497)	28 (13)	0.381	-1.447 (0.235)	0.047 (1.048)
Contrast (Replic. –Non-replic.)*	-1.312 (0.269)	--	0.484	-2.260 (0.104)	-0.363 (0.696)

QM(df = 1) = 7.3414, p-val = 0.0067

Table A2.S2_1. This is the source data to Figure 2b for the effect of “life-history status” on reproduction (*non-phylogenetic model*). Since the majority of studies only assigned sex to adult individuals, sex and age were combined into one moderator consisting of three levels: adult females, adult males, and juveniles. Effect sizes used for statistical tests were Hedges’ *g*. Sample size of effect sizes for each group of the moderator is given in a separate column, followed by the associated number of studies in brackets.

**MODERATORS** *(AIC = 120.18)*	**ES (H*g*)**	**N_{ES} (studies)**	**SE**	**CI LOWER**	**CI UPPER**
*Adult female**	*-0.199*	*58(24)*	*0.080*	*-0.356*	*-0.042*
Adult male	0.068	10 (4)	0.133	-0.193	0.329
Juvenile	0.098	12 (4)	0.208	-0.310	0.506
Contrast (female-male)*	*-0.267*	-	*0.122*	*-0.506*	*-0.028*
Contrast (juv-male)	0.030	-	0.247	--0.454	0.514
Contrast (juv-female)	0.297	-	0.223	-0.140	0.734

QF (df = 2) = 6.2256, (p-val) = 0.0445

Table A2.S2_2. This is the source data to Figure 2b for the effect of “treatment agent” (replicating agent vs. non-replicating agent) on reproduction (*non-phylogenetic model*). Effect sizes used for statistical tests were Hedges’ *g*. Sample size of effect sizes for each group of the moderator is given in a separate column, followed by the associated number of studies in brackets.

**MODERATORS** *(AIC = 122.24)*	**ES (H*g*)**	**N_{ES} (studies)**	**SE**	**CI LOWER**	**CI UPPER**
*Replic. agent**	*-0.300*	*18 (7)*	*0.137*	*-0.568*	*-0.032*
Non-replic. agent	-0.067	62 (22)	0.085	-0.234	0.101
Contrast (Replic. –Non-replic.)	-0.234	--	0.161	-0.550	0.082

QF (df = 1) = 2.1044, p-val = 0.1469

Table A2.S3_1. This is the source data to Figure 2c for the effect of "life-history status" on immune trait expression (*non-phylogenetic model*). Since the majority of studies only assigned sex to adult individuals, sex and age were combined into one moderator consisting of three levels: adult females, adult males, and juveniles. Effect sizes used for statistical tests were Hedges' *g*. Sample size of effect sizes for each group of the moderator is given in a separate column, followed by the associated number of studies in brackets.

**MODERATORS** *(AIC = 162.20)*	**ES (H*g*)**	**N_{ES} (studies)**	**SE**	**CI LOWER**	**CI UPPER**
*Adult female**	*0.880*	*24 (9)*	*0.406*	*0.085*	*1.675*
Adult male*	*0.843*	*20 (7)*	*0.416*	*0.029*	*1.658*
Juvenile	0.732	10 (4)	0.660	-0.562	2.025
Contrast (female-male)	0.037	--	0.234	-0.423	0.495
Contrast (juv-male)	-0.112	--	0.780	-1.640	1.417
Contrast (juv-female)	-0.148	--	0.775	-1.667	1.370

QF (df = 2) = 0.0541, p-val = 0.9733

Table A2.S3_2. This is the source data to Figure 2c for the effect of "treatment agent" (replicating agent vs. non-replicating immune agent) on immune trait expression (*non-phylogenetic model*). Effect sizes used for statistical tests were Hedges' *g.* Sample size of effect sizes for each group of the moderator is given in a separate column, followed by the associated number of studies in brackets.

**MODERATORS** *(AIC = 160.25)*	**ES (H*g*)**	**N_{ES} (studies)**	**SE**	**CI LOWER**	**CI UPPER**
*Replic. agent**	*0.822*	*14 (4)*	*0.380*	*0.078*	*1.566*
*Non-replic. Agent**	*0.826*	*40 (14)*	*0.327*	*0.185*	*1.468*
Contrast (Replic. –Non-replic.)	-0.004	--	0.248	-0.490	0.482

QM (df = 1) = 0.0003, p-val = 0.9859

Table A2.S4_1. This is the source data to Figure 2d for the effect of “life-history status” on morphology (*non-phylogenetic model*). Since the majority of studies only assigned sex to adult individuals, sex and age were combined into one moderator consisting of three levels: adult females, adult males, and juveniles. Effect sizes used for statistical tests were Hedges’ *g*. Sample size of effect sizes for each group of the moderator is given in a separate column, followed by the associated number of studies in brackets.

**MODERATORS** *(AIC = 65.00)*	**ES (Hg)**	**N_{ES} (studies)**	**SE**	**CI LOWER**	**CI UPPER**
Adult female	0.276	7 (5)	0.248	-0.209	0.761
Adult male	0.104	5 (4)	0.284	-0.662	0.453
Juvenile	-0.028	19 (6)	0.176	-0.372	0.317
Contrast (fem-male)	0.381	--	0.377	-0.358	1.119
Contrast (juv-male)	0.077	--	0.334	-0.579	0.732
Contrast (juv-female)	-0.304	--	0.304	-0.899	0.291

QM(df = 2) = 1.3166, p-val = 0.5177

Table A2.S4_2. This is the source data to Figure 2d for the effect of "treatment agent" (replicating agent vs. non-replicating agent) on morphology (*non-phylogenetic model*). Effect sizes used for statistical tests were Hedges' *g.* Sample size of effect sizes for each group of the moderator is given in a separate column, followed by the associated number of studies in brackets.

**MODERATORS** *(AIC = 70.52)*	**ES (Hg)**	**N_{ES} (studies)**	**SE**	**CI LOWER**	**CI UPPER**
Replic. agent	-0.095	12 (3)	0.241	-0.567	0.377
Non-replic. Agent	0.095	19 (12)	0.153	-0.204	0.395
Contrast (Replic. –Non-replic.)	-0.190		0.285	-0.749	0.369

QM(df = 1) = 0.4444, p-val = 0.5050

Table A2.S5_1. This is the source data to Figure 2e for the effect of “treatment agent” (replicating agent vs. non-replicating agent) on development times (*non-phylogenetic model*). Effect sizes used for statistical tests were Hedges’ *g.* Sample size of effect sizes for each group of the moderator is given in a separate column, followed by the associated number of studies in brackets.

**MODERATORS** *(AIC = 28.44)*	**ES (Hg)**	**N_{ES} (studies)**	**SE**	**CI LOWER**	**CI UPPER**
Replic. agent	0.208	5 (3)	0.291	-0.363	0.779
Non-replic. Agent	-0.264	13 (3)	0.2561	-0.766	0.239
Contrast (Replic. –Non-replic.)	0.274	--	0.226	-0.169	0.718

QM(df = 1) = 1.4723, p-val = 0.2250

Tables associated with Figure 3 - meta-regressions

Table A2.S6. This is the source data to Figure 3a for the effect of all moderators on survival (*non-phylogenetic model*). Effect sizes used for statistical tests were logged OR, also displaying back-transformed lnOR in brackets. Reference level for the full model is adult males that were challenged with a non-replicating agent. AIC-values are generated from a ML model.

**MODERATORS** *(AIC = 161.75)*	**ES lnOR (OR)**	**SE**	**CI LOWER (OR)**	**CI UPPER (OR)**
Life-history status				
Adult females	-0.225 (0.799)	0.119	-0.459 (0.632)	0.010 (1.010)
Juveniles	0.317(1.373)	0.770	-1.192 (0.304)	1.825 (6.205)
Treatment agent				
*Replicating agent**	*-1.354 (0.258)*	*0.492*	*-2.318 (0.099)*	*-0.389 (0.678)*

QM(df = 3) = 10.8987, p-val = 0.0123

Table A2.S7. This is the source data to Figure 3b for the effect of all moderators on reproduction (*non-phylogenetic model*). Effect sizes used for statistical tests were Hedges' *g*. The reference level in the full model reflects adult females that were challenged with a non-replicating agent. AIC-values are generated from a ML model.

**MODERATORS** (*AIC = 120.94*)	**ES (H*g*)**	**SE**	**CI LOWER**	**CI UPPER**
Life-history status				
*Adult females**	*-0.261*	*0.122*	*-0.500*	*-0.021*
Juveniles	-0.017	0.256	-0.519	0.485
Treatment agent				
Replicating agent	-0.187	0.173	-0.526	0.153

Table A2.S8. This is the source data to Figure 3c for the effect of all moderators (main effects only) on immune trait expression (*non-phylogenetic model*). Effect sizes used for statistical tests were Hedges' *g*. The reference level in the full model reflects adult females that were challenged with a non-replicating agent. AIC-values are generated from a ML model.

**MODERATORS** *(AIC = 164.20)*	**ES (H*g*)**	**SE**	**CI LOWER**	**CI UPPER**
**Life-history status**				
Adult females	0.035	0.239	-0.434	0.503
Juveniles	-0.110	0.789	-1.656	1.436
**Treatment agent**				
Replicating agent	0.002	0.255	-0.499	0.503

QM (df = 3) = 0.0490, p-val = 0.9972

Table A2.S9. This is the source data to Figure 3d for the effect of all moderators on morphology (*non-phylogenetic model*). Effect sizes used for statistical tests were Hedges' *g*. The reference level in the full model reflects adult females that were challenged with a non-replicating agent. AIC-values are generated from a ML model.

**MODERATORS** *(AIC = 73.35)*	**ES (H*g*)**	**SE**	**CI LOWER**	**CI UPPER**
Life-history status				
Adult females	0.349	0.404	-0.443	1.140
Juveniles	0.081	0.350	-0.606	0.768
Treatment agent				
Replicating agent	-0.073	0.324	-0.708	0.563

QM(df = 3) = 1.2123, p-val = 0.7501

Tables associated with Figure 4 - meta-regressions

Subsample for MATING STATUS

Table A2.S10. This is the source data to Figure 4a for the effect of "mating status" (virgin or mated) on survival. Effect sizes used for statistical tests were logged OR (lnOR). Sample size of effect sizes for each group of the moderator is given in a separate column, followed by the associated number of studies in brackets. Note that all data is from invertebrates.

**MODERATORS** *(AIC = 63.76)*	**ES lnOR (OR)**	**N_{ES} (studies)**	**SE**	**CI LOWER (OR)**	**CI UPPER (OR)**
Mating status					
Virgin	0.085 (1.240)	12 (5)	0.554	-0.100 (0.579)	1.170 (2.657)
*Mated**	*-0.933 (0.374)*	*17 (8)*	*0.364*	*-1.646 (0.197)*	*-0.219 (0.710)*
Contrast (mated-virgin)	*-1.018 (0.361)*	--	*0.663*	*-2.316 (0.099)*	*-0.281 (1.324)*

QM (df = 1) = 2.359, p-val = 0.125

Table A2.S11. This is the source data to Figure 4b for the efffect of “mating status” (virgin or mated) on reproduction. Effect sizes used for statistical tests were Hedges’ g. Sample size of effect sizes for each group of the moderator is given in a separate column, followed by the associated number of studies in brackets. Note the there was only enough data to include invertebrate females in this analysis.

**MODERATORS** *(AIC = 56.12)*	**ES(H*g*)**	**N_{ES} (studies)**	**SE**	**CI LOWER (H*g*)**	**CI UPPER (H*g*)**
Mating status					
Virgin	-0.627	12 (5)	0.272	-1.160	-0.093
Mated	-0.028	28 (9)	0.182	-0.385	0.329
Contrast (mated-virgin)	0.599	--	0.328	-0.043	1.241

QM (df = 1) = 3.3416, p-val = 0.0675

Table A2.S12. This is the source data to Figure 4c for the effect of "mating status" (virgin or mated) on immune trait expression. Effect sizes used for statistical tests were Hedges' g. Sample size of effect sizes for each group of the moderator is given in a separate column, followed by the associated number of studies in brackets.

**MODERATORS** *(AIC = 53.40)*	**ES(H*g*)**	**N_{ES} (studies)**	**SE**	**CI LOWER (H*g*)**	**CI UPPER (H*g*)**
Mating status					
Virgin	0.324	20 (5)	0.306	-0.275	0.923
Mated	0.617	9 (2)	0.353	-0.076	1.309
Contrast (mated-virgin)	0.293	--	0.252	-0.202	0.787

QM (df = 1) = 1.3447, p-val = 0.2462
